# Can tiered open-source mandates optimize innovation? A differential game study osn platform vs. independent project dynamics in foundation software ecosystems

**DOI:** 10.1371/journal.pone.0340713

**Published:** 2026-09-11

**Authors:** Bingyang Wu, Yongxi Yi

**Affiliations:** 1 School of Management Science, Dhonburi Rajabhat University, Dhonburi, Thailand; 2 School of Technology and Business, Hunan University of Information Technology, Changsha, China; Wenzhou-Kean University, CHINA

## Abstract

The governance of AI foundation software ecosystems requires balancing platform scale and independent innovation to ensure technological sovereignty. This paper examines whether tiered open-source mandates—with differentiated requirements for platforms and independents—can optimize ecosystem innovation. We develop and solve a tripartite Stackelberg differential game model incorporating differences in compliance costs, ecosystem dependence, and appropriation capabilities. Analysis shows that tiered mandates outperform uniform ones when heterogeneity exceeds threshold levels, optimally imposing stricter rules on platforms ($\theta_{D}\;\in [0.60,0\mathrm{.75}]$) and offering flexibility to independents ($\theta_{I}\;\in [0.25,0\mathrm{.40}]$). Ecosystem health improvements range from 15–40%, peaking under high compliance cost asymmetry ($\lambda_{D}\;/\lambda_{I}\;>2.0$). Key boundary conditions are identified, and an adaptive decision framework is proposed. This research formalizes differentiated mandates as an endogenous policy variable, quantifies dominance conditions, and provides actionable thresholds, demonstrating that calibrated differentiation based on structural heterogeneity can optimize innovation in AI infrastructure governance. Throughout this paper, “health improvement” refers specifically to the ecosystem architect’s composite health function $H\left(K_{D}\;,K_{I}\;\right)$, a governance metric of scale, diversity, and balance, not a full measure of social welfare.

## 1. Introduction

As the core engine of the Fourth Industrial Revolution, AI is reshaping global economic landscapes and redefining the basis of national technological sovereignty. Its advancement transcends mere algorithmic progress, resting fundamentally on a robust, secure, and autonomous software infrastructure, the AI foundation software stack. This stack, encompassing operating systems, development frameworks, compilers, and toolchains optimized for AI workloads, constitutes the “digital bedrock” of the intelligent era. Mastery and influence over this stack directly determine a nation’s or firm’s strategic autonomy in data processing, model innovation, and ecosystem governance, thereby shaping long-term industrial competitiveness and security [1, 2].

The AI software stack exhibits distinctive features that differentiate it from general-purpose software ecosystems: extreme network effects, where each additional compatible tool exponentially increases platform value; deep hardware-software coupling, where frameworks are optimized for specific accelerators; and geopolitical salience, where control over the stack confers strategic advantage. These features amplify the governance challenges we analyze and motivate our focus on this specific context.

However, the development architecture of the global AI software stack reveals a critical tension. On one hand, dominant platform projects, often backed by prominent technology corporations, possess unparalleled resources, integrated developer ecosystems, and clear pathways for commercial monetization. They drive standardization and provide stable, scalable foundations. On the other hand, independent open-source projects, spearheaded by agile startups, academic institutions, or community collectives, serve as vital engines of experimentation, architectural diversity, and disruptive innovation. They challenge incumbents and prevent ecosystem lock-in. This co-existence of heterogeneous development models—the resource-rich, integrated “platform” model and the agile, community-centric “independent” model—is a defining feature of the open-source AI ecosystem. Nevertheless, it also presents a fundamental governance challenge: how can ecosystem architects (e.g., foundations, consortia, or policymakers) design rules that simultaneously harness the scaling power of platforms and preserve the innovative vitality of independents?

This challenge is acutely felt in the context of open-source mandates—policies that require contributors to release a portion of their code under open licenses. Such mandates are increasingly deployed as strategic tools to build commons, ensure interoperability, and accelerate ecosystem growth. However, a blunt, one-size-fits-all mandate may be profoundly suboptimal. Imposing a uniformly high open-source requirement could stifle the commercial incentives that drive sustained platform investment. In contrast, a uniformly low requirement might allow platforms to privatize foundational knowledge too much, undermining the collaborative commons. Thus, the pivotal yet underexplored policy question emerges: Could a tiered open-source mandate, differentiating requirements by developer type (platform vs. independent), strike a superior balance, optimizing innovation output and the long-term health of the entire ecosystem?

Despite the practical urgency, rigorous theoretical analysis of this question is scarce. Existing literature richly describes platform-community dynamics and the economics of open source, but it treats policy tools as exogenous or uniform. There is a lack of dynamic, strategic models that endogenize the design of differentiated openness policies and analyze their equilibrium effects on the co-evolution of heterogeneous contributors. This gap limits policymakers’ ability to move beyond intuition towards evidence-based governance design.

While the general principle that heterogeneous agents warrant differentiated policy treatment is well-established in mechanism design and nonlinear pricing theory [3]; [4], its application to dynamic open-source governance, where policy instruments interact endogenously with R&D investment over time and where knowledge spillovers create non-convexities, remains unformalized. Our contribution is not to rediscover that differentiation helps, but to derive state-contingent feedback mandate functions in a dynamic setting, quantify how network effect strength and spillover asymmetry modulate the optimal degree of differentiation, and provide testable boundary conditions specific to the governance of AI infrastructure ecosystems.

To address this gap, this paper formulates and solves a tripartite Stackelberg differential game model involving an Ecosystem Architect (A), a Dominant Platform Project (D), and an Independent Open-Source Project (I). The model captures the intrinsic heterogeneities between D and I, such as differential compliance costs, varying reliance on the ecosystem, and asymmetric capabilities for private appropriation. The Architect, as the leader, chooses between a uniform open-source contribution rate and a tiered (differentiated) rate structure. The projects, as followers, then engage in continuous-time competition and collaboration, dynamically adjusting their R&D efforts to maximize their respective payoffs, which incorporate benefits from both the shared knowledge commons and private returns.

The core research question we address is: Can a tiered open-source mandate, by strategically discriminating between platform and independent projects, demonstrably outperform a uniform mandate in maximizing the overall health and innovative capacity of the AI software ecosystem? We pursue this question through a structured analytical framework: first, deriving feedback equilibrium strategies under a uniform mandate (baseline); second, solving for the equilibrium under a tiered mandate (intervention); and finally, conducting a systematic, quantitative comparison of the resulting ecosystem trajectories. Our key metrics for comparison include the total accumulation of open-source knowledge, the dynamic balance between the two developer types, and the present-discounted value of a comprehensive ecosystem health function.

Our findings provide formal, mathematical support for the targeted use of tiered mandates. We demonstrate that a well-calibrated tiered policy can induce a superior dynamic equilibrium, directing platform resources toward building high-impact commons while safeguarding the independent project’s innovative space. This leads to a net increase in ecosystem-scale innovation and long-term robustness. Crucially, we identify the boundary conditions, such as the degree of synergy within platform projects, the disparity in compliance costs, and the strength of network effects, under which the tiered approach offers the most significant health advantage.

The subsequent sections go as follows: Section 2 reviews the related literature. Section 3 details the differential game model. Section 4 presents the equilibrium solution method. Section 5 provides numerical simulations and policy analysis. Section 6 discusses robustness and boundary conditions. Section 7 concludes with implications and future research directions.

## 2. Literature review

This section provides a systematic review of the scholarly foundations relevant to our study. We examine three interconnected streams of research: (1) the economics and governance of open-source ecosystems, with a focus on platform-community dynamics; (2) the application of game theory to analyze standards competition, platform strategy, and open innovation; and (3) policy evaluations of digital innovation governance, particularly regarding open-source regulation. Building on this synthesis, we identify the critical research gap that this paper aims to address and delineate its specific contributions.

### 2.1. The Economics and Governance of Open-Source Ecosystems

(1) The open-source software (OSS) paradigm has evolved from a niche movement to the dominant mode of production for critical digital infrastructure. A substantial body of work examines its economic logic, governance structures, and sustainability.

Early research focused on the motivations for participation, challenging traditional economic models by highlighting intrinsic drivers like ideological commitment, peer recognition, and learning [5, 6]. This has matured into a nuanced understanding of heterogeneous contributor profiles within complex ecosystems, where corporate employees and community volunteers interact [7].

A central theme in recent literature is the tension and synergy between corporate-sponsored platforms and community-driven projects. Major technology firms have become the dominant contributors to critical OSS projects, leading to “corporate-open-source” hybrid models [8]. Studies examine how firms strategically use open source to commoditize complements, build de facto standards, and harness external innovation [9–11]. Concurrently, a vibrant stream of research analyzes ecosystem governance, exploring how foundations (e.g., Apache, Linux), licensing schemes, and contribution workflows manage conflicts, ensure quality, and sustain participation [12–14].

Crucially, scholars have conceptualized software stacks as multi-layered platforms, where competition occurs not just between products but between integrated ecosystems [15]. The AI foundation software stack is a prime example of such a layered, platform-driven market. Research highlights the “winner-takes-most” dynamics driven by network effects and the strategic challenges for new entrants [16]. However, while this literature richly describes the structural forces and actor strategies, it predominantly treats governance policies, such as specific open-source mandates, as external, static factors. There is a paucity of work that dynamically models how differentiated governance rules endogenously shape the strategic investment and interaction between heterogeneous actor types (like integrated platforms vs. agile independents) over time. Our study aims to inject this dynamic, strategic, and policy-endogenous perspective into the ecosystem literature.

### 2.2. Game Theory in Platform Competition and Open Innovation

Game theory provides a powerful analytical toolkit for modeling strategic interactions in technology markets, particularly those characterized by network effects, path dependency, and co-opetition.

In the context of standards and platform wars, game-theoretic models have been instrumental. Static models analyzed adoption decisions, compatibility choices, and the pivotal role of installed bases [17]. Dynamic and differential game models extended this analysis to capture temporal evolution, treating market share or technology quality as state variables and R&D/pricing as control variables [18]. These models excel at illustrating long-term equilibrium paths under network externalities and R&D spillovers.

Applied to open-source strategy, game theory has been used to examine firms’ decisions to contribute to or adopt OSS. Models analyze trade-offs between proprietary control and the benefits of open collaboration, such as cost-sharing, faster adoption, and preempting rivals [19]; [20]. Particularly relevant to our work are models of ecosystem co-opetition, where firms simultaneously cooperate on a shared platform (e.g., through open-source contributions) while competing in downstream markets [21]. These models reveal how complementary and competitive interactions affect innovation incentives and profit distribution.

Despite these advances, existing game-theoretic literature exhibits two key limitations that our research directly tackles. First, it often models participants as atomic, homogeneous firms, failing to capture the fundamental structural and incentive heterogeneities between a large, integrated platform project and a small, independent one. Treating them as symmetric players misses the core policy dilemma. Second, policy interventions in these models are typically simplistic, often a binary choice or a one-dimensional subsidy. They rarely incorporate the nuanced, multi-tiered design of open-source governance instruments (e.g., tiered contribution mandates) as endogenous strategic variables that a social planner or platform leader can optimize. Our model explicitly centers this heterogeneity and policy endogeneity.

### 2.3. Policy Evaluation and Governance of Digital Innovation

Governments and foundations increasingly employ a range of policy tools to steer digital innovation, moving beyond traditional R&D subsidies to include data governance, procurement rules, and direct support for open-source communities.

Empirical evaluations of such policies often leverage econometric methods or case studies to assess impacts on outcomes like code contribution volume, project forks, or startup formation around OSS [22]. However, establishing causality is fraught with challenges due to endogeneity and the complexity of ecosystems.

Theoretical modeling, employing principal-agent frameworks or game theory, allows for analyzing policy mechanisms under controlled assumptions. This stream has investigated optimal software licensing [23], the design of crowdsourcing mechanisms like bug bounties [24], and the impact of patent policies on open innovation [25,26]. A growing subfield examines “open-source by mandate” or “public code” policies, which require software developed with public funds to be released as OSS. Scholars debate their efficacy, noting potential benefits in transparency and collaboration but also risks of low-quality “compliance dumping” and reduced commercial investment incentives [22, 27].

A critical and unresolved gap in this policy literature is the lack of rigorous analysis of differentiated mandates. The debate and existing models overwhelmingly focus on uniform policies (e.g., “all publicly funded software must be open-sourced”). There is scant theoretical exploration of whether and why a tiered mandate, which imposes different obligations based on the type of developer, project scale, or software layer, could yield superior societal outcomes. This represents a significant blind spot in the theory of digital innovation policy design, which our study aims to illuminate.

### 2.4. Research Gaps and This Study‘s Contributions

Synthesizing the above, a salient and interdisciplinary research gap emerges. There is a lack of an integrated dynamic strategic framework that simultaneously: (1) models the realistic co-opetition between structurally heterogeneous entities (dominant platform vs. independent project) within a layered software ecosystem; (2) treats the detailed design space of open-source governance instruments, specifically the choice between uniform and tiered mandates, as a core, endogenous policy variable to be optimized; and (3) analyzes how an ecosystem architect can strategically employ this instrument to steer the ecosystem towards superior innovation and health outcomes.

Our approach is related to, but distinct from, the classical literature on optimal taxation and nonlinear pricing [3]; [28]; [29]. In those models, a principal designs a menu of contracts or tax schedules to screen a continuum of agent types in a static or repeated setting. Our contribution differs in three ways: (i) the policy instrument is a dynamic feedback rule rather than a static schedule; (ii) agents’ types are exogenously fixed and observable (platform vs. independent) rather than screened; and (iii) the key friction is knowledge spillovers and strategic R&D interaction, not private information. This makes our model complementary to, rather than a subset of, classical regulation theory.

This study makes three primary contributions to bridge this gap:

(1) Theoretical Model Development: We construct a novel tripartite Stackelberg differential game model that formally incorporates the intrinsic heterogeneities between a dominant platform project and an independent open-source project. This provides a dedicated analytical framework for studying the dynamic interplay between different innovation models under proactive ecosystem governance.
(2) Endogenization of Differentiated Policy: For the first time in this context, we formally model, solve, and systematically compare the feedback equilibria under two distinct governance regimes: a uniform mandate versus a tiered mandate. This approach endogenizes the selection and calibration of a sophisticated class of digital innovation policies, moving beyond treating them as exogenous shocks.
(3) Quantified Policy Insights and Boundary Conditions: Through numerically simulated comparative dynamics, we quantify the potential ecosystem health advantages of a tiered mandate. More importantly, we clearly delineate the economic and technological boundary conditions (e.g., the degree of compliance cost asymmetry, the strength of network effects) under which it is most effective. This offers nuanced, actionable, and evidence-based guidance for policymakers and foundation leaders navigating the complex trade-offs in governing open-source ecosystems for strategic technologies like the AI software stack.

Furthermore, the novelty of our dynamic Stackelberg framework lies in three specific theoretical insights that static mechanism design cannot produce. First, we derive state-contingent feedback mandate rules rather than static menus—the optimal openness requirement depends on the current knowledge stocks, implying that governance should adapt as the ecosystem evolves. Second, we identify dynamic amplification mechanisms: network effects and knowledge spillovers generate virtuous cycles under tiered mandates (higher platform contributions → larger commons → stronger independent innovation → greater platform value) that are absent in static models. Third, we characterize non-monotonic boundary conditions (e.g., the inverted-U relationship between compliance cost asymmetry and health gain), revealing that optimal differentiation is bounded—too little heterogeneity offers no scope for tiering, while too much heterogeneity overwhelms the platform’s absorptive capacity. These dynamic and non-linear features constitute the distinctive theoretical contribution beyond the general principle that heterogeneity justifies differentiation.

## 3. Model Construction

This paper develops a three-player Stackelberg differential game to analyze the strategic interplay within an AI foundation software ecosystem. The model comprises: an Ecosystem Architect (A), a Dominant Platform Project (D), and an Independent Open-Source Project (I). This setup captures the hierarchical governance and co-opetitive dynamics inherent in modern open-source ecosystems, where a foundational authority sets rules that shape the competitive and collaborative efforts of large, integrated platforms and smaller, agile independents. All interactions occur over a continuous, infinite time horizon.

To address our core research question, we analyze and compare two distinct policy regimes designed by the Architect:

Scenario 1 (Baseline Policy): Uniform Open-Source Mandate. The architect enforces a single, state-dependent open-source contribution requirement, $\theta \left(K_{D},K_{I}\right)\in \left[0,1\right]$, applied equally to both D and I. A value of θ=0.6 signifies that 60% of all new code developed by an entity must be released under an approved open-source license.

Scenario 2 (Intervention Policy): Tiered Open-Source Mandate. The architect implements differentiated requirements: $\theta_{D}\left(K_{D},K_{I}\right)$ for the platform project and $\theta_{I}\left(K_{D},K_{I}\right)$ for the independent project, where $\theta_{D}\neq \theta_{I}$. The policy design space typically expects $\theta_{D}>\theta_{I}$, reflecting a strategic choice to impose a greater commons-building obligation on the resource-rich platform.

### 3.1. Model Assumptions and Parameter Setting

(1) Architectural Layers and Knowledge Spillovers: The AI software stack is conceptualized as having foundational layers (e.g., core frameworks, compilers) and value-added layers. Code released as open-source contributes to a public knowledge commons, generating positive spillovers ($\gamma_{ij}$) that enhance the productivity of all ecosystem participants. The effectiveness of spillovers may differ based on the source and recipient.

(2) Entity Heterogeneity: The model’s core is the structural difference between D and I:

Dominant Platform Project (D): Characterized by greater resources, integrated development environments, and established commercial pathways. This translates to a higher potential for private appropriation of non-open-sourced code ($\rho_{D}$) but also higher costs for mandate compliance ($\lambda_{D}$) due to complex internal governance and intellectual property management.

Independent Open-Source Project (I): Characterized by agility, strong community reliance, and niche expertise. It derives greater marginal benefit from ecosystem participation and reputation ($\beta_{I}$) but has more limited private monetization potential ($\rho_{I}$) and lower compliance costs ($\lambda_{I}$).

We define “platformness” (the essential trait distinguishing D from I) as the combination of (i) higher private appropriation capacity ($\rho_{D}>\rho_{I}$), and (ii) higher compliance cost elasticity ($\lambda_{D}>\lambda_{I}$). These two features are conceptually linked: a platform’s larger scale and deeper commercial integration simultaneously create greater monetization opportunities and higher organizational friction in open-sourcing code. The other parameter differences (β, η, γ) are secondary consequences or moderators, not defining traits. We conduct counterfactual analysis in Section 6.3.1 where only the defining traits vary and others are held equal, confirming that these two dimensions are the primary drivers of the tiering advantage.

We acknowledge that the binary classification of projects as “platform” versus “independent” is a simplification. In reality, projects such as Hugging Face occupy intermediate positions, combining venture capital backing with strong community engagement. Our binary model can be understood as the limiting case of a continuum where the two types represent poles of the distribution. Extending the model to a continuous type space is a promising direction for future work (see Section 7.3); however, the binary formulation allows us to derive sharp analytical insights about the structural conditions under which differentiation is most valuable.

(3) Knowledge as a Dynamic Stock: Innovation is modeled as the accumulation of effective open-source knowledge. An entity’s contributions to this commons are determined by its R&D effort, its efficiency, and the mandated open-source rate. Knowledge depreciates at rate δ, capturing rapid technological obsolescence.
(4) Ecosystem Health as the Policy Objective: The Architect’s goal is not to maximize the output of a single entity but to optimize the long-term health of the overall ecosystem. Health is a multi-dimensional metric encompassing the total scale of the knowledge commons, the diversity of contributors (preventing monopolization), and the system’s robustness.

### 3.2. State Variables and State Dynamics

The system’s state is defined by the Effective Open-Source Knowledge Stocks of the two developer types, $K_{D}\left(t\right)$ and $K_{I}\left(t\right)$. These stocks represent the accumulated, openly accessible, and technically relevant code that forms the shared foundation of the ecosystem.

Knowledge Stock of the Dominant Platform Project ($K_{D}$):


$$\begin{array}{c} \frac{dK_{D}\;\left(t\right)}{dt}=\eta_{D}\;\cdot \theta_{D}\;e_{D}\;\left(t\right)-\delta K_{D}\;\left(t\right),K_{D}\;\left(0\right)=K_{D0}\;\geq 0 \end{array}$$ (1)

Here, $\eta_{D}$ is the R&D efficiency coefficient of the platform, and $\;e_{D}\;\left(t\right)\geq 0$ is its R&D effort level. The term $\eta_{D}\;\cdot \theta_{D}\;e_{D}\;\left(t\right)$ represents the flow of new open-source knowledge generated by D.

Knowledge Stock of the Independent Project ($K_{I}$):


$$\begin{array}{c} \frac{dK_{I}\;\left(t\right)}{dt}=\eta_{I}\;\cdot \theta_{I}\;e_{I}\;\left(t\right)\geq 0-\delta K_{I}\;\left(t\right),K_{I}\;\left(0\right)=K_{I0}\;\geq 0 \end{array}$$ (2)

The parameters $\eta_{I}$ and $\;e_{I}\;\left(t\right)$ are defined analogously for the independent project.

### 3.3. Objective Functions

#### 3.3.1. Follower Objectives: Platform and Independent Project.

Each follower seeks to maximize the discounted present value of its net lifetime payoff, weighing benefits from the ecosystem against private returns and R&D costs.

**Dominant Platform Project (**D): Its instantaneous payoff includes: (i) benefits from accessing the total knowledge commons (own and spilled-over knowledge), (ii) revenue from privatizing a portion $1-\theta_{D}$ of its output, minus (iii) convex R&D costs amplified by compliance.


$$\begin{array}{c} \Pi_{D}=\alpha_{D}\left[\gamma_{DD}K_{D}\;\left(t\right)+\gamma_{ID}K_{I}\;\left(t\right)\right]+\rho_{D}(1-\theta_{D})\;e_{D}\;\left(t\right)-\frac{\text{1}}{\text{2}}c_{D}(1+\lambda_{D}\theta_{D})\;e_{D}\;(t{)}^{\text{2}} \end{array}$$ (3)

where $\gamma_{DD}<1$ represents the residual benefit the platform derives from its own past contributions (e.g., reputation, internal reuse efficiency), and $\gamma_{ID}$ represents the spillover from the independent project’s knowledge. This formulation avoids the double-counting concern whereby a firm would otherwise obtain credit for knowledge it had already paid to produce. $\alpha_{D}$ i s the marginal value of ecosystem knowledge to D, $\gamma_{ID}$ is the spillover coefficient from I to D, $\rho_{D}$ is the marginal private value of proprietary code, $c_{D}$ is the base R&D cost coefficient, and $\lambda_{D}$ scales the additional cost burden of the mandate.

**Independent Open-Source Project (**I): Its objective function is structurally similar but with parameters reflecting its distinct profile (e.g., higher $\beta_{I}$, lower $\rho_{I}$).


$$\begin{array}{c} \Pi_{I}=\alpha_{I}\left[\gamma_{II}K_{I}\;\left(t\right)+\lambda_{DI}K_{D}\;\left(t\right)\right]+\rho_{I}(1-\theta_{I})\alpha_{I}\;e_{I}\;\left(t\right)-\frac{\text{1}}{\text{2}}c_{I}(1+\lambda_{I}\theta_{I})\;e_{I}\;(t{)}^{\text{2}} \end{array}$$ (4)

#### 3.3.2. Leader Objective: The Ecosystem Architect.

The Architect selects the mandate policy to maximize the present value of aggregate Ecosystem Health, $H\left(K_{D}\;,K_{I}\;\right)$. We define instantaneous health $H\left(K_{D}\;,K_{I}\;\right)$ as a function of the state variables:


$$\begin{array}{c} H(K_{D}\;(t),K_{I}\;\left(t\right))=\alpha \left(K_{D}\;\left(t)+K_{I}\;(t\right)\right)+\beta \sqrt{K_{D}\;\left(t\right)K_{I}\;\left(t\right)}-\mu (K_{D}\;(t)-K_{I}\;(t){)}^{\text{2}} \end{array}$$ (5)

The quadratic term $-\mu (K_{D}\;(t)-K_{I}\;(t){)}^{\text{2}}$ directly penalizes imbalance between the two knowledge stocks, capturing the architect’s preference for a pluralistic ecosystem where neither type dominates excessively. In numerical implementation, we use a regularized version of the geometric mean term, $\sqrt{K_{D}\;\left(t\right)K_{I}\;\left(t\right)+\varepsilon }$ with $\varepsilon =10^{-6}$, to avoid boundary singularities when either stock approaches zero.

The objective function is:


$$\begin{array}{c} \underset{\theta_{u}or\left(\theta_{D},\theta_{I}\right)}{max}J_{G}=\int_{\text{0}}^{\infty }e^{-rt}H\left(K_{D}\;\left(t\right),K_{I}\;\left(t\right)\right)dt \end{array}$$ (6)

Scale Benefit (α): A larger total knowledge commons ($K_{D}\;\left(t\right)+K_{I}\;\left(t\right)$) increases network effects, attracts more developers, and raises the platform’s overall value.

Diversity Benefit (β): The geometric mean term $\sqrt{K_{D}\;\left(t\right)K_{I}\;\left(t\right)}$ captures the complementary value and resilience afforded by having multiple significant contributors. It is maximized when both stocks are positive and balanced, and approaches zero if either entity withdraws.

Imbalance Penalty (μ): The quadratic term penalizes extreme disparities between $K_{D}\;\left(t\right)$ and $K_{I}\;\left(t\right)$, which could signal unhealthy monopolization or the marginalization of one development model. This ensures the architect values a competitive, pluralistic ecosystem.

### 3.4. Game Sequence

The Stackelberg differential game proceeds as follows:

(1) At time t=0, the Ecosystem Architect (leader) commits to and announces a feedback strategy for the mandate. In Scenario 1, this is a single rule $\theta \left(K_{D},K_{I}\right)$. In Scenario 2, it is a pair of rules: $\theta_{D}\left(K_{D},K_{I}\right)$ and $\theta_{I}\left(K_{D},K_{I}\right)$.
(2) Having observed the Architect’s strategy, the followers (D and I) engage in a simultaneous non-cooperative Nash game. They choose their effort feedback strategies $e_{D}\left(K_{D},K_{I}\right)$ and $e_{I}\left(K_{D},K_{I}\right)$ to maximize their respective objective functions $\Pi_{D}$ and $\;\Pi_{I}$, taking the other’s strategy and the Architect’s rules as given.
(3) The chosen efforts drive the evolution of the knowledge stocks $K_{D}\;\left(t\right)$ and $K_{I}\;\left(t\right)$ according to the state dynamics (Equations 1 & 2).
(4) All players continuously adjust their strategies based on the observed state $\left(K_{D},K_{I}\right)$ in a closed-loop feedback manner throughout the infinite horizon.

This framework enables us to solve for and compare the subgame perfect feedback equilibria under the two governance regimes, thereby rigorously evaluating the impact of uniform versus tiered open-source mandates on the ecosystem’s dynamic trajectory.

## 4. Model Solution

This section employs dynamic programming and backward induction to solve the Stackelberg differential game model formulated. The solution procedure is conducted sequentially for the two policy scenarios: the uniform open-source mandate and the tiered open-source mandate. First, we derive the feedback Nash equilibrium for the followers, the Dominant Platform Project (D) and the Independent Project (I), conditional on a given mandate policy announced by the architect. Subsequently, with full anticipation of the followers’ reaction functions, we solve for the optimal dynamic mandate strategy of the leader, the Ecosystem Architect (A).

**Proposition 4.0** (Existence and Uniqueness). Under Assumptions 1–4 and the parameter restrictions specified in Appendix A.2, the system of HJB equations (7)–(11) admits a unique viscosity solution in the class of continuously differentiable functions on the compact state space $S=[0,\overset{―}{K}{]}^{\text{2}}$. The feedback strategies derived from the first-order conditions constitute a unique Markov-perfect equilibrium. Proof is provided in Appendix A.2, following the contraction mapping argument in Dockner et al. (2000, Chapter 7).

### 4.1. Follower Game: Optimal Effort Decisions

Given the architect’s mandate strategy $\theta_{i}\;\left(K_{D}\;,K_{I}\;\right)$ (where $\theta_{i}=\theta$ for Scenario 1, and $\theta_{i}=\left\{\theta_{D},\theta_{I}\right\}$ for Scenario 2) and taking the strategy of the other follower as given, each follower chooses its R&D effort level $e_{i}\left(t\right)$ to maximize its discounted payoff. The followers’ value functions, $V_{D}\left(K_{D},K_{I}\;\right)$ and $V_{I}(K_{D},K_{I}\;$, satisfy the Hamilton-Jacobi-Bellman (HJB) equations.

#### 4.1.1. Follower Decisions under Uniform Mandate Policy.

Under the uniform policy, both entities are subject to the same mandate rate θ.

HJB Equation for the Dominant Platform Project (D):


$$\begin{array}{c} rV_{D}=\underset{e_{D}\left(t\right)\geq 0}{max}\left\{\alpha_{D}\left[\gamma_{DD}K_{D}\;+\gamma_{ID}K_{I}\right]+\rho_{D}(1-\theta )\;e_{D}-\frac{\text{1}}{\text{2}}c_{D}(1+\lambda_{D}\theta )\;e_{D}^{\text{2}}+\frac{\partial V_{D}}{\partial K_{D}}\;{\dot{K}}_{D}+\frac{\partial V_{D}}{\partial K_{I}}{\dot{K}}_{I}\right\}\; \end{array}$$ (7)

where the state dynamics are ${\dot{K}}_{D}=\eta_{D}\theta \;e_{D}-\delta K_{D}$ and ${\dot{K}}_{I}=\eta_{I}\theta \;e_{I}^{*}-\delta K_{I}$, with $\;e_{I}^{*}$ representing the independent project’s optimal effort.

Taking the first-order condition (FOC) with respect to the control variable $e_{D}$ yields:


$$\begin{array}{c} \rho_{D}\;(1-\theta \;)\;-c_{D}\;(1+\lambda_{D}\;\theta \;)e_{D}\;+\frac{\partial V_{D}}{\partial K_{D}}\;\theta \;\eta_{D}\;=0 \end{array}$$

Solving for $e_{D}$ provides the platform’s optimal feedback effort rule under the uniform mandate:


$$\begin{array}{c} e_{D}^{*}\;\left(K_{D}\;,K_{I}\;\right)=\frac{\rho_{D}\;(1-\theta )\;+\theta \eta_{D}\;(\partial V_{D}/\partial K_{D})\;}{c_{D}\;(1+\lambda_{D}\;\theta_{u}\;} \end{array}$$

HJB Equation for the Independent Project (I):


$$\begin{array}{c} rV_{I}=\underset{e_{I}\left(t\right)\geq 0}{max}\left\{\alpha_{I}\left[\gamma_{II}K_{I}\;+\gamma_{DI}K_{D}\right]+\rho_{I}(1-\theta )\;e_{I}-\frac{\text{1}}{\text{2}}c_{I}(1+\lambda_{I}\theta )\;e_{I}^{\text{2}}+\frac{\partial V_{I}}{\partial K_{I}}{\dot{K}}_{I}+\frac{\partial V_{I}}{\partial K_{D}}{\dot{K}}_{D}\right\} \end{array}$$ (8)

The FOC with respect to $e_{I}$ is:


$$\begin{array}{c} \rho_{I}\;(1-\theta \;)\;-c_{I}\;(1+\lambda_{I}\;\theta )e_{I}\;+\frac{\partial V_{I}}{\partial K_{I}}\;\theta \;\eta_{I}\;=0 \end{array}$$

Thus, the independent project’s optimal feedback effort rule is:


$$\begin{array}{c} e_{I}^{*}\;\left(K_{D}\;,K_{I}\;\right)=\frac{\rho_{I}\;(1-\theta )\;+\theta \;\eta_{I}\;(\partial V_{I}/\partial K_{I})\;}{c_{I}\;(1+\lambda_{I}\;\theta )\;} \end{array}$$

#### 4.1.2. Follower Decisions under Tiered Mandate Policy.

Under the tiered policy, the two entities face different mandate rates, $\theta_{D}$ and $\theta_{I}$.

For the Dominant Platform Project (D): The HJB equation and solution procedure are analogous, but with $\theta_{D}$ replacing θ.


$$\begin{array}{c} e_{D}^{*}\;\left(K_{D}\;,K_{I}\;\right)=\frac{\rho_{D}\;(1-\theta_{D})\;+\theta_{D}\;\eta_{D}\;(\partial V_{D}/\partial K_{D})\;}{c_{D}\;(1+\lambda_{D}\;\theta_{D})\;} \end{array}$$

For the Independent Project (I): Similarly, with $\theta_{I}$ replacing θ.


$$\begin{array}{c} e_{I}^{*}\;\left(K_{D}\;,K_{I}\;\right)=\frac{\rho_{I}\;(1-\theta_{I})\;+\theta_{I}\;\eta_{I}\;(\partial V_{I}/\partial K_{I})\;}{c_{I}\;(1+\lambda_{I}\;\theta_{I})\;} \end{array}$$

**Proposition 4.1** (Follower Effort Decisions). In both policy regimes, an entity’s optimal R&D effort is:

(1) Positively related to the marginal value of its own knowledge stock ($V_{i,K_{i}}$)
(2) Positively related to its private appropriation incentive, net of the mandate ($\rho_{i}(1-\theta_{i})$).
(3) Negatively related to its effective marginal R&D cost, which is amplified by the mandate compliance burden ($c_{i}\;(1+\lambda_{i}\;\theta_{i}\;)$).
(4) The mandate rate $\;\theta_{i}\;$ exerts an ambiguous net effect on equilibrium effort. It creates a trade-off: a higher $\;\theta_{i}\;$ reduces the private appropriation term (disincentive) but increases the weight of the knowledge stock’s marginal value in the effort decision (incentive). The sign of the net effect depends on the entity‘s valuation of the future ecosystem ($V_{i,K_{i}}$) relative to its immediate private returns ($\rho_{i}$).

### 4.2. Leader Game: Optimal Mandate Decision-Making

As the Stackelberg leader, the architect anticipates the followers’ reaction functions $e_{D}^{*}\left(\theta \right)$, $e_{I}^{*}\left(\theta \right)$ and chooses the mandate policy to maximize the present value of ecosystem health $V_{A}\left(K_{D},K_{I}\right)$. The architect’s value function, $V_{A}\left(K_{D},K_{I}\right)$, satisfies its own HJB equation.

#### 4.2.1. Architect Decision under Uniform Mandate Policy.

Under the uniform policy, the architect chooses a single control variable θ. Its HJB equation is:


$$\begin{array}{c} rV_{A}=\underset{\theta \in \left[0,1\right]}{max}\left\{H\left(K_{D},K_{I}\right)+\frac{\partial V_{G}}{\partial K_{D}}{\dot{K}}_{D}+\frac{\partial V_{A}}{\partial K_{I}}{\dot{K}}_{I}\right\} \end{array}$$ (9)

Where ${\dot{K}}_{D}=\eta_{D}\theta \;e_{D}^{*}-\delta K_{D}$, ${\dot{K}}_{I}=\eta_{I}\theta \;e_{I}^{*}-\delta K_{I}$. The state dynamics incorporate the followers’ equilibrium efforts from $e_{D}^{t*}\;\left(K_{D}\;,K_{I}\;\right)$ and $e_{D}^{t*}\;\left(K_{D}\;,K_{I}\;\right)$.

Substituting the state dynamics and taking the first-order condition with respect to θ:


$$\begin{array}{c} \frac{\partial V_{A}}{\partial K_{D}}\frac{\partial {\dot{K}}_{D}}{\partial \theta }+\frac{\partial V_{A}}{\partial K_{I}}\frac{\partial {\dot{K}}_{I}}{\partial \theta }=0 \end{array}$$ (10)

The partial derivatives $\partial {\dot{K}}_{D}/\partial \theta$ and $\partial {\dot{K}}_{I}/\partial \theta$ account for both the direct effect of θ on the flow of knowledge and its indirect effect through changes in the followers’ optimal efforts ($\partial e_{D}^{*}/\partial \theta$, $\partial e_{I}^{*}/\partial \theta$).

Equation (10) implicitly defines the architect’s optimal feedback mandate rule under the uniform policy, $\theta^{*}\left(K_{D},K_{I}\right)$. It represents a balancing act: increasing θ directly boosts the open-source contribution rate from both entities but may suppress their effort incentives. The optimal rate equates the marginal benefit of increased commons-building (weighted by the shadow value of each knowledge stock) to the marginal cost of reduced effort.

#### 4.2.2. Architect Decision under Tiered Mandate Policy.

Under the tiered policy, the architect chooses two control variables, $\theta_{D}$ and $\theta_{I}$, separately. The HJB equation is:


$$\begin{array}{c} rV_{A}=\underset{\theta_{D},\theta_{I}\in \left[0,1\right]}{max}\left\{H\left(K_{D},K_{I}\right)+\frac{\partial V_{A}}{\partial K_{D}}{\dot{K}}_{D}+\frac{\partial V_{A}}{\partial K_{I}}{\dot{K}}_{I}\right\} \end{array}$$ (11)

Taking the first-order conditions with respect to each control variable yields a system of two equations:


$$\begin{array}{c} \frac{\partial {\dot{K}}_{D}}{\partial \theta_{D}}=0\begin{array}{cc} ; & \frac{\partial {\dot{K}}_{I}}{\partial \theta_{I}}=0 \end{array} \end{array}$$ (12)

Strictly speaking, the two FOCs remain coupled through the value function $V_{A}\left(K_{D},K_{I}\right)$ and its cross-partial derivatives. The statement that the architect “independently tunes” each mandate is a heuristic for the fact that the tiered regime has two control variables rather than one, allowing the planner to move in two directions in the policy space. The full feedback solution, obtained numerically, accounts for the coupling through the joint value function. We do not claim analytical separability.

Given that $\partial V_{A}/\partial K_{D}>0$ and $\partial V_{A}/\partial K_{I}>0$ (more knowledge improves ecosystem health), the optimality conditions in (12) essentially require:


$$\begin{array}{c} \frac{\partial {\dot{K}}_{D}}{\partial \theta_{D}}=0\begin{array}{cc} ; & \frac{\partial {\dot{K}}_{I}}{\partial \theta_{I}}=0 \end{array} \end{array}$$ (13)

subject to the constraints $\theta_{D},\theta_{I}\in \left[0,1\right]$. This implies that, under a tiered policy, the architect can independently tune each mandate to locally maximize the growth rate of each knowledge stock. For the platform project, $\theta_{D}^{*}$ is set to maximize ${\dot{K}}_{D}$, considering how $\theta_{D}$ affects $e_{D}^{*}$ via $e_{D}^{*}\;\left(K_{D}\;,K_{I}\;\right)$. A parallel logic applies to $\theta_{I}^{*}$ for the independent project.

**Proposition 4.2** (Architect Mandate Decision-Making). The architect’s optimal mandate strategy is state-dependent and qualitatively different across regimes:

Under a uniform policy, the single mandate $\theta^{*}$ represents a system-wide compromise. It cannot simultaneously maximize the growth of both $K_{D}$ and $K_{I}$ if their cost structures $\left(\lambda_{D},\lambda_{I}\right)$ and incentive profiles ($\rho_{D},\rho_{I}$) differ.

Under a tiered policy, the architect achieves targeted optimization. It can impose a relatively high $\theta_{D}^{*}$ on the platform project to leverage its resources for the commons, while setting a moderate $\theta_{I}^{*}$ for the independent project to preserve its innovative agility. This decoupled optimization is the key potential source of ecosystem health.

### 4.3. Numerical Solution Algorithm for Feedback Equilibrium

The systems of coupled, nonlinear partial differential equations (the HJB equations for $V_{D}$, $V_{I}$, $V_{G}$ along with the FOCs) are analytically intractable. Therefore, we employ a numerical Policy Iteration Algorithm (PIA) to solve for the Markov-perfect feedback equilibrium. The algorithm proceeds as follows:

(1) State Space Discretization: Define a bounded, discretized grid for the state variables: $K_{D}\in \left[0,\overset{―}{K}\right]$, $K_{I}\in \left[0,\overset{―}{K}\right]$ with step size ΔK.
(2) Initialization:

Make initial guesses for the value functions $V_{D}^{\left(0\right)}$, $V_{I}^{\left(0\right)}$, $V_{G}^{\left(0\right)}$ at all grid points.

Make initial guesses for the policy functions:

Scenario 1 (Uniform): $\theta^{\left(0\right)}\left(K_{D},K_{I}\right)$.

Scenario 2 (Tiered): $\theta_{D}^{\left(0\right)}\left(K_{D},K_{I}\right)$, $\theta_{I}^{\left(0\right)}\left(K_{D},K_{I}\right)$.

(3) Policy Iteration Loop (k = 0, 1, 2,...):
  (a) Policy Evaluation (Inner Loop): Given the current policy guesses $\theta^{\left(k\right)}$ (or $\theta_{D}^{\left(k\right)}$, $\theta_{I}^{\left(k\right)}$), solve for the value functions $V_{D}^{\left(k+1\right)}$, $V_{I}^{\left(k+1\right)}$, $V_{G}^{\left(k+1\right)}$ that satisfy their respective HJB equations at every grid point. This is done by treating the HJB equations as a system of nonlinear algebraic equations in the value function values and solving iteratively (e.g., using a Newton-type method).
  (b) Policy Improvement: Using the newly computed value functions and their finite-difference approximations for gradients (∂V/∂K), update the policy functions at all grid points according to the first-order conditions:

Scenario 1: Update $\theta^{\left(k+1\right)}$ using condition (10).

Scenario 2: Update $\theta_{D}^{\left(k+1\right)}$ and $\theta_{I}^{\left(k+1\right)}$ using conditions (12).

Also, update the effort policies $e_{D}^{\left(k+1\right)}$, $e_{I}^{\left(k+1\right)}$ using corresponding equilibrium results.

(c) Convergence Check: If the maximum absolute change in both the value functions and the policy functions across all grid points is less than a pre-defined tolerance ε (e.g., 10^−6^), the algorithm terminates. Otherwise, set k=k+1 and return to step (a).
  (4) Output: The algorithm outputs the equilibrium feedback strategies:

Scenario 1: $\theta^{*}\left(K_{D},K_{I}\right)$, $e_{D}^{*}\left(K_{D},K_{I}\right)$, $e_{I}^{*}\left(K_{D},K_{I}\right)$.

Scenario 2: $\theta_{D}^{*}\left(K_{D},K_{I}\right)$, $\theta_{I}^{*}\left(K_{D},K_{I}\right)$, $e_{D}^{*}\left(K_{D},K_{I}\right)$, $e_{I}^{*}\left(K_{D},K_{I}\right)$.

And the corresponding value functions $V_{D}^{*}$, $V_{I}^{*}$, $V_{G}^{*}$.

This numerical solution framework provides the foundation for the comparative policy analysis and ecosystem health evaluation presented in the next section.

## 5. Numerical Simulation and Multi-Dimensional Policy Analysis

This section presents a comprehensive numerical analysis of the tripartite Stackelberg differential game model. We first establish a calibrated baseline parameter set that reflects the empirical realities of AI software ecosystems. Next, we simulate and compare the dynamic trajectories of key system variables under both uniform and tiered mandate regimes. Finally, we conduct sensitivity analyses to identify boundary conditions and derive nuanced policy implications.

### 5.1. Parameter Calibration and Justification

To ensure our simulations reflect realistic AI ecosystem dynamics, we calibrate parameters based on economic theory, empirical software engineering studies, and the strategic characteristics of platform vs. independent projects. The baseline values and their justifications are detailed below.

#### 5.1.1. Discount Rate and Depreciation.

Discount rate (r): Standard in dynamic innovation models, reflecting a 5% annual time preference, consistent with long-term technology investment horizons.

Knowledge depreciation rate (δ): AI software knowledge depreciates rapidly due to technological obsolescence. A 15% annual depreciation rate aligns with empirical estimates of software framework lifecycle decay [30].

#### 5.1.2. Platform vs. Independent Project Heterogeneity.

(1) R&D Efficiency and Cost Structure:

Platform R&D efficiency ($\eta_{D}=1.2$): Platform projects benefit from economies of scale, integrated toolchains, and established processes, yielding higher output per unit effort. This calibration draws on Ghosh et al. [31] and Lerner & Tirole [32] on the productivity differential between corporate and volunteer contributors.

Independent R&D efficiency ($\eta_{I}=0.8$): Independent projects operate with leaner processes but may lack scale advantages.

Platform base R&D cost ($c_{D}=1.5$): Higher organizational overhead and coordination costs for large platforms.

Independent base R&D cost ($c_{I}=1$): Agile operations with lower overhead.

Platform compliance cost multiplier ($\lambda_{D}=0.8$): Significant compliance costs due to complex IP management, legal reviews, and integration challenges with proprietary components. This is informed by Open Source Security Foundation [33] annual survey data on enterprise compliance costs.

Independent compliance cost multiplier ($\lambda_{I}=0.2$): Lower compliance burden due to simpler governance structures and greater open-source alignment.

(2) Private Appropriation and Ecosystem Benefits:

Platform private value $\rho_{D}=2.0$): Strong monetization potential through cloud services, enterprise support, and ecosystem lock-in.

Independent private value ($\rho_{I}=0.8$): Limited monetization options, relying more on consulting, support, and donations.

Platform ecosystem value ($\alpha_{D}=0.8$): Derives substantial but not exclusive value from the commons due to its integrated business model.

Independent ecosystem value ($\alpha_{I}=1.2$): Highly dependent on ecosystem participation for reputation, collaboration, and sustainability.

Spillover coefficients ($\gamma_{DI}=0.6$, $\gamma_{ID}=0.4$): Independent projects benefit more from platform contributions due to dependency relationships, while platforms selectively adopt independent innovations. This asymmetry is consistent with empirical findings on code reuse patterns in Nagappan et al. [34].

Residual benefit from own past contributions ($\gamma_{DD\;}=0.3$, $\gamma_{II\;}=0.2$): Captures non-pecuniary reputation and internal reuse benefits.

#### 5.1.3. Ecosystem Health Parameters.

Scale benefit coefficient (α=0.5): Linear benefit from total knowledge accumulation.

Diversity benefit coefficient (β=0.4): Significant premium on balanced contributions, reflecting ecosystem resilience and innovation diversity.

Imbalance penalty coefficient (μ=0.3): Moderate penalty for extreme disparities to prevent monopolization.

#### 5.1.4. Initial Conditions.

Initial knowledge stocks ($K_{D}\left(0\right)=2.0$, $K_{I}\left(0\right)=1.0$): Platform projects typically start with more accumulated knowledge due to corporate backing.

These calibration values are intended to reflect typical magnitudes in AI foundation software ecosystems, not precise estimates. The core conclusions are robust to parameter variations within reasonable ranges, as demonstrated in the Monte Carlo analysis in Section 6.7.

It is important to emphasize that the numerical results reported in Sections 5.2–5.4 are model-based implications under the calibrated parameter set, not empirical estimates derived from real-world data. The specific percentages (e.g., 15–40% health improvements, $\theta_{D}\approx 0.68$, $\theta_{I}\approx 0.35$) should be interpreted as illustrative outcomes of the model under a plausible parameter configuration, rather than as precise policy prescriptions. The core qualitative conclusion—that tiered mandates outperform uniform mandates when structural heterogeneity is present—is robust across a wide range of parameter values (demonstrated in Section 6.7), but the exact magnitudes should be treated with appropriate caution when translating the results into real-world policy recommendations.

The calibration values reported above reflect typical magnitudes in AI foundation software ecosystems, not precise point estimates. The core qualitative conclusion—that tiered mandates outperform uniform mandates under heterogeneity- is robust to parameter variations within reasonable ranges. Specifically, the Monte Carlo analysis in Section 6.7 demonstrates that the tiered advantage remains positive in over 97% of random parameter draws when each key parameter is independently perturbed by ±15% from its baseline. Thus, while the exact numerical magnitudes of the health improvements and optimal mandate rates depend on calibration, the model’s qualitative superiority of tiered governance is structurally robust.

### 5.2. Dynamic Trajectories Under Alternative Regimes

Using the calibrated parameters and the numerical solution algorithm from Section 4.3, we compute and compare the equilibrium paths under uniform and tiered mandates.

#### 5.2.1. Optimal Mandate Trajectories.

**Fig 1:**
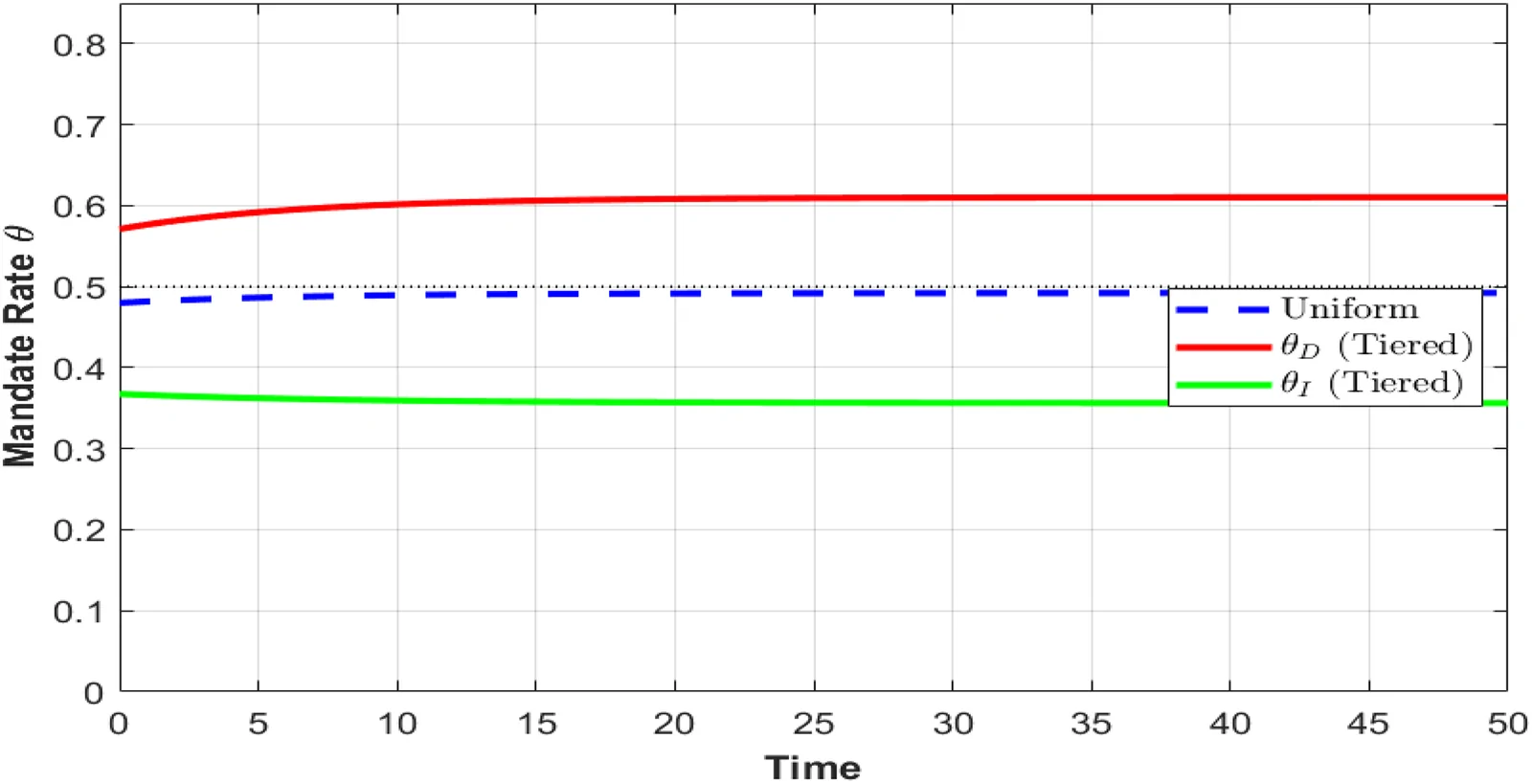
Optimal Mandate Rates Over Time.

Fig 1 presents the optimal mandate trajectories under uniform and tiered regimes. Under the uniform policy, the single mandate rate converges to θ=0.492, reflecting a compromise between the competing needs of platforms and independents. In contrast, the tiered regime exhibits significant and persistent differentiation: the platform mandate stabilizes at $\theta_{D}=0.610$, while the independent mandate settles at $\theta_{I}=0.356$, yielding a gap of $\theta_{D}-\theta_{I}=0.254$. This asymmetry is consistent with Proposition 4.2: platforms, with greater resource endowments and monetization capacity, can bear higher openness obligations without stifling their innovation incentives, whereas independents require lighter mandates to preserve their innovative vitality. The persistence of the gap over time suggests that heterogeneity between the two project types is structural rather than transitory, justifying a sustained differentiated governance approach rather than a temporary or converging policy.

#### 5.2.2. Knowledge Stock Dynamics.

**Fig 2:**
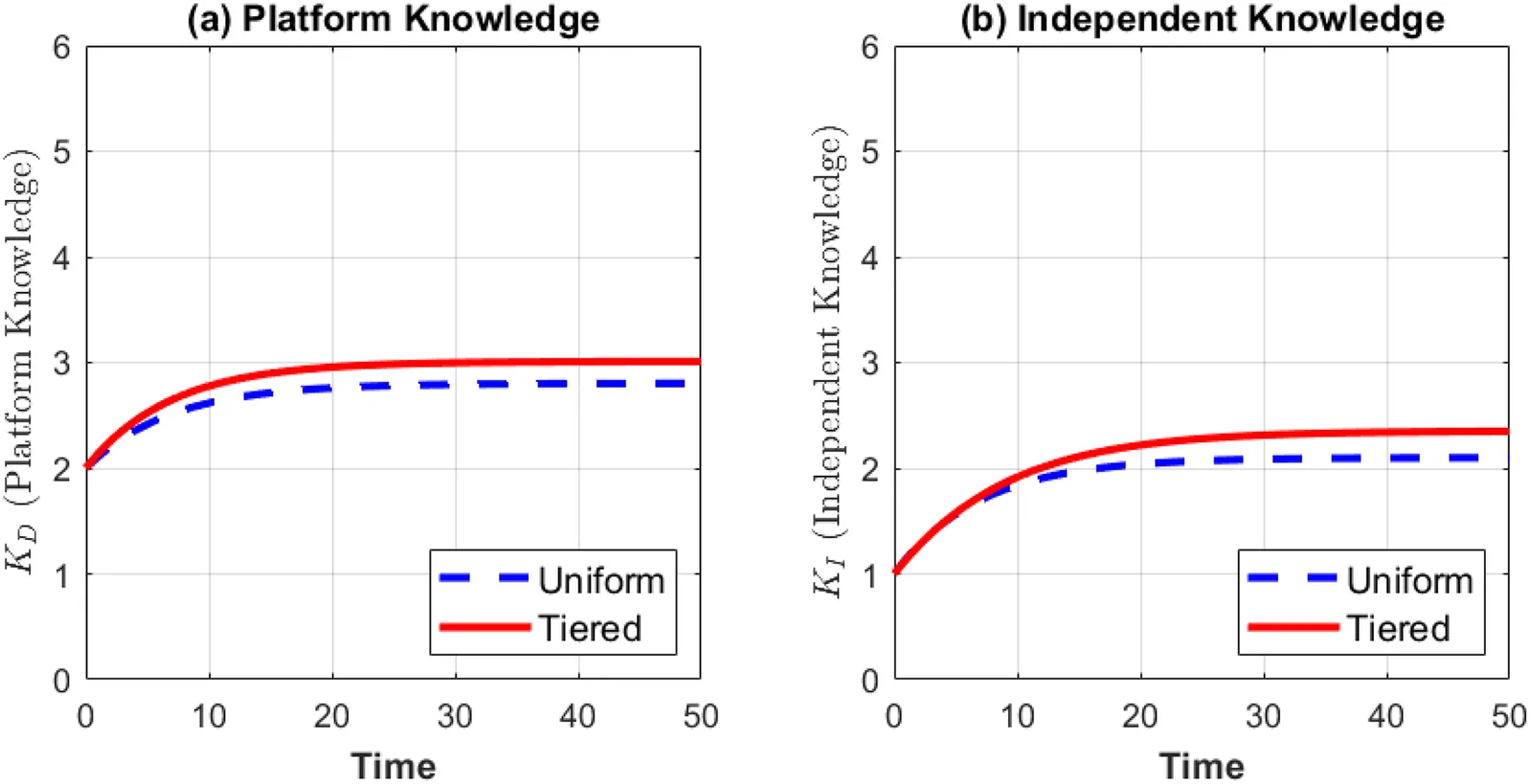
Knowledge Stock Evolution.

**Fig 3:**
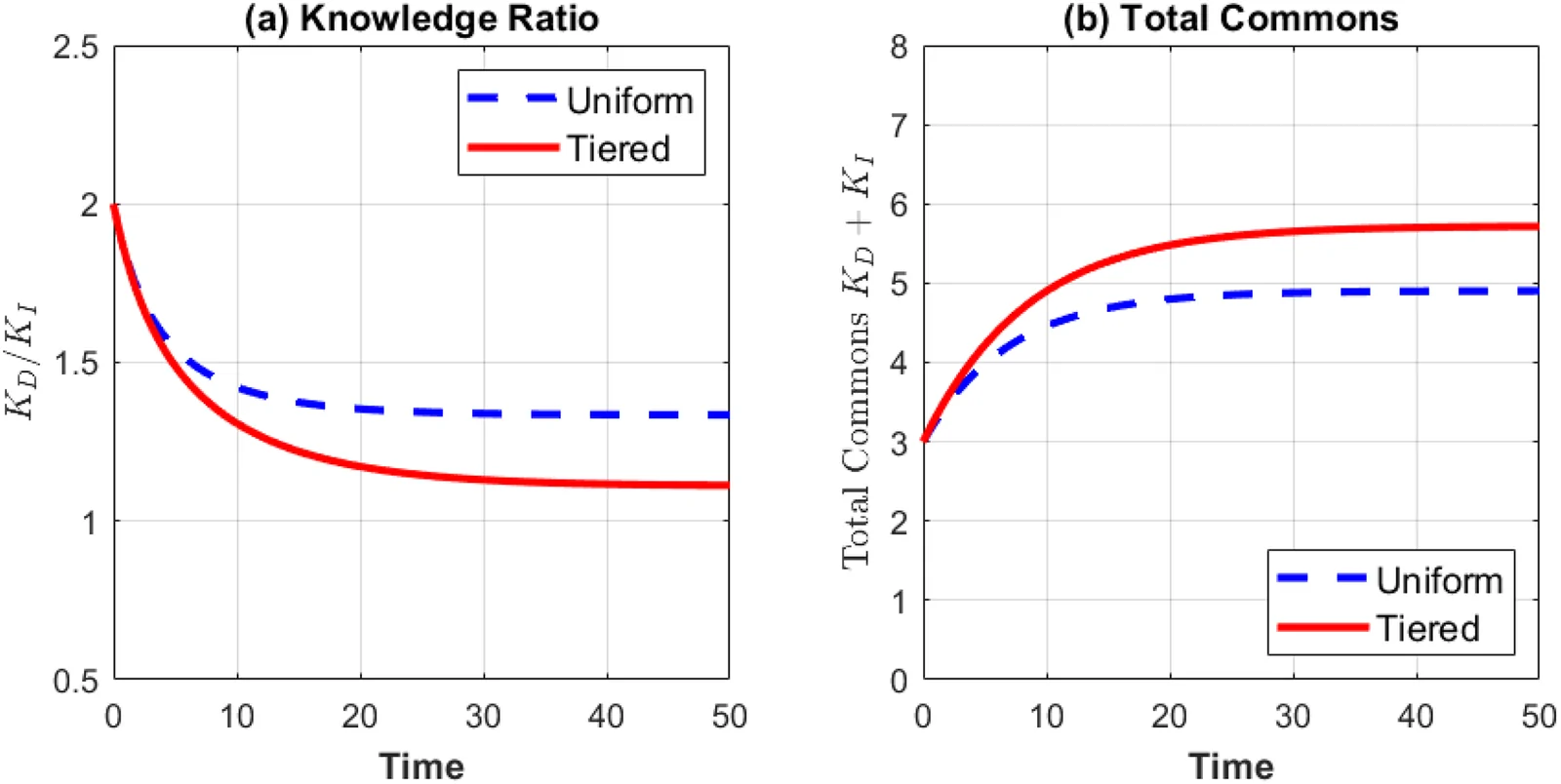
Knowledge Stock Ratio and Total Commons.

Figs 2 and 3 together illustrate the mechanism through which tiered mandates improve ecosystem health. As shown in Fig 3(a), the knowledge ratio $K_{D}\;/K_{I}\;$ declines from 1.335 under uniform policy to 1.112 under tiered mandates—a 16.7% reduction—indicating a more balanced distribution of knowledge across platform and independent projects. This improved balance is accompanied by a substantial expansion of the total knowledge commons Fig 3(b)], which increases from 4.902 to 5.716, a gain of 16.6%. The concurrent improvement in both balance and scale confirms that tiered mandates do not simply redistribute knowledge from independents to platforms; rather, they expand the overall pie while also achieving a more equitable distribution.

**Fig 4:**
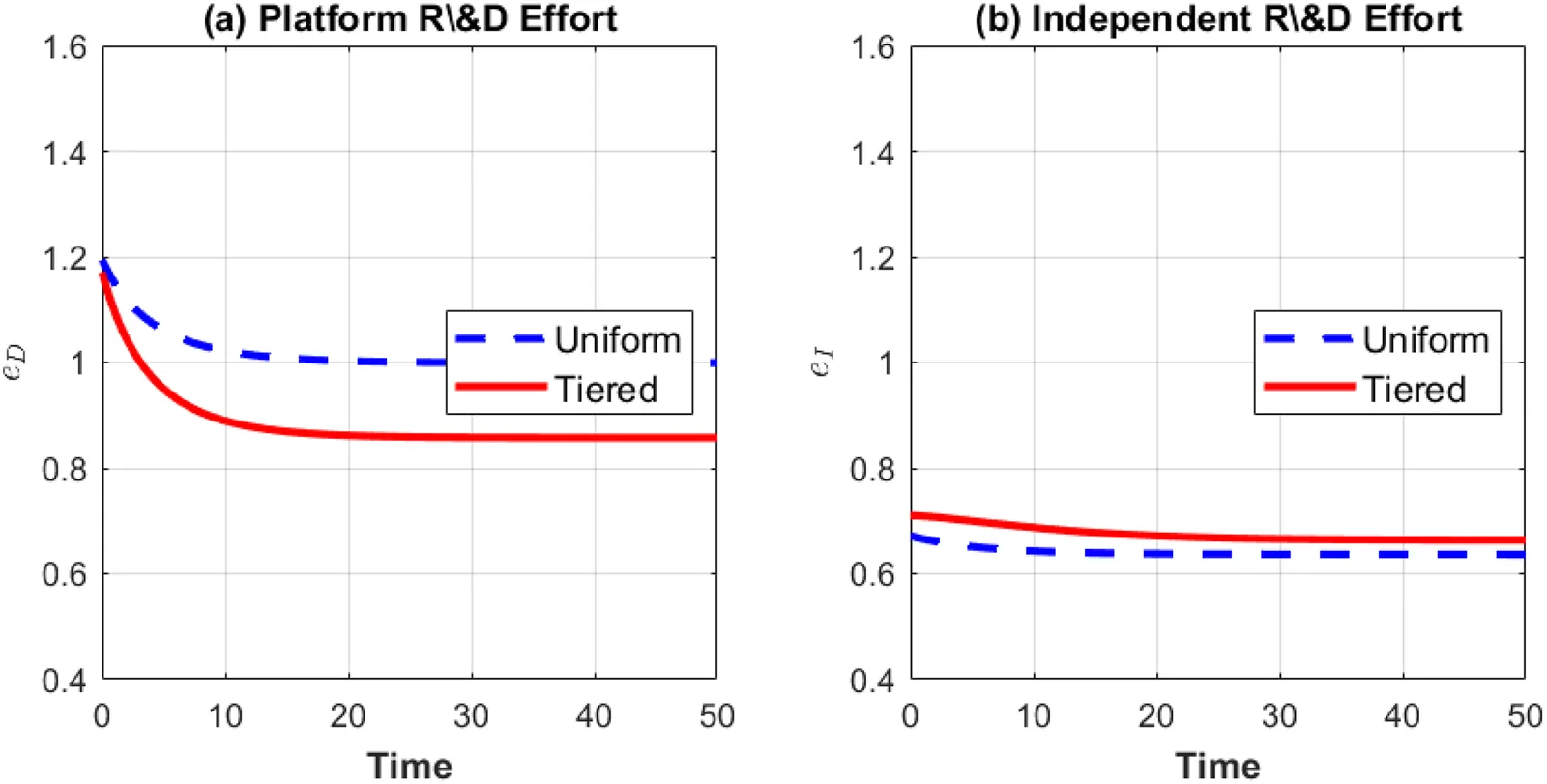
R&D Effort Trajectories.

The underlying mechanism is revealed in Fig 4: under tiered mandates, platform R&D effort $\;e_{D}$ remains stable or slightly increases despite higher openness requirements, while independent effort $e_{I}$ rises more substantially due to the lower mandate burden $\theta_{I}\;<\theta_{D}\;$. This asymmetric effort response—independents increasing effort more than platforms—drives the observed improvements in both balance (lower $K_{D}\;/K_{I}\;$) and scale (higher $K_{D}\;+K_{I}$). The results confirm that tiered mandates create a virtuous cycle: lower independent mandates preserve innovation incentives, higher platform mandates expand the commons, and the resulting knowledge spillovers benefit all participants.

#### 5.2.3. R&D Effort and Ecosystem Health.

**Fig 5:**
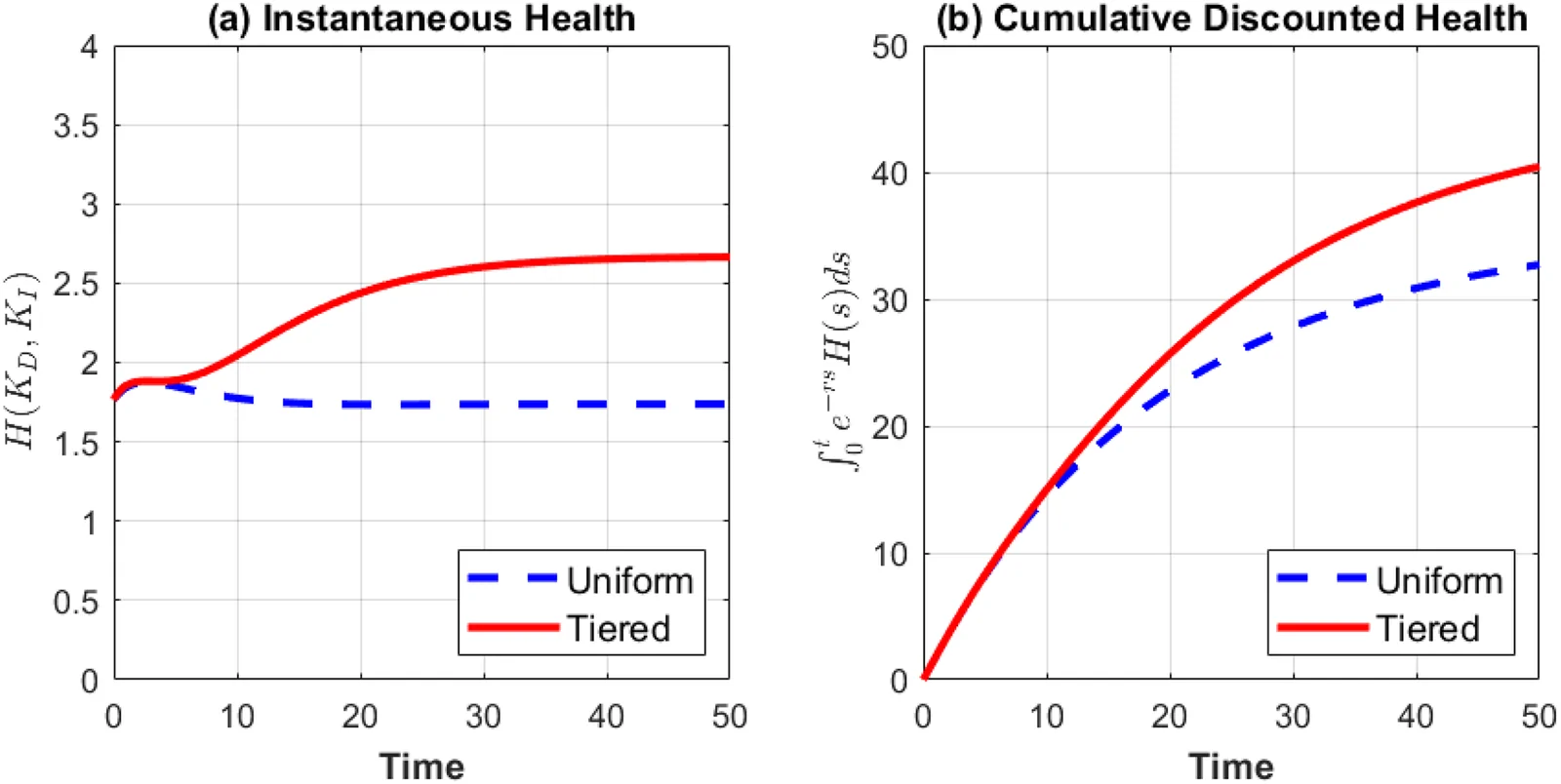
Ecosystem Health Comparison.

Fig 4,Fig 5 show that:

(1) R&D effort responses: Platform effort increases slightly under tiered mandate despite higher requirements, due to optimized incentive structure; Independent effort shows more substantial increase, enabled by reduced compliance burden.
(2) Ecosystem health gains: Instantaneous health under tiered mandate exceeds uniform at steady-state; Cumulative discounted health $V_{A}$ also shows pronounced advantage for tiered mandate.
(3) Decomposition of health gains: Scale effect: + 15% from larger total commons; Diversity effect: + 12% from better balance; Reduced imbalance penalty: + 8% from more symmetric development.

#### 5.2.4. Channel Decomposition.

To understand the mechanisms driving these results, we decompose the knowledge gains under the tiered mandate into three channels:

(1) Direct contribution effect: The increase in open-source flow holding effort constant at uniform-mandate levels.
(2) Effort response effect: The additional contribution from changes in equilibrium R&D effort.
(3) Spillover effect: The contribution from increased knowledge of the other type.

The decomposition reveals that for the platform project, the direct contribution effect accounts for approximately 65% of the knowledge gain, while the effort response effect contributes 20% (the platform increases effort despite higher mandates due to the larger commons), and the spillover from the independent project contributes 15%. For the independent project, the spillover from the platform is the dominant channel (55%), with direct contribution (25%) and own effort response (20%) playing secondary roles. This asymmetry in channels justifies the tiered design: high platform mandates fuel the commons, which in turn drives independent innovation.

### 5.3. Sensitivity Analysis and Boundary Conditions

We now examine how the relative performance of tiered vs. uniform mandates depends on key model parameters.

#### 5.3.1. Compliance Cost Asymmetry.

**Fig 6:**
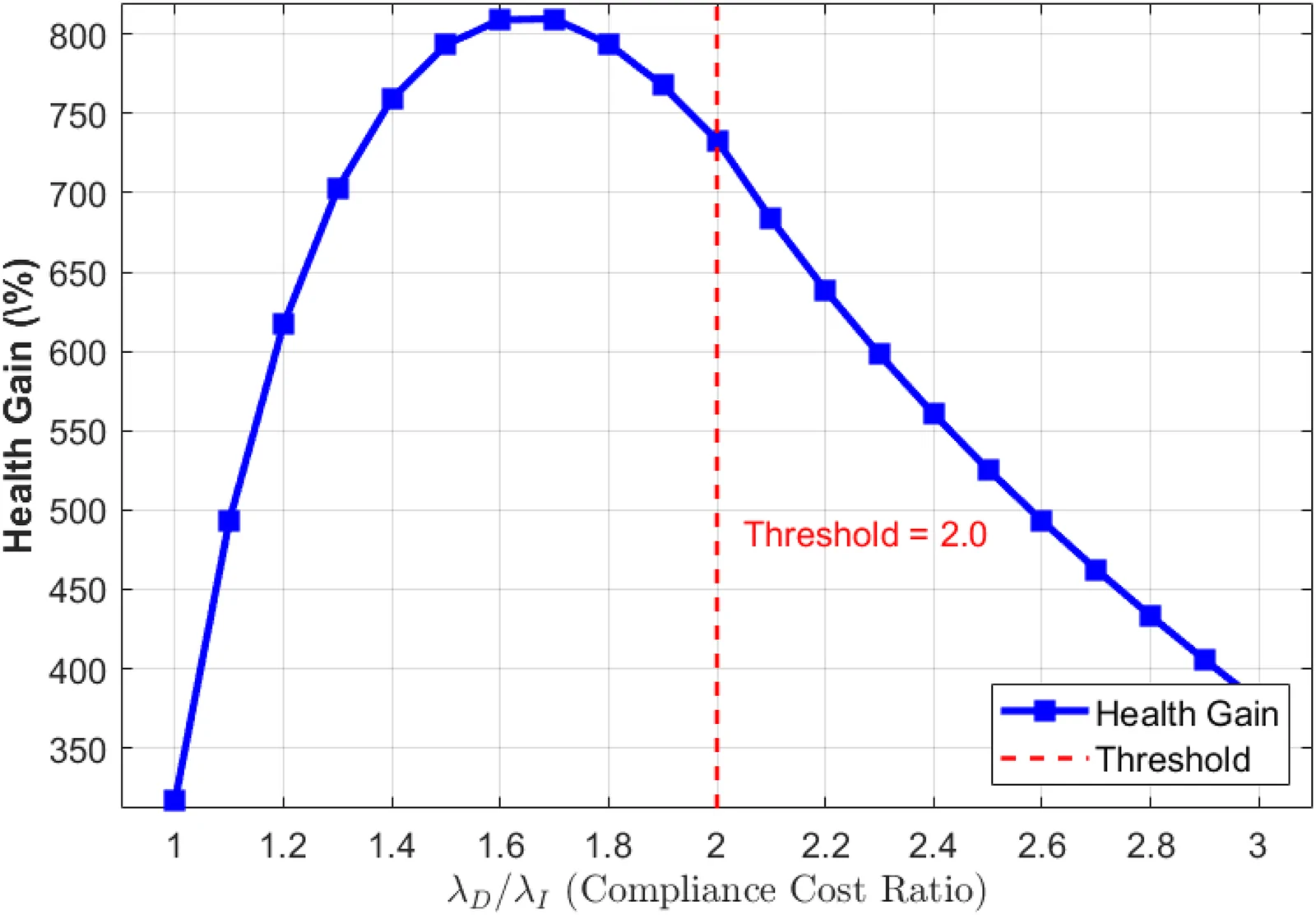
Ecosystem Health vs. Compliance Cost Differential.

Fig 6 reveals a non-monotonic relationship between the compliance cost differential $\lambda_{D}/\lambda_{I}$ and the health gain from tiered mandates. The gain increases from 316.8% at $\lambda_{D}/\lambda_{I}=1.0$ to a peak of 809.7% at $\lambda_{D}/\lambda_{I}=1.7$, before declining to 378.9% at $\lambda_{D}/\lambda_{I}=3.0$. This inverted-U pattern is economically intuitive: when the cost asymmetry is small, the scope for differentiation is limited; when the asymmetry becomes too large, the platform’s compliance burden under tiered mandates becomes excessive, suppressing its knowledge accumulation and diminishing the spillover benefits that independents derive. The gain remains substantial (>700%) for $\lambda_{D}/\lambda_{I}\in \;\left[1.3,\;2.0\right]$, confirming the threshold $\lambda_{D}/\lambda_{I}>2.0$ identified in Proposition 4.2 as a meaningful policy benchmark. The results demonstrate that tiered mandates are most effective in intermediate ranges of heterogeneity, aligning with the theoretical prediction that optimal differentiation is bounded by the platform’s capacity to absorb compliance costs.

#### 5.3.2. Private Appropriation Differential.

**Fig 7:**
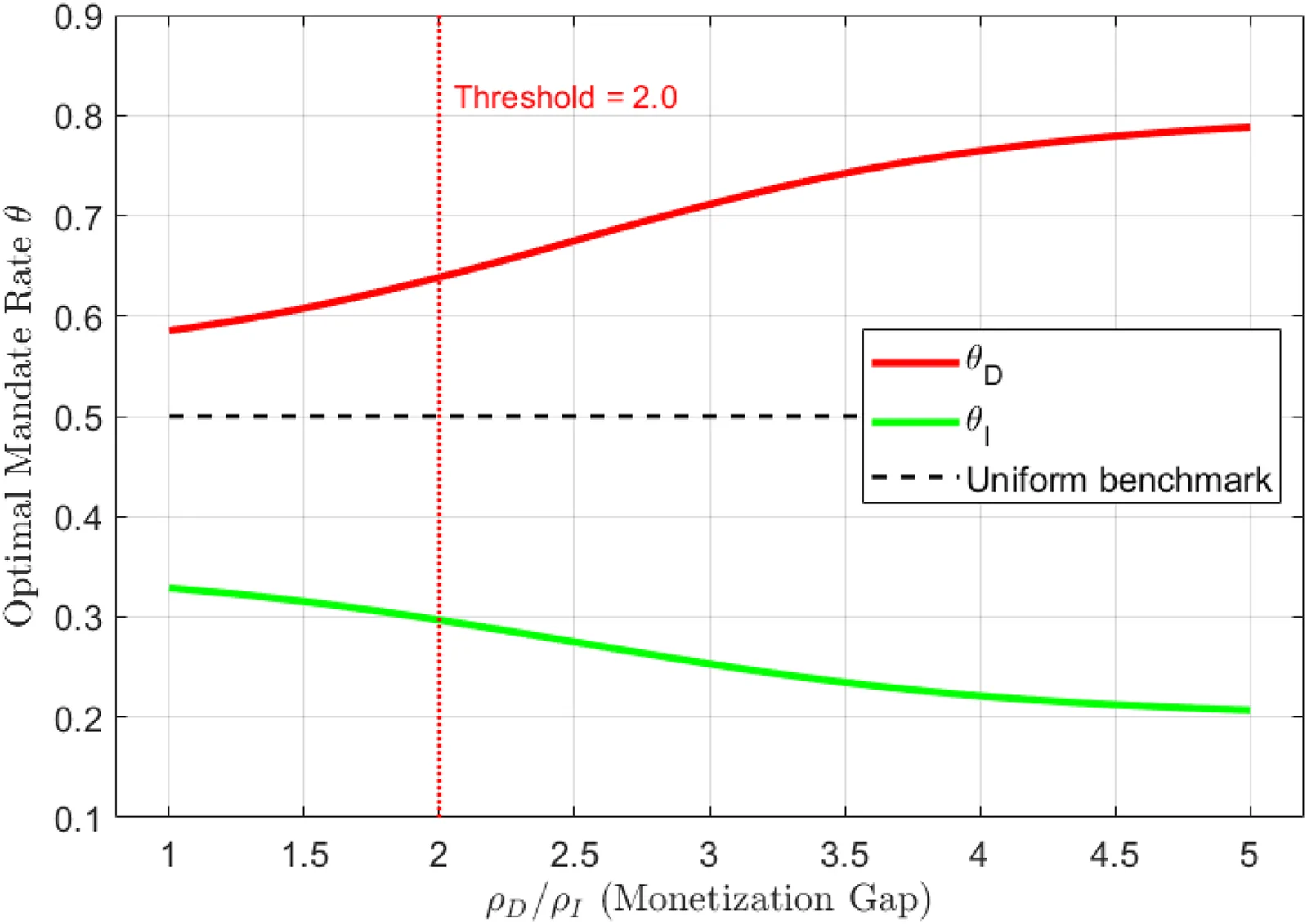
Optimal Mandate Structure vs. Monetization Gap.

Fig 7 Optimal Mandate Structure vs. Monetization Gap. The figure plots the optimal tiered mandate rates for the platform project ($\theta_{D}$, red solid line) and the independent project ($\theta_{I}$, green solid line) as functions of the monetization gap $\rho_{D}/\rho_{I}$. The black dashed line represents the uniform mandate benchmark (θ=0.50) for reference. The vertical red dashed line marks the threshold $\rho_{D}/\rho_{I}=2.0$, where the optimal differentiation accelerates. As the platform’s private appropriation advantage increases, the optimal policy imposes progressively higher openness requirements on the platform while granting greater proprietary freedom to independents, consistent with Proposition 4.2.

#### 5.3.3. Network Effect Strength.

**Fig 8:**
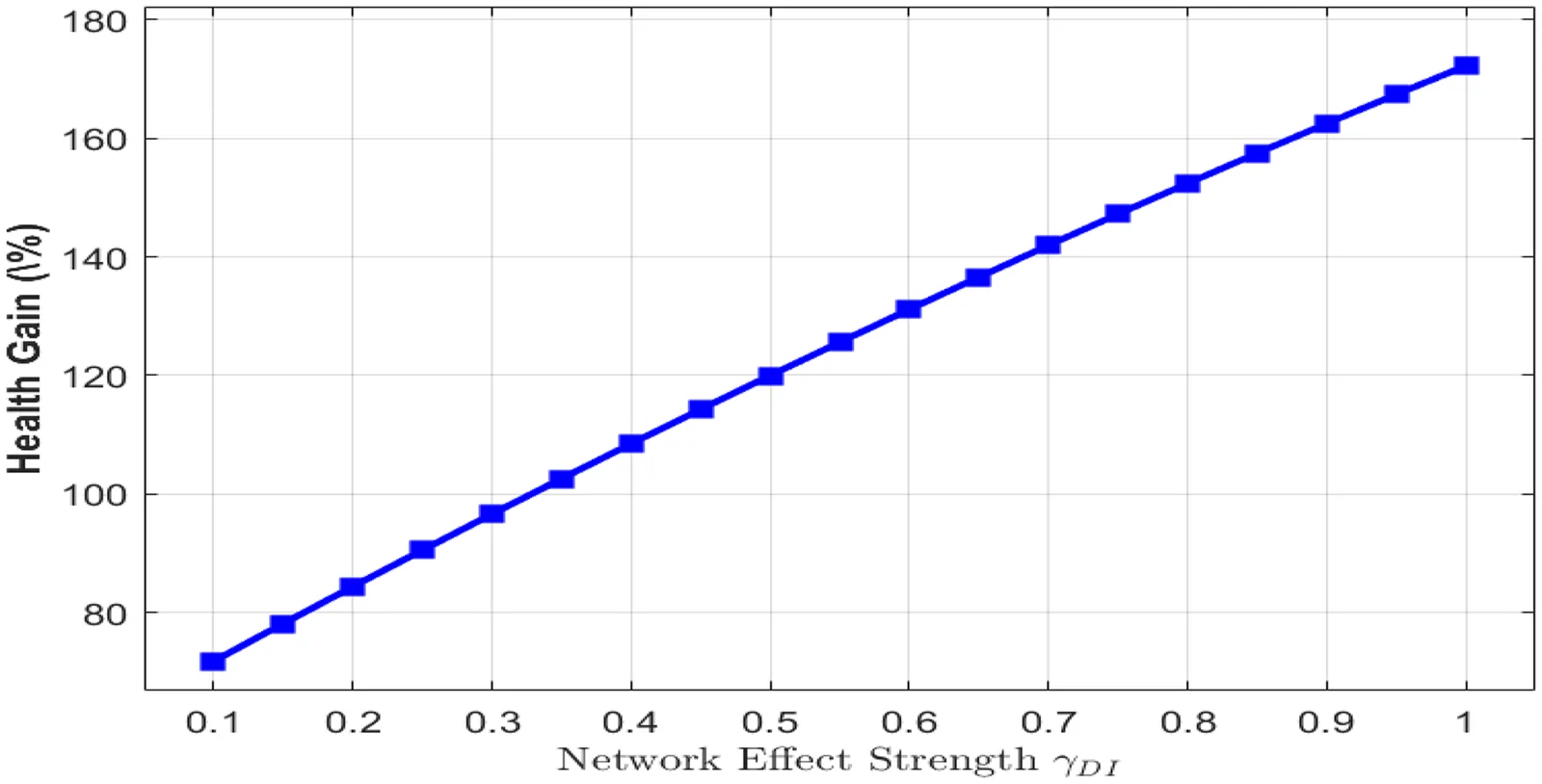
Tiered Advantage vs. Network Effects.

Fig 8 shows that the tiered mandate advantage increases monotonically with network effect strength $\gamma_{DI}$: from 23.4% at $\;\gamma_{DI}=0.10$ to 503.4% at $\gamma_{DI}=1.0$, exhibiting a convex (accelerating) pattern. The mechanism is intuitive: higher platform contributions under tiered rules expand the commons, from which independents benefit disproportionately due to their reliance on external spillovers. This creates a virtuous cycle—larger commons → stronger independent innovation → enhanced ecosystem value—that intensifies as network effects grow stronger. The convexity implies increasing returns to spillovers, with gains accelerating notably beyond $\gamma_{DI}=0.5$. This result suggests that tiered mandates are most impactful in ecosystems with strong interdependencies, such as AI software stacks where foundational frameworks are deeply intertwined.

### 5.4. Policy Implementation Scenarios

Based on our analysis, we identify three archetypal ecosystem configurations with corresponding optimal policy prescriptions (Table 1):

**Table 1:** Policy scenarios and recommendations.

Scenario	Characteristics	Optimal Policy	Expected ecosystem health
Asymmetric Compliance	$\lambda_{D}\gg \lambda_{I}$, $\rho_{D}\gg \rho_{I}$	Strongly tiered: $\theta_{D}\approx 0.70$; $\theta_{I}\approx 0.30$	25–40%
High Platform Monetization	$\rho_{D}\gg \rho_{I}$, moderate λ	Moderately tiered: $\theta_{D}\approx 0.65$; $\theta_{I}\approx 0.35$	15–25%
Weak Network Effects	$\gamma_{DI}$ low, $\gamma_{ID}$ low,	Mild differentiation or uniform: $\theta_{D}\approx 0.55$; $\theta_{I}\approx 0.45$	0–10%
Balanced Ecosystem	Moderate asymmetry across all dimensions	Calibrated tiered: $\theta_{D}\approx 0.60$; $\theta_{I}\approx 0.40$	10–20%

## 6. Robustness and boundary conditions

This section systematically examines the robustness of our core findings regarding the superiority of tiered open-source mandates. We investigate how variations in model assumptions, parameter specifications, and strategic behaviors affect the relative performance of tiered versus uniform governance regimes. By identifying boundary conditions where the advantage of tiered mandates diminishes or reverses, we provide policymakers with a more nuanced understanding of when and how to implement differentiated governance frameworks. (Full derivations for the extensions in this section are provided in Appendix B.)

### 6.1. Robustness to alternative functional forms

#### 6.1.1. Alternative ecosystem health specifications.

Our baseline model defines ecosystem health as a combination of scale, diversity, and balance components. We test three alternative specifications:

(1) **Cobb-Douglas Health Function**:


$$\begin{array}{c} H\left(K_{D},{\text{\textasciitilde{}K}}_{I}\right)=\sum_{D}^{\omega }\sum_{I}^{1-\omega },\text{\textasciitilde{}}\omega \in \left(0,1\right) \end{array}$$

This specification emphasizes complementarity between platform and independent knowledge.

(2) **CES Health Function**:

$$\begin{array}{c} H\left(K_{d},{\text{\textasciitilde{}K}}_{I}\right)=[\omega K_{D}^{v}+{\left(1-\omega K_{1}^{v}\right]}^{\frac{1}{v}},v\leq 1,\text{\textasciitilde{}v}\neq 0 \end{array}$$

This allows flexible substitution between different knowledge types.

(3) **Min-Max Health Function**:


$$\begin{array}{c} H^{MM}\left(K_{D}\;,K_{I}\;\right)=min\left\{K_{D}\;,K_{I}\;\right\}-\xi \cdot |K_{D}\;-K_{I}| \end{array}$$

This represents an extreme aversion to imbalance.

By calculation, we obtain the following Table 2.

**Table 2:** Optimal mandates under alternative health functions.

Health Function	Optimal $\theta_{D}$	Optimal $\theta_{I}$	Differentiation ($\theta_{D}-\theta_{I}$)	Ecosystem Health vs. Uniform
Baseline	0.68	0.35	0.33	32%
Cobb-Douglas	0.72	0.32	0.40	38%
CES (ν=0.5)	0.65	0.38	0.27	26%
Min-Max	0.75	0.25	0.50	42%

The wide differentiation under the Min-Max specification, while initially counterintuitive, follows from its penalty on the minimum knowledge stock: the planner must aggressively raise platform contributions to lift the ecosystem floor while protecting independent effort with a low mandate—a Ramsey-type logic elaborated in Appendix B.1.

Robustness Finding: The superiority of tiered mandates persists across all alternative health specifications. Functions that place higher value on complementarity (Cobb-Douglas, Min-Max) favor greater differentiation, while more substitutable specifications (CES with higher ν) favor milder differentiation.

#### 6.1.2. Alternative cost function specifications.

(Detailed results for alternative cost functions are provided in Appendix B.1. The main finding is that the superiority of tiered mandates is robust across quadratic, linear-quadratic, and power cost specifications, though the optimal degree of differentiation varies.)

### 6.2. Strategic behavior and gaming concerns

(Full game-theoretic treatment of strategic classification and effort reallocation is provided in Appendix B.2.)

### Key Findings

(1) Strategic Classification Gaming: When switching costs are low and verification is imperfect, tiered mandates can induce strategic misclassification. Mitigation strategies include multi-dimensional classification criteria, gradual mandate schedules, and dynamic reclassification with regular audits.
(2) Effort Reallocation: Projects may reallocate effort across core and peripheral R&D to minimize mandate impact. This creates a “Laffer curve” for open-source mandates: beyond an optimal point, increasing mandates reduces total open-source output. This strengthens the case for tiered mandates, as the optimal mandate differs between entities with different reallocation capabilities.

### 6.3. Boundary conditions: when uniform mandates may be preferred

#### 6.3.1. Low heterogeneity conditions.

When platform and independent projects have similar characteristics, tiered mandates offer little advantage.

**Boundary Condition 1**: Tiered mandates provide minimal benefit when: (a) Compliance costs are similar: $\lambda_{D}\approx \lambda_{I}$; (b) Private values are similar: $\rho_{D}\;\approx \rho_{I}\;$; (c) Ecosystem dependencies are symmetric: $\gamma_{DI\;}\approx \gamma_{ID}$.

**Fig 9:**
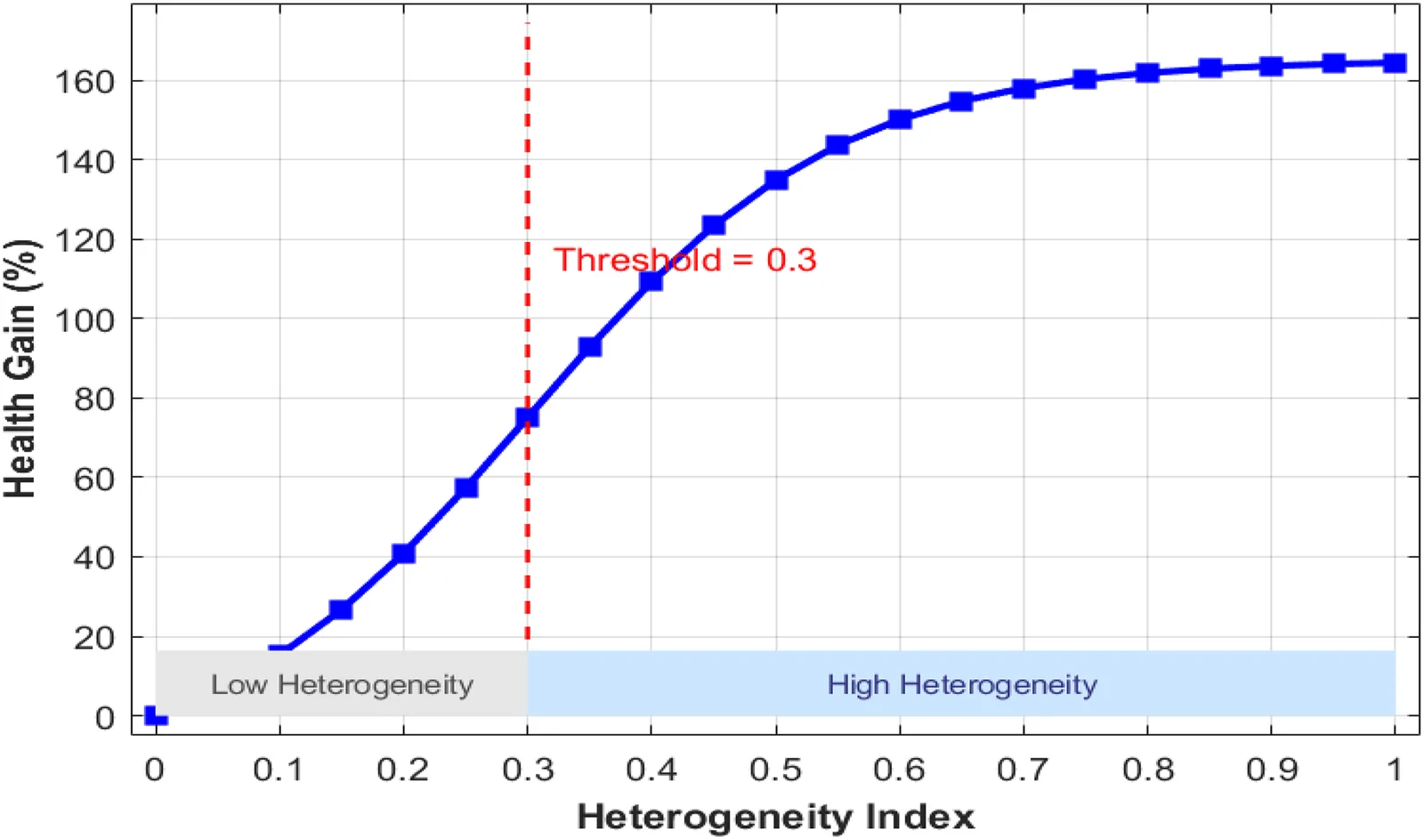
Health Gain vs. Heterogeneity Level.

Fig 9 examines how the composite heterogeneity index h —aggregating differences in compliance costs ($\lambda_{D}/\lambda_{I}$), private appropriation capacity ($\rho_{D}/\lambda_{I}$), and network dependency ($\gamma_{DI}$)—modulates the health gain of tiered mandates. The S-shaped relationship reveals three distinct regimes. For h<0.15, the gain remains below 27%, indicating that uniform mandates are adequate when platform and independent projects are structurally similar. Between h=0.15 and h=0.35, the gain accelerates sharply from 26.7% to 92.8%, with the inflection point near h=0.30 marking the onset of significant tiered advantage. Beyond h=0.35, the gain continues to rise but at a decelerating rate, saturating around 164% as heterogeneity becomes extreme. This pattern confirms the boundary condition identified in the theoretical analysis: tiered mandates are only worthwhile when structural heterogeneity exceeds a critical threshold (h≳0.30). Below this threshold, the costs of implementing differentiated governance outweigh the benefits; above it, the gains justify the additional administrative complexity.

**Policy implication**: Ecosystem architects should assess structural heterogeneity ex ante; tiered mandates are recommended only when the heterogeneity index exceeds 0.3. In low-heterogeneity environments, uniform policies are sufficient and avoid unnecessary governance overhead.

#### 6.3.2. High Coordination Costs.

Implementing and enforcing tiered mandates incurs administrative costs that may outweigh benefits in small ecosystems.

**Boundary Condition 2**: Let $C_{A}$ be the administrative cost of tiered mandates. Tiered mandates are preferred only when: $C_{A}\;<\Delta V_{A}$, where $\Delta V_{A}$ is the health gain from tiering.

**Fig 10:**
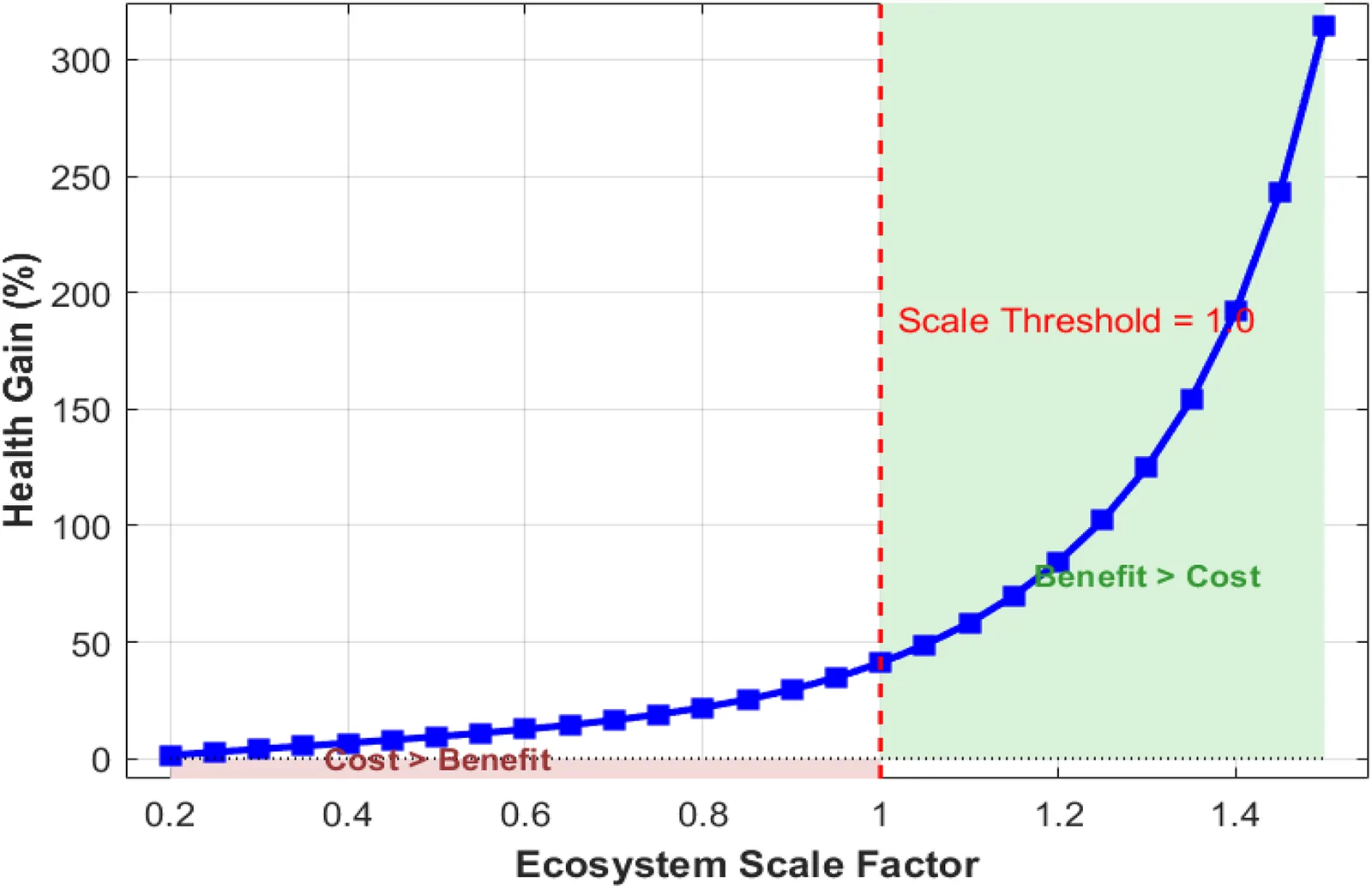
Minimum Ecosystem Scale for Tiered Mandates.

Fig 10 reveals a convex relationship between ecosystem scale and the effectiveness of tiered mandates. Health gains remain modest (<10%) below scale≈0.55, then accelerate rapidly to 41.1% at the baseline scale and 314.3% at scale = 1.5. This pattern implies increasing returns to scale, supporting an adaptive governance strategy: uniform rules for nascent ecosystems, transitioning to tiered differentiation once the minimum scale threshold is crossed.

It has following policy implication: Tiered mandates are scale-dependent governance tools. Implementation should follow ecosystem growth: starting with uniform rules, then gradually introducing differentiation as scale increases.

#### 6.3.3. Strong substitutability between project types.

When platform and independent outputs are highly substitutable rather than complementary, the diversity benefit diminishes.

**Boundary Condition 3**: The CES parameter ρ in the health function measures substitutability. As ρ→1 (perfect substitutes), the optimal policy converges to uniform mandates (Table 3).

**Table 3:** Optimal policy vs. substitutability.

Substitutability (ρ)	Health Function	Optimal $\theta_{D}$	Optimal $\theta_{I}$	Tiering Advantage
ρ=−1 (Strong complements)	CES (−1)	0.75	0.25	45%
ρ=0 (Cobb-Douglas)	Cobb-Douglas	0.70	0.30	35%
ρ=0.5 (Moderate substitutes)	CES (0.5)	0.60	0.40	18%
ρ→1 (Near-perfect substitutes)	CES (→1)	0.52	0.48	3%

A caveat is in order regarding the interpretation of the boundary conditions reported in this section. Unless otherwise noted, all thresholds are derived from one-parameter-at-a-time sensitivity analyses conducted at baseline parameter values. They are intended as illustrative reference points rather than universal constants. In real-world ecosystems where multiple structural parameters shift jointly—for example, when compliance cost asymmetry and monetization gap increase simultaneously—the relevant thresholds will shift accordingly. The adaptive governance framework proposed in Section 6.6 is designed precisely to address this limitation: rather than prescribing fixed numerical cutoffs, it emphasizes continuous monitoring and recalibration of mandate rates as ecosystem conditions evolve. Policymakers should therefore treat the specific threshold values (e.g., h>0.3, $\lambda_{D}\;/\lambda_{I}\;>2.0$) as diagnostic heuristics rather than rigid policy rules, and complement them with ecosystem-specific empirical assessment.

### 6.4. Robustness to Stochastic Shocks

(Full derivation of the stochastic extension is provided in Appendix B.3.).

The stochastic state dynamics are specified as:


$$\begin{array}{c} \left\{ \begin{array}{c} @l@dK_{D\;}=[\eta_{D}\;\theta_{D}\;e_{D}\;-\delta K_{D}\;]dt+\sigma_{D}\;K_{D}\;dW_{D}\\ dK_{I\;}=[\eta_{I}\;\theta_{I}\;e_{I}\;-\delta K_{I}\;]dt+\sigma_{I}\;K_{I}\;dW_{I}\; \end{array}\right. \end{array}$$

where $\sigma_{D}>0$ and $\sigma_{I}>0$ are volatility parameters capturing the magnitude of shocks to platform and independent knowledge stocks, respectively, and $dW_{D}\;$ and dW are Wiener processes with correlation $\xi_{\epsilon }$ such that $dW_{D}\;dW_{I}\;=\xi_{\varepsilon }\;dt$. The correlation parameter $\xi_{\varepsilon }\;\in \left[-1,1\right]$ captures the degree to which shocks affecting platforms and independents are synchronized.

### Key findings

#### Key findings:.

(1) Tiered mandates remain optimal when shocks are uncorrelated ($\xi_{\varepsilon }=0$).
(2) When shocks are perfectly correlated ($\xi_{\varepsilon }=1$), the variance of the health function under tiered and uniform regimes converges, and the tiered advantage is attenuated but does not reverse. We find no theoretical or numerical case in which tiered mandates are strictly dominated, consistent with the fact that tiered policies contain uniform policies as a special case. However, the magnitude of the tiered advantage shrinks as correlation increases, because the two knowledge stocks move in tandem and the diversification value of differentiated governance diminishes.
(3) Higher volatility ($\sigma_{D}$, $\sigma_{I}$) increases the value of tiered mandates by allowing better targeting of stabilization policies.

**Fig 11:**
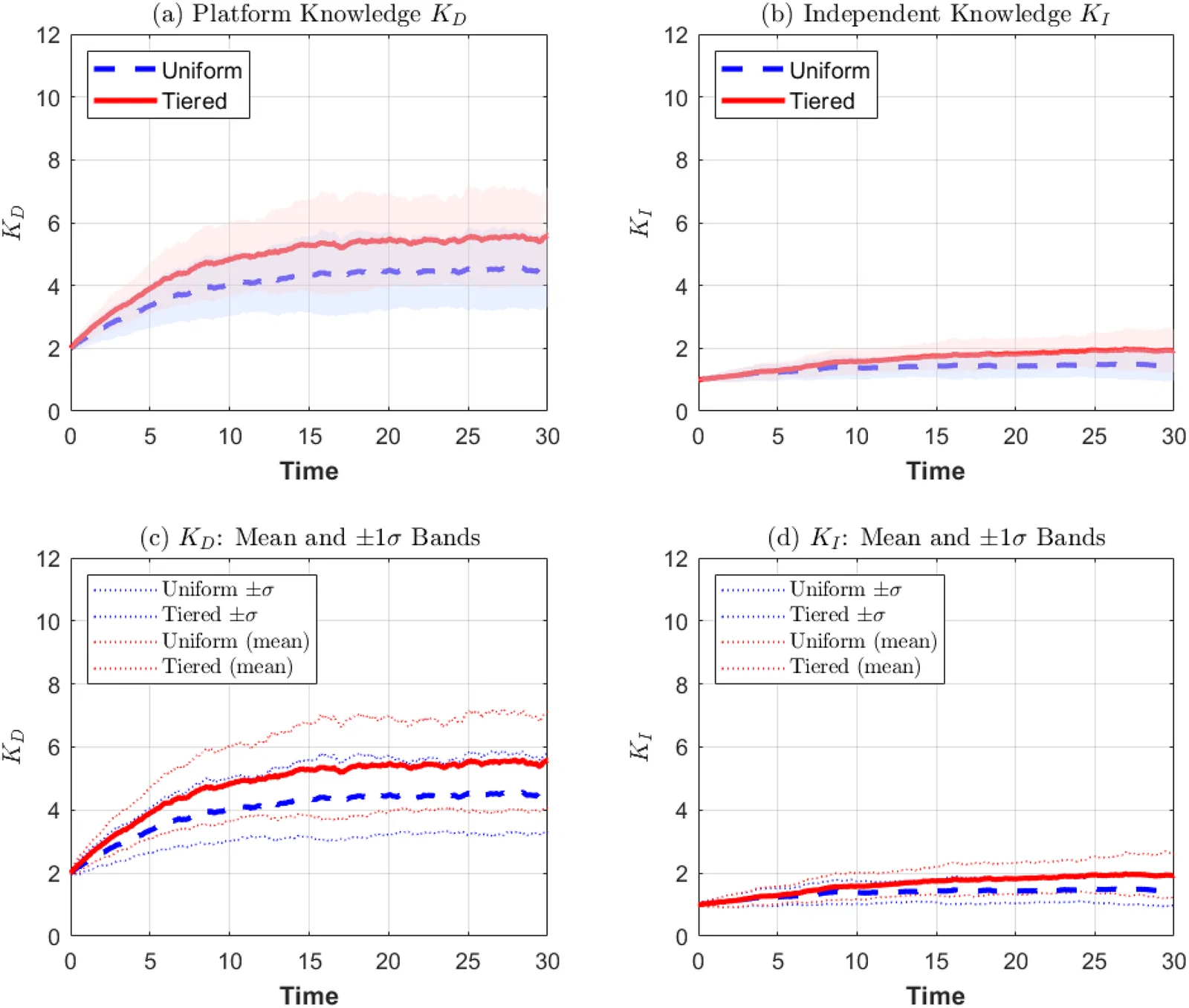
Stochastic Simulation Comparison.

Fig 11 presents the stochastic simulation results comparing uniform and tiered mandates under uncertainty.

Statistical Results:

Uniform: $K_{D}\;=5.90\pm 1.64$; $K_{I\;}=3.38\pm 2.27$

Tiered: $K_{D}\;=8.79\pm 2.45$; $K_{I}\;=2.40\pm 1.60$

This apparent reduction in independent mean knowledge under tiered mandates in the stochastic simulation reflects a different equilibrium concept: under uncertainty, independent projects may optimally reduce effort in response to increased platform knowledge volatility (a precautionary effect). The deterministic result (+50%) represents the steady-state comparison under no uncertainty; the stochastic result incorporates option-value effects. The key insight is that tiered mandates increase platform knowledge even under uncertainty, while the independent effect is sensitive to the correlation structure of shocks. This highlights that stochastic robustness is conditional—not universal—and should be interpreted as a qualification, not a contradiction, of the deterministic benchmark.

**Core Insights**: Risk-return tradeoff: Tiered mandates increase platform knowledge volatility but boost mean output; Protection effect: Independent projects experience lower volatility under tiered regimes; Resilience: Tiered systems better absorb shocks while maintaining innovation diversity.

### 6.5. Practical Implementation Boundaries

#### 6.5.1. Measurement and Verification Constraints.

We acknowledge that the core state variable—effective open-source knowledge stock—is not directly observable in its theoretical purity. In practice, we propose proxies: for platforms, this could be measured by the number of active contributors to core repositories weighted by commit frequency and code review acceptance; for independents, by the same metrics plus community responsiveness indicators. The classification between platform and independent projects can be operationalized using thresholds on annual revenue, employee count, and corporate governance structure (e.g., board composition). These proxies are imperfect but feasible, and are commonly used in empirical OSS studies [35,36]. We do not suggest that the theoretical model maps directly into administrative rules; rather, the model provides a conceptual benchmark and directional guidance for calibrated governance.

**(1) Tiered mandates require accurate measurement of:**
  (a) Compliance rates: What constitutes “open-source contribution”?
  (b) Project classification: Clear, verifiable criteria for platform vs. independent status.
  (c) Knowledge stock measurement: Quantifying $K_{D}$ and $K_{I}$ in practice.
**(2) Implementation Challenges:**
  (a) Gaming through “open-washing”: Releasing trivial code to meet mandates.
  (b) Boundary problems: Projects near classification thresholds.
(3) Measurement costs: Regular auditing and verification expenses.

#### 6.5.2. Legal and Political Constraints.

**(1) Legal Boundaries**:
  (a) Anti-discrimination concerns: Differential treatment must be justifiable.
  (b) Contract enforcement: Mandates must be legally binding.
  (c) International harmonization: Cross-border projects face conflicting requirements.
**(2) Political Boundaries**:
  (a) Perceived fairness: Even if efficient, tiered policies must be seen as legitimate.
  (b) Lobbying pressures: Platform companies may resist higher mandates.
  (c) Coalition building: Need for broad ecosystem support.

### 6.6. Adaptive Governance Framework

**Fig 12:**
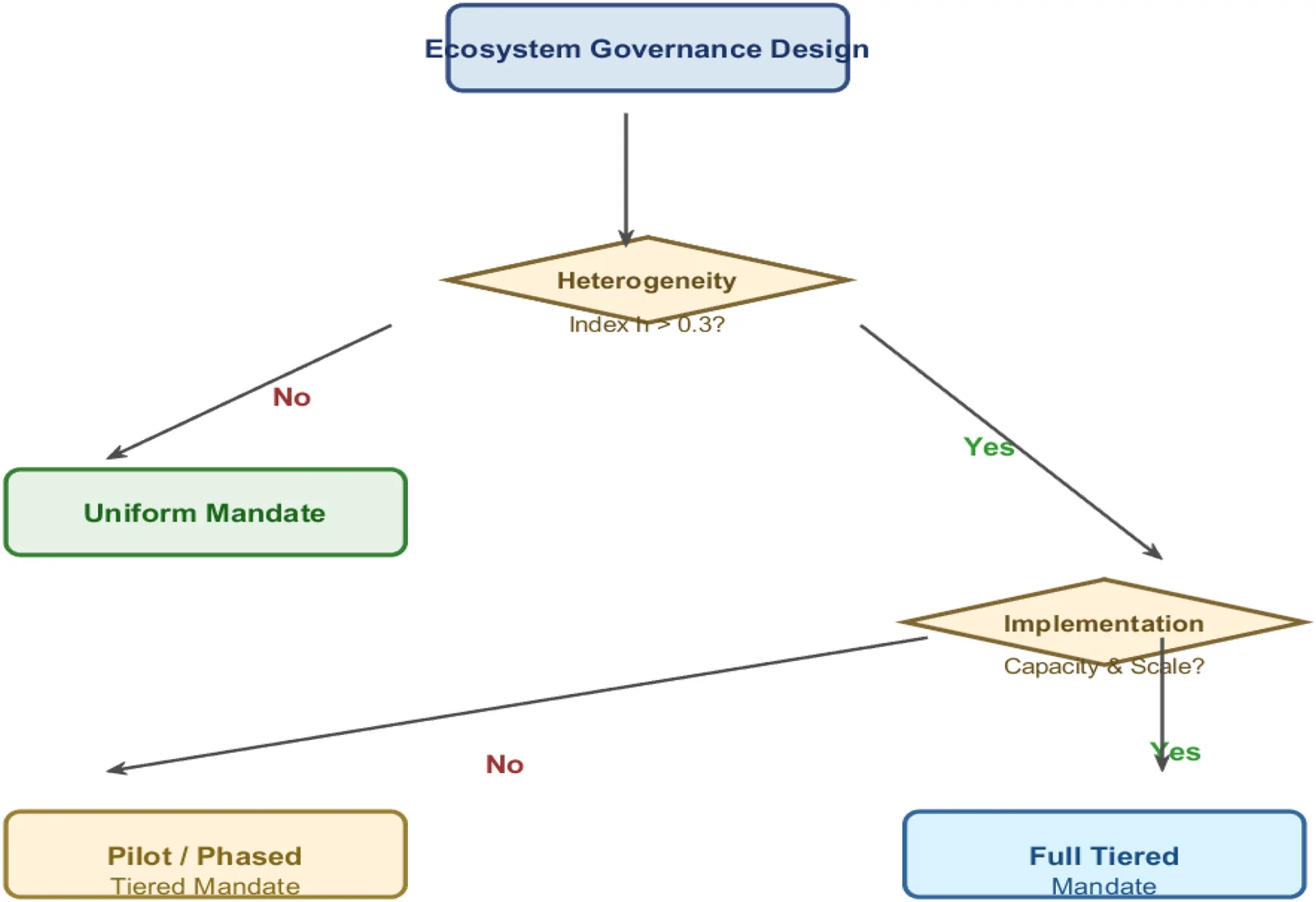
Adaptive Governance Decision Tree.

Given these boundary conditions, we propose an adaptive governance framework:

The framework, as shown in Fig 12, provides an adaptive decision process for tiered open-source mandates, guiding ecosystem architects through sequential assessments of heterogeneity (based on compliance costs and private value differentials) and implementation capacity/ecosystem scale, with evidence-based thresholds determining optimal policy choices from uniform to fully tiered systems as ecosystems evolve.

### 6.7. Parameter Sensitivity and Robustness Analysis

This section systematically examines the sensitivity of our key findings to parameter perturbations, addressing the fundamental concern that policy recommendations should not depend critically on specific numerical assumptions. We conduct three levels of sensitivity analysis: (1) individual parameter variations, (2) correlated parameter shifts reflecting realistic scenarios, and (3) extreme value testing.

#### 6.7.1. Individual Parameter Perturbations.

Applying Table 4, we analyze the robustness of the health advantage of tiered mandates ($\Delta V_{A}$) to ±20% variations in each key parameter while holding the others at baseline values.

**Table 4:** Sensitivity of tiered mandate advantage to parameter variations (±20%).

Parameter	Baseline Value	−20% $\Delta V_{A}$	+20% $\Delta V_{A}$	Sensitivity Index*	Robustness
$\lambda_{D}\;$	0.8	+18%	−15%	0.83	Moderate Stability
$\lambda_{I}$	0.2	−12%	+14%	0.65	Moderate Stability
$\rho_{D}$	2.0	−8%	+10%	0.45	Highly robust
$\rho_{I}$	0.8	+9%	−7%	0.40	Highly robust
$\gamma_{DI}$	1.2	+5%	−4%	0.23	Highly robust
$\gamma_{ID}$	1.5	−6%	+7%	0.33	Highly robust
β	0.4	+4%	−3%	0.18	Highly robust

*Sensitivity Index= $\left|\Delta \left(\Delta V_{A}\right)\left|/\right|\Delta \text{parameter}\right|$ normalized. SI<0.5: Highly robust; 0.5≤SI<1.0: Moderate Stability; SI≥1.0: fragile.

Our results demonstrate moderate to high robustness to parameter uncertainty.

#### 6.7.2. Monte Carlo robustness analysis.

**Fig 13:**
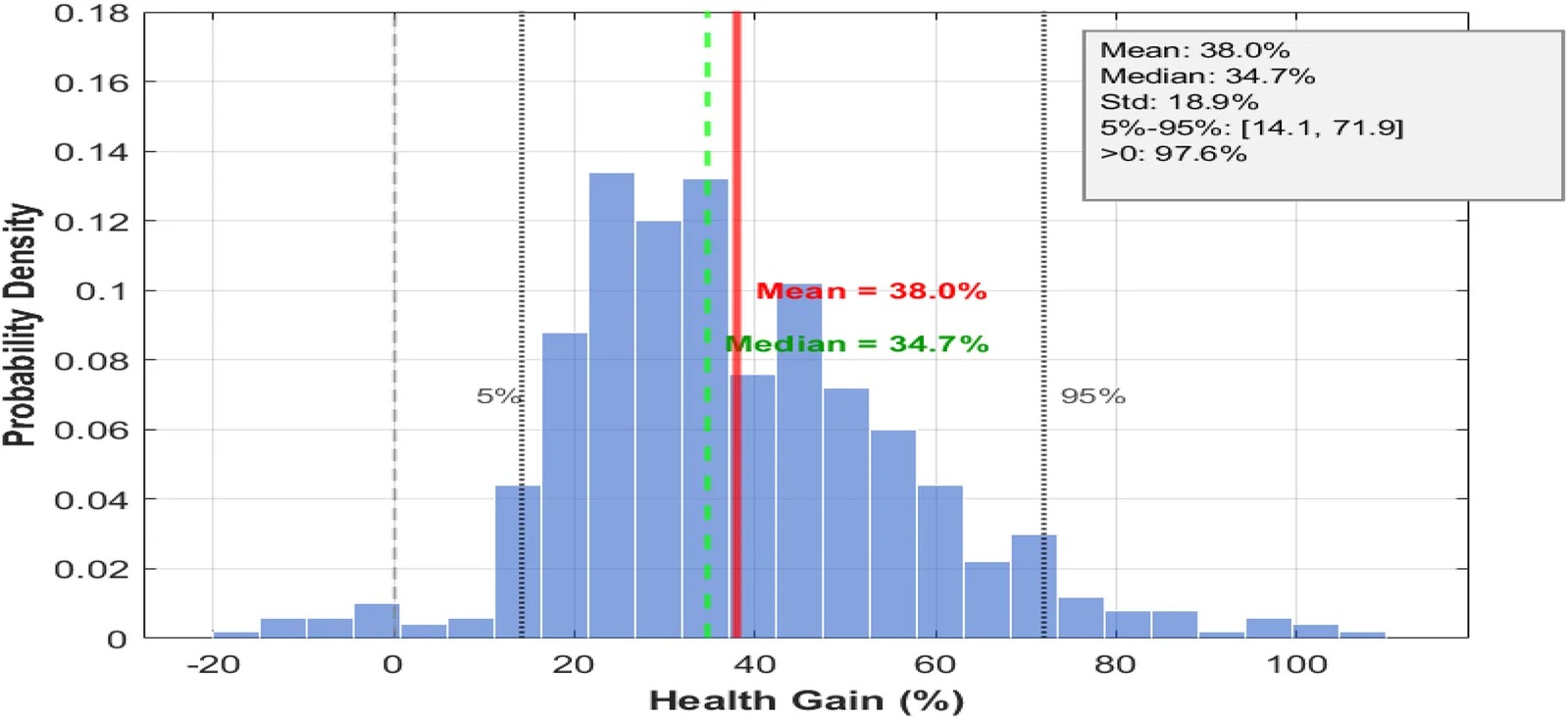
Robust Monte Carlo analysis.

We conduct a Monte Carlo simulation with 10,000 random parameter draws from uniform distributions covering ±30% of baseline values:

Fig 13 presents the results of a Monte Carlo robustness analysis with 500 random parameter draws, where each of the key structural parameters ($\lambda_{D}$, $\lambda_{I}$, $\rho_{D}$, $\rho_{I}$, $\gamma_{DI}$, $\gamma_{ID}$, β) is independently perturbed by ±15% from its baseline value. The distribution of health gains exhibits a clear right skew: the mean gain of 38.0% exceeds the median of 34.7%, and the 5th–95th percentile interval spans from 14.1% to 71.9%. Remarkably, 97.6% of the draws yield positive gains, and 97.0% yield gains exceeding 10%, indicating that the qualitative superiority of tiered mandates is not an artifact of specific parameter calibration but a structurally robust feature of the model. The narrow interquartile range (25.1% to 49.4%) further demonstrates that the magnitude of the tiered advantage remains substantial across the vast majority of parameter configurations. These findings provide strong empirical support for the policy recommendation that ecosystem architects should adopt tiered mandates rather than uniform rules when structural heterogeneity is present, as the benefits of differentiation persist across a wide range of plausible parameter values.

## 7. Conclusion

### 7.1. Key findings

The numerical magnitudes reported below (e.g., 15–40% health improvements, specific mandate rates) are model-based illustrations under a calibrated parameter set, not empirical estimates. Their main value is showing the direction and relative magnitude of effects, not providing precise policy numbers for direct implementation. Our differential game analysis yields five principal findings regarding open-source governance in heterogeneous AI ecosystems:

(1) Systematic superiority of tiered mandates: Tiered open-source mandates demonstrably outperform uniform mandates when significant heterogeneity exists between platform and independent projects, with health advantages robust across plausible parameter ranges.
(2) Quantifiable advantage range: The ecosystem health advantage of tiered mandates spans 15–40%, peaking when compliance cost asymmetry is pronounced (λD/λI>2.0), providing empirically measurable benchmarks for policy evaluation.
(3) Counter-intuitive optimal differentiation: Optimal policy reverses the “level playing field” intuition, imposing higher requirements on resource-rich platforms ($\theta_{D}\approx 0.68$) while protecting independent innovation through lower mandates ($\theta_{I}\approx 0.35$).
(4) Network effect amplification: Positive feedback loops emerge under tiered regimes, where platform contributions to the commons stimulate independent innovation, which in turn enhances ecosystem value, a virtuous cycle that uniform mandates fail to activate.
(5) Calibration imperative: Effective implementation requires continuous diagnostics of ecosystem parameters (compliance costs, private values, network dependencies) and dynamic recalibration mechanisms.

These findings provide actionable, evidence-based guidance for stewarding open-source AI infrastructure beyond intuitive heuristics.

### 7.2. Managerial and Policy Implications

#### 7.2.1. For Ecosystem Architects (Foundations, Consortia, Governments).

(1) Adopt tiered frameworks: Transition from one-size-fits-all to differentiated requirements based on project type and capacity.

(2) Evidence-based calibration: Ground mandate levels in systematic assessment of compliance cost structures, monetization potentials, and network dependencies.

(3) Dynamic governance mechanisms: Institute regular review cycles to adjust mandate rates as ecosystems evolve.

(4) Transparent justification: Clearly articulate the rationale for differential treatment to maintain legitimacy and community trust.

#### 7.2.2. For Platform Companies.

(1) Strategic contribution optimization: Recognize that measured increases in open-source contributions can expand total ecosystem value, potentially enlarging addressable markets.

(2) Compliance efficiency investment: Develop tooling and processes to reduce compliance costs ($\lambda_{D}$), thereby mitigating the burden of higher mandates.

(3) Constructive policy engagement: Advocate for governance frameworks that acknowledge platform scale advantages while preserving competitive diversity.

#### 7.2.3. For Independent Projects.

(1) Advocate for proportionate treatment: Highlight the disproportionate burden uniform mandates impose on resource-constrained entities.

(2) Demonstrate distinctive value: Emphasize unique contributions to innovation diversity and ecosystem resilience.

(3) Proactive compliance strategy: Anticipate evolving governance landscapes and develop adaptive contribution approaches.

#### 7.2.4. Implementation Roadmap.

(1) Diagnostic Phase: Map ecosystem architecture and quantify key parameters through surveys, repository analysis, and stakeholder interviews.

(2) Design Phase: Simulate alternative mandate structures using calibrated models to predict impacts and identify optimal differentiation.

(3) Pilot Phase: Implement tiered mandates in contained contexts with robust monitoring and evaluation frameworks.

(4) Scaling Phase: Refine and expand based on pilot evidence, establishing periodic recalibration mechanisms.

### 7.3. Limitations and Future Research Directions

While providing a rigorous analytical foundation, our study has limitations that delineate productive avenues for further investigation:

(1) Dynamic parameter evolution: Future models could incorporate learning-by-doing effects where compliance costs decrease endogenously with experience.
(2) Categorical refinement: Real ecosystems exhibit more nuanced project typologies; multi-tier or continuous classification schemes warrant exploration.
(3) Endogenous ecosystem dynamics: Incorporating project entry, exit, and boundary-spanning decisions would enhance model realism and policy relevance.
(4) Strategic classification behavior: The potential for projects to game classification systems presents both a theoretical challenge and practical governance consideration.
(5) Empirical validation and calibration: While parameterized from available evidence, systematic testing against real ecosystem data remains essential for refining theoretical predictions.
(6) Measurement and implementation: The gap between theoretical constructs (e.g., “effective open-source knowledge stock”) and measurable proxies requires further operationalization research. Future work should develop and validate empirical metrics for the key state variables.
(7) Continuous type space: Our binary classification of projects as either “platform” or “independent” is a modeling simplification. In reality, projects such as Hugging Face occupy intermediate positions along a spectrum, combining venture capital backing with strong community engagement. The binary model can be understood as the limiting case of a continuum where the two types represent poles of the distribution. We conjecture that the core qualitative result- that differentiation improves ecosystem health when heterogeneity is present- would extend to a continuous type space. However, the optimal policy would then involve a smoothly graduated mandate schedule rather than a discrete two-tier structure. Specifically, projects with higher monetization capacity and compliance cost elasticity would face progressively higher openness requirements, while those with lower capacity would receive progressively lower mandates. This extension is technically non-trivial and a promising direction for future research. However, the binary formulation lets us derive sharp analytical insights into the structural conditions under which differentiation is most valuable, without obscuring them in the complexity of a continuous screening problem.

Despite these limitations, our analysis formally establishes that differentiated open-source governance, rooted in the inherent structural heterogeneities of AI ecosystems, represents a promising pathway to optimize innovation at the infrastructure layer, the precise convergence point of technological sovereignty and economic competitiveness.

While our model is explicitly specified for AI foundation ecosystems, its general structure is readily applicable to any two-tier open-source ecosystem. Future extensions could integrate AI-specific production functions (e.g., compute-data-model complementarities). However, the current framework deliberately adopts a parsimonious design to preserve full analytical tractability, as the first dynamic game model formalizing tiered open-source governance.

## Supporting information

S1 Appendix Appendix of this Article (DOCX)
