# Supplementary material for: Can tiered open-source mandates optimize innovation? A differential game study osn platform vs. independent project dynamics in foundation software ecosystems
Source: PLoS One. 2026 Sep 11;21(9):e0340713. doi: 10.1371/journal.pone.0340713 (PMC13567796; doi:10.1371/journal.pone.0340713)
Supplement: Supplementary file 1 [file pone.0340713.s001.docx]

**Appendix A: Proofs and Derivations**

This appendix provides formal proofs and derivations for the propositions stated in the main text. Unless otherwise noted, all functions are assumed to be continuously differentiable on the compact state space $S=[0,\overline{K}]^{2}$ with $\overline{K}$ sufficiently large. All parameters are strictly positive unless explicitly stated otherwise.

**A.1 Proof of Proposition 4.1 (Comparative Statics of Follower Effort)**

We derive the comparative statics for the follower's optimal effort with respect to the mandate rate. Consider the platform project's optimization problem; the independent project's case is symmetric.

Step 1: Express the optimal effort.

From the first-order condition of the follower's HJB equation, the optimal effort is

$$e^{*} (\theta)=\frac{\rho(1-\theta) +V_{K\eta}\theta}{c (1+\lambda\theta) }$$

To simplify notation, define: $A=\rho$ (private appropriation benefit); $B=V_{K \eta}$(marginal value of knowledge stock times R&D efficiency); $C=c$ (base R&D cost coefficient), $\lambda=\text{ }compliance\text{ }cost\text{ }multiplier$.

Then the optimal effort becomes

$$e^{*} (\theta)=\frac{A (1-\theta) +B\theta}{C (1+\lambda\theta) }$$

Step 2: Take the derivative with respect to $\theta$. Apply the quotient rule:

$\frac{de^{*}}{d\theta}=\frac{(-A+B)\cdot(1+\lambda\theta)-[A(1-\theta)+B\theta]\cdot\lambda}{C(1+\lambda\theta)^{2}}$.

Step 3: Simplify the numerator. Let the numerator be $N$. Then:

$N=(-A+B)(1+\lambda\theta)-\lambda A(1-\theta)-\lambda B\theta$. Expand the first term: $N=(-A+B)+(-A+B)\lambda\theta-\lambda A+\lambda A\theta-\lambda B\theta$. Group terms by powers of $\theta$: Constant terms (no $\theta$): $(-A+B)-\lambda A=B-A-\lambda A=B-A(1+\lambda)$. $\theta$ terms

$(-A+B)\lambda\theta+\lambda A\theta-\lambda B\theta=(-A\lambda+B\lambda+A\lambda-B\lambda)\theta=0$. Thus: $N=B-A(1+\lambda)$

Step 4: Write the final derivative.

Substituting back: $\frac{de^{*}}{d\theta}=\frac{B-A(1+\lambda) }{C(1+\lambda\theta)^{2}}$.

Step 5: Substitute the original parameters. For the platform project:

$\frac{\partial e_{D}^{*}}{\partial\theta_{D}}=\frac{V_{D,K_{D}}\eta_{D}-\rho_{D}(1+\lambda_{D})}{c_{D}(1+\lambda_{D}\theta_{D})^{2}}$. For the independent project: $\frac{\partial e_{I}^{*}}{\partial\theta_{I}}=\frac{V_{I,K_{I}}\eta_{I}-\rho_{I}(1+\lambda_{I})}{c_{I}(1+\lambda_{I}\theta_{I})^{2}}$

Step 6: Interpretation and sign of the net effect.

The sign of the derivative is determined by the numerator: If, then $\partial e_{i}^{*} /\partial\theta_{i} >0$. Interpretation: The marginal value of accumulating more knowledge stock (weighted by R&D efficiency) exceeds the marginal private return from proprietary code adjusted for compliance costs. In this case, a higher mandate rate increases R&D effort because the entity values the future benefits of a larger commons more than the current foregone private returns.

If, then $\partial e_{i}^{*} /\partial\theta_{i} <0$. Interpretation: The private return from keeping code proprietary dominates. A higher mandate suppresses R&D effort because the entity cannot capture enough of the value it creates.

If, then $\partial e_{i}^{*} /\partial\theta_{i}=0$. The two effects exactly cancel.

Step 7: The ambiguous net effect.

Because is endogenous and depends on the entire future trajectory, the sign of $\partial e_{i}^{*} /\partial\theta_{i}$ cannot be determined ex ante. This ambiguity is the key economic insight: a higher mandate has two countervailing effects: Direct disincentive effect (captured by $-\rho_{i }\theta_{i}$ in the numerator of $e^{*}$): Higher $\theta_{i}$ means less of the output can be kept proprietary, reducing the immediate return from effort.

Indirect incentive effect (captured by): Higher $\theta_{i}$ means more open-source contribution flows into the commons, increasing the stock $K_{i}$, which yields future benefits.

The net effect depends on the entity's valuation of the future (through) relative to its current private returns ($\rho_{i }$) and compliance burden ($\lambda_{i }$).

**A.2 Proof of Proposition 4.0 (Existence and Uniqueness of Feedback Equilibrium)**

We establish existence and uniqueness of the Markov-perfect equilibrium for the differential game. The proof follows the standard contraction mapping approach in Dockner et al. (2000, Chapter 7) adapted to our three-player Stackelberg structure.

Step 1: State space and assumptions.

Define the compact state space $S=[0,\overline{K}]^{2}$, where $\overline{K}$ is chosen sufficiently large such that the optimal trajectories never reach the boundary. This is guaranteed by the following assumptions:

Assumption 1 (Positivity and boundedness): $r>0$, $\delta>0$, and all payoff parameters ($\rho_{i }$, $c_{i}$, $\lambda_{i}$, $\eta_{i}$, $\alpha_{i}$) are strictly positive and finite.

Assumption 2 (Concavity): The instantaneous payoff functions $\Pi_{i}$ and HH are strictly concave in their respective control variables and jointly concave in the state variables.

Assumption 3 (Lipschitz continuity): The state dynamics are Lipschitz continuous in ($K_{D} ,K_{I} ,e_{D} ,e_{I}$).

Assumption 4 (Nondegeneracy): The marginal value is bounded away from zero for all $K_{i}=[0,\overline{K}]$.

These assumptions hold for our calibrated parameter set and all robustness checks.

Step 2: Followers' HJB equations.

For a fixed mandate policy $\theta$ (or ($\theta_{D}$,$\theta_{I}$), the followers' HJB equations are:

$rV_{D}=\max_{e_{D}\geq0} \left\{\alpha_{D}\left[\gamma_{DD}K_{D} +\gamma_{ID}K_{I}\right]+\rho_{D}(1-\theta) e_{D}-\frac{1}{2}c_{D}(1+\lambda_{D}\theta) e_{D}^{2}+V_{D,K_{D}}(\eta_{D}\theta_{D}e_{D}-\delta K_{D})+V_{D,K_{I}}(\eta_{I}\theta_{I}e_{I}^{*}-\delta K_{I})\right\}$ $rV_{I}=\max_{e_{I}\geq0} \left\{\alpha_{I}\left[\gamma_{II}K_{I} +\gamma_{DI}K_{D}\right]+\rho_{I}(1-\theta) e_{I}-\frac{1}{2}c_{I}(1+\lambda_{I}\theta) e_{I}^{2}+V_{I,K_{D}}(\eta_{D}\theta_{D}e_{D}^{*}-\delta K_{D})+V_{I,K_{I}}(\eta_{I}\theta_{I}e_{I}-\delta K_{I})\right\}$

Given $e_{I}^{*}$ in the first equation and $e_{D}^{*}$ in the second, each follower's optimization is a standard optimal control problem with a single control. By Theorem 3.1 of Fleming and Rishel (1975), each HJB equation admits a unique viscosity solution in the class of continuously differentiable functions on SS because:

The control set $e_{i}=[0,\overline{e_{i}}]$ is compact, the reward functions are concave in $e_{i}$ (quadratic with negative coefficient), the state dynamics are linear in $e_{i}$, The discount rate$r>0$ ensures finite values.

Thus, for a fixed mandate policy, the followers' value functions $V_{D}$ and $V_{I}$ are uniquely determined.

Step 3: Architect's HJB equation.

The architect's HJB equation is:

$rV_{D}=\max_{\theta_{D},\theta_{i}} \left\{H(K_{D},K_{I})_{D}+V_{A,K_{D}}(\eta_{D}\theta_{D}e_{D}^{*}-\delta K_{D})+V_{A,K_{I}}(\eta_{D}\theta_{D}e_{I}^{*}-\delta K_{I})\right\}$,

where $e_{D}^{*}$ and $e_{I}^{*}$ are the followers' best responses derived in Step 2. Substituting the explicit expressions for $e_{i}^{*}$ yields a PDE that is continuous in ($\theta_{D}$,$\theta_{I}$).

Step 4: Policy iteration as a contraction mapping.

Define the policy improvement operator $T$ as follows: for a given policy $(\theta_{D}^{(K)},\theta_{I}^{(K)})$, solve the followers' HJB equations to obtain $(V_{D}^{(K)},V_{I}^{(K)})$, then solve the architect's HJB to obtain ($V_{A}^{(K)}$), and finally update the policy using the first-order conditions:

$\theta_{i}^{K}=\arg\max_{\theta_{i}} \left\{V_{A,K_{i}}^{(K)}\eta_{i}e_{i}^{*}(\theta_{i })\right\}$.

The operator $T$ has the following contraction property. Let $\parallel\cdot\parallel_{\infty}$ denote the sup-norm on $S$. For any two policies $(\theta_{D},\theta_{I})$ and $(\overrightarrow{\theta}_{D},\overrightarrow{\theta}_{I})$:$\parallel T(\theta)-T(\overrightarrow{\theta})\parallel_{\infty}\leq\frac{1}{r+\delta} \parallel\theta-\overrightarrow{\theta}\parallel_{\infty}$.

The contraction coefficient is $1\text{/}(r+\delta)<1$ since $r>0$ and $\delta>0$. The inequality follows from: The value functions are Lipschitz in the policies (because the payoffs are smooth); The state dynamics are Lipschitz in the policies; the discounting over the infinite horizon reduces the impact of any policy difference by the factor $1/(r+\delta)$.

Step 5: Existence of a fixed point.

By the Banach fixed-point theorem, the contraction mapping T has a unique fixed point on the complete metric space of bounded continuous functions on S. This fixed point corresponds to the unique policy $(\theta_{D}^{*},\theta_{I}^{*})$ and the associated value functions $(V_{D}^{*},V_{I}^{*},V_{A}^{*})$.

Step 6: Verification that the fixed point is the equilibrium.

The fixed point satisfies the HJB equations for all players and the first-order conditions, which are sufficient for optimality due to the concavity of the Hamiltonians (the Mangasarian sufficient conditions hold). Thus, the fixed point is the unique Markov-perfect feedback equilibrium.

Conclusion: Under Assumptions 1–4, the system of HJB equations admits a unique viscosity solution, and the feedback strategies derived from the first-order conditions constitute a unique Markov-perfect equilibrium.

**A.3 Proof of Proposition 4.2 (Optimal Mandate Structure)**

We prove that under the tiered regime, the optimal mandate rates satisfy $V_{D}^{*}>V_{I}^{*}$ when $\lambda_{D}>\lambda_{I}$ and $\rho_{D}>\rho_{I}$, under the additional assumption that V$V_{D,K_{D}\eta_{D}}$ and $V_{I,K_{I}\eta_{I}}$ are not too different.

Step 1: Characterize the optimal mandate for each type.

From the architect's first-order conditions (Equation 13 in the main text):

. Substituting: $\eta_{i} (e_{i}^{*} +\theta_{i} \frac{\partial e_{i}^{*}}{\partial\theta_{i}} )=0$

Since $\eta_{i}>0$ this simplifies to: $e_{i}^{*} +\theta_{i} \frac{\partial e_{i}^{*}}{\partial\theta_{i}}=0$

Step 2: Substitute the expressions for $e_{i}^{*}$ and its derivative.

From A.1, we have: $e_{i}^{*} =\frac{\rho_{i} (1-\theta_{i}) +S_{i}\theta_{i} }{c_{i} (1+\lambda_{i} \theta_{i}) }$, where , and ρ$\frac{\partial e_{i}^{*}}{\partial\theta_{i}}=\frac{S_{i}-\rho_{i} (1-\theta_{i}) }{c_{i} (1+\lambda_{i} \theta_{i})^{2} }$.

Step 3: Derive the optimality condition.

$e_{i}^{*}+\theta_{i} \partial e_{i}^{*} /\partial\theta_{i} =0$: $\frac{\rho_{i} (1-\theta_{i}) +S_{i}\theta_{i} }{c_{i} (1+\lambda_{i} \theta_{i}) }+\theta\frac{S_{i}-\rho_{i} (1-\theta_{i}) }{c_{i} (1+\lambda_{i} \theta_{i})^{2} }=0$.

Multiply through by $c_{i} (1+\lambda_{i} \theta_{i})^{2}$: $[\rho_{i} (1-\theta_{i} )+S_{i} \theta_{i }](1+\lambda_{i} \theta_{i} )+\theta_{i }[S_{i} -\rho_{i}(1+\lambda_{i} )]=0$.

Step 4: Simplify algebraically.

Let $S_{i}=V_{i,K_{i}\eta_{i}}$ and expand: First term: $\rho_{i} (1-\theta_{i })(1+\lambda_{i} \theta_{i })+S_{i} \theta_{i} (1+\lambda_{i} \theta_{i } )$; Second term: $\theta_{i }S_{i }-\theta_{i} \rho_{i }(1+\lambda_{i} )$. Summing: $\rho_{i} (1-\theta_{i })(1+\lambda_{i} \theta_{i })-\theta_{i} \rho_{i }(1+\lambda_{i} )+S_{i} \theta_{i} (1+\lambda_{i} \theta_{i } )+\theta_{i }S_{i }=0$. Now simplify the $\rho_{i}$ terms: $\rho_{i}\left[(1-\theta_{i })(1+\lambda_{i} \theta_{i })-\theta_{i} (1+\lambda_{i} \right]$. Compute $(1-\theta_{i })(1+\lambda_{i} \theta_{i })$: $=1+\lambda_{i} \theta_{i } -\theta_{i }-\lambda_{i }\theta_{i}^{2}$. Subtract $\theta_{i} (1+\lambda_{i} )=\theta_{i} +\lambda_{i }\theta_{i}$: $=1+\lambda_{i} \theta_{i } -\theta_{i } -\lambda_{i }\theta_{i}^{2} -\theta_{i} -\lambda_{i} \theta_{i} =1-2\theta_{i }-\lambda_{i }\theta_{i}^{2}$

So the $\rho_{i}$ terms become: $\rho_{i}(1-2\theta_{i }-\lambda_{i }\theta_{i}^{2})$.

Now simplify the $S_{i}$ terms: $S_{i }\theta_{i} (1+\lambda_{i} \theta_{i } )+\theta_{i} S_{i }=S_{i }\theta_{i} +S_{i }\lambda_{i}\theta_{i}^{2} +\theta_{i} S=2\theta_{i} S +_{i }\lambda_{i}\theta_{i}^{2}$. Thus the full equation is: $\rho_{i} (1-2\theta_{i }-\lambda_{i }\theta_{i}^{2} )+S_{i} (2\theta_{i }+\lambda_{i }\theta_{i}^{2} )=0$.

Step 5: Rearrange into a quadratic equation.

Bring all terms together: $\rho_{i}-2\rho_{i}\theta_{i }-\rho_{i}\lambda_{i }\theta_{i}^{2} +2S_{i} \theta_{i }+S_{i} \lambda_{i }\theta_{i}^{2} =0$. Group by powers of $\theta_{i }$:

Constant: $\rho_{i}$; $\theta_{i }$: $2(S_{i}-\rho_{i})\theta_{i }$; $\theta_{i}^{2}$: $(S_{i}-\rho_{i})\lambda_{i }\theta_{i}^{2}$.

Thus: $(S_{i}-\rho_{i})\lambda_{i }\theta_{i}^{2}+2(S_{i}-\rho_{i})\theta_{i }+\rho_{i}=0$. Let $D_{i}=S_{i}-\rho_{i}$. Then: $D_{i}\lambda_{i }\theta_{i}^{2}+2D_{i}\theta_{i }+\rho_{i}=0$.

Step 6: Solve the quadratic equation.

If $D_{i}=0$, then the equation reduces to $\rho_{i}=0$, which is impossible (since $\rho_{i}>0$). Thus $D_{i}\neq0$. Divide by $D_{i}\lambda_{i }$: $\theta_{i}^{2}+\frac{2}{\lambda_{i }}\theta_{i }+\frac{\rho_{i}}{D_{i}\lambda_{i}}=0$. Apply the quadratic formula:$\theta_{i }=\frac{-\frac{2}{\lambda_{i }}\pm\sqrt{\frac{4}{\lambda_{i}^{2}}-\frac{4\rho_{i}}{D_{i}\lambda_{i}}}}{2}$ Simplify: $\theta_{i }=\frac{1}{\lambda_{i }}(-1\pm\sqrt{1-\frac{\lambda_{i}\rho_{i}}{D_{i}}})$.

Since $\theta_{i} \in[0,1]$ and $\rho_{i }>0$, $\lambda_{i }>0$, we require$D_{i}>\lambda_{i}\rho_{i}$ for a real solution. The relevant (positive) root is: $\theta_{i}^{*}=\frac{1}{\lambda_{i }}(-1+\sqrt{1-\frac{\lambda_{i}\rho_{i}}{D_{i}}})$.

Step 7: Comparative statics on $\theta_{i}$.

We examine how $\theta_{i}^{*}$ depends on the key parameters.

(a) Effect of $\rho_{i}$ (private value): Let $F(D_{i} ,\rho_{i} ,\lambda_{i })=-1+\sqrt{1-\frac{\rho_{i}\lambda_{i}}{D_{i}}}$. Since $\theta_{i}^{*}=F/\lambda_{i }$, and $\lambda_{i }>0$, the sign of $\partial\theta_{i}^{*}/\partial\rho_{i}$ is the same as the sign of $\partial F/\partial\rho_{i}$. Compute:

$\frac{\partial F}{\partial\rho_{i}}=\frac{1}{2\sqrt{1-\rho_{i}\lambda_{i}/D_{i}}}(-\frac{\lambda_{i}}{D_{i}})=-\frac{\lambda_{i}}{2D_{i}\sqrt{1-\rho_{i}\lambda_{i}/D_{i}}}$. Since $D_{i}>0$ (we established $D_{i}>\rho_{i}\lambda_{i}>0$), this derivative is negative. Therefore: $\frac{\partial\theta_{i}^{*}}{\partial\rho_{i}}<0$.

Actually, from the original equation $e_{i}^{*} +\theta_{i} \frac{\partial e_{i}^{*}}{\partial\theta_{i}}=0$, increasing $\rho_{i}$ raises the marginal benefit of effort, which tends to increase $e_{i}^{*}$; but it also raises the cost of the mandate (because more private value is foregone). The net effect is ambiguous and depends on the magnitude of $S_{i}$. We rely on the numerical result from the calibrated model: with our parameters, the dominant effect is that $\theta_{D}>\theta_{I}$.

(b) Effect of $\lambda_{i}$ (compliance cost): Taking the derivative with respect to $\lambda_{i}$ (holding $D_{i}$ and $\rho_{i}$ fixed): $\frac{\partial\theta_{i}^{*}}{\partial\lambda_{i}}=\frac{1}{\lambda_{i}^{2}}(-1+\sqrt{1-\frac{\lambda_{i}\rho_{i}}{D_{i}}})+\frac{1}{\lambda_{i}}\frac{1}{2\sqrt{1-\rho_{i}\lambda_{i}/D_{i}}}(-\frac{\rho_{i}}{D_{i}})$. The first term is negative (since $\sqrt{1-\rho_{i}\lambda_{i}/D_{i}}<1$, so $-1+\sqrt{\cdot}<0$). The second term is also negative. Thus: $\theta_{i}^{*} /\partial\lambda_{i} <0$.

Interpretation: Higher compliance costs reduce the optimal mandate rate because the cost of enforcing the mandate is higher.

(c) Effect of $S_{i}$ (marginal value of knowledge): Taking the derivative with respect to $S_{i}$ (holding $\rho_{i}$ and $\lambda_{i}$ fixed): $\frac{\partial\theta_{i}^{*}}{\partial S_{i}}=\frac{1}{\lambda_{i}}\frac{1}{2\sqrt{1-\rho_{i}\lambda_{i}/D_{i}}}\frac{\rho_{i}\lambda_{i}}{D_{i}^{2}}\frac{\partial D_{i}}{\partial S_{i}}$. Since $\partial D_{i}/\partial S_{i}=1$, and all other factors are positive, we have: $\partial\theta_{i}^{*} /\partial S_{i} >0$.

Interpretation: When the marginal value of accumulating knowledge is higher, the optimal mandate is higher because the benefits of a larger commons are larger.

Step 8: Compare $\theta_{D}^{*}$ and $\theta_{I}^{*}$.

We have: $\lambda_{D}>\lambda_{I}$ (platforms have higher compliance costs). This tends to make $\theta_{D}^{*}<\theta_{I}^{*}$ (because $\theta_{i}^{*} /\partial\lambda_{i} <0$); $\rho_{D}>\rho_{I}$ (platforms have higher private value). This tends to make $\theta_{D}^{*}>\theta_{I}^{*}$ (if the total derivative is positive); $S_{D}=V_{D,K_{D}\eta_{D}}$ vs $S_{I}=V_{I,K_{I}\eta_{I}}$: typically $S_{I}>S_{D}$ (independents value the commons more). This tends to make $\theta_{D}^{*}<\theta_{I}^{*}$ (because $\partial\theta_{i}^{*} /\partial S_{i} >0$).

The net comparison depends on the relative magnitudes. In our calibrated baseline: $\lambda_{D}=0.8$, $\lambda_{I}=0.2$, $\rho_{D}=0.2$, $\rho_{I}=0.8$, $S_{D}$ and $S_{I}$ are determined endogenously.

The numerical solution yields $\theta_{D}^{*}\approx0.68>0.35\simeq\theta_{I}^{*}$, meaning the $\rho$ effect dominates.

Condition for $\theta_{D}^{*}>\theta_{I}^{*}$:

From the quadratic solution, $\theta_{D}^{*}>\theta_{I}^{*}$ is equivalent to:

.

While a closed-form threshold is not analytically tractable, numerical evaluation confirms that this inequality holds when: $\frac{\rho_{D}}{\rho_{I}}=\frac{1+\lambda_{D}}{1+\lambda_{I}}\frac{S_{I}}{S_{D}}\Phi(\lambda_{D},\lambda_{I},\rho_{D},\rho_{I})$, where $\Phi\in(0,1)$ is a correction factor. For our baseline parameters, the left-hand side is 2.0/0.8=2.5, the right-hand side is approximately $1.8/1.2\times(S_{I} /S_{D} )\times\Phi$. With $S_{I} /S_{D}\approx1.5$ and $\Phi\approx0.7$, the right-hand side is about 1.5×1.5×0.7=1.575, so the inequality holds.

Conclusion: Under the condition that the private appropriation differential ($\rho_{D}/\rho_{I}$) is sufficiently large relative to the compliance cost differential ($\lambda_{D}/\lambda_{I}$) and the knowledge valuation differential ($S_{I}/S_{D}$), we have $\theta_{D}^{*}>\theta_{I}^{*}$. This condition is satisfied in our calibrated baseline.

**A.4 Comparative Statics for the Architect's Uniform Policy**

We provide the sensitivity of the optimal uniform mandate $\theta^{*}$ to the key parameters. This analysis demonstrates why the uniform policy is a constrained compromise.

Step 1: The first-order condition.

From the architect's FOC under the uniform policy (Equation 10 in the main text):

.

Step 2: . Using : .

From A.1, we know: $\frac{\partial e^{*}}{\partial\theta}=\frac{S_{i}-\rho_{i}(1+\lambda) }{c_{i}(1+\lambda\theta)^{2}}$, where $S_{i}=V_{i,K_{i}\eta_{i}}$. Thus:

. Define $\Phi_{i}(\theta)$ as the term in brackets. Then: .

Step 3: The FOC in compact form.

Substituting: $V_{A,K_{D}}\eta_{D}\Phi_{D}(\theta)+V_{A,K_{I}}\eta_{I}\Phi_{I}(\theta)=0$. Since $V_{A,K_{i}}>0$ and $\eta_{i }>0$, the optimal $\theta^{*}$ balances the two terms: $\Phi_{D}(\theta)$ is the marginal contribution of the mandate to platform knowledge growth; $\Phi_{I}(\theta)$ is the marginal contribution to independent knowledge growth.

Step 4: Implicit differentiation.

To find how $\theta^{*}$ changes with a parameter pp, differentiate the FOC with respect to $p$:

$(V_{A,K_{D}}\eta_{D}\Phi^{'}_{D}+V_{A,K_{I}}\eta_{I}\Phi'_{I})\frac{\partial\theta^{*}}{\partial p}+\frac{\partial}{\partial p}(V_{A,K_{D}}\eta_{D}\Phi_{D}+V_{A,K_{I}}\eta_{I}\Phi_{I})=0$.

The first term in parentheses is negative by the second-order condition (the FOC is a maximum). Therefore: $\frac{\partial\theta^{*}}{\partial p}=\frac{\frac{\partial}{\partial p}(V_{A,K_{D}}\eta_{D}\Phi_{D}+V_{A,K_{I}}\eta_{I}\Phi_{I})}{V_{A,K_{D}}\eta_{D}\Phi^{'}_{D}+V_{A,K_{I}}\eta_{I}\Phi'}$.

Step 5: Effect of $\lambda_{D}$ (platform compliance cost). Compute $\partial\Phi_{D}/\partial\lambda_{D}$:

Recall $\Phi_{D}(\theta)=\frac{\rho_{D}(1-\theta)+S_{D}\theta}{c_{D}(1+\lambda_{D}\theta)}+\theta\frac{S_{D}-\rho_{D}(1+\lambda_{D}) }{c_{D}(1+\lambda_{D}\theta)^{2}}$. For the first term, the derivative with respect to $\lambda_{D}$ is: $\frac{\partial}{\partial\lambda_{D}}\left[\frac{\rho_{D}(1-\theta)+S_{D}\theta}{c_{D}(1+\lambda_{D}\theta)}\right]=-\frac{[\rho_{D}(1-\theta)+S_{D}\theta]\theta}{c_{D}(1+\lambda_{D}\theta)^{2}}$. For the second term, use the quotient rule. Let $N=S_{D}-\rho_{D}(1+\lambda_{D})$, so $\partial N/\partial\lambda_{D}=\rho_{D}$. Then:

$\frac{\partial}{\partial\lambda_{D}}\left[\frac{\theta N }{c_{D}(1+\lambda_{D}\theta)^{2}}\right]=\frac{\theta(-\rho_{D} )c_{D} (1+\lambda_{D} \theta)^{2}-\theta N\cdot c_{D} \cdot2(1+\lambda_{D} \theta)\cdot\theta}{c_{D}^{2}(1+\lambda_{D}\theta)^{4}}$.

Simplifying: $\frac{-\rho_{D}\theta}{c_{D}(1+\lambda_{D}\theta)^{2}}-\frac{2N\theta^{2} }{c_{D}(1+\lambda_{D}\theta)^{3}}$. Thus $\partial\Phi_{D}/\partial\lambda_{D}<0$ for typical parameters (both terms are negative). Therefore, $\partial(V_{A,K_{D}}\eta_{D}\Phi_{D})/\partial\lambda_{D}<0$. Since the denominator is negative, we have: $\partial\theta^{*}/\partial\lambda_{D}<0$.

Interpretation: When platform compliance costs increase, the optimal uniform mandate decreases. The architect cannot raise the mandate for platforms without hurting them, so the uniform rule settles at a lower level to accommodate platforms.

Step 6: Effect of $\lambda_{I}$ (independent compliance cost).

Similarly, $\partial\Phi/\partial\lambda_{I}<0$, so $\partial\theta^{*}/\partial\lambda_{I}<0$.

Interpretation: Higher independent compliance costs also reduce the uniform mandate because the architect wants to protect independents.

Step 7: The uniform mandate as a compromise.

Under the uniform policy, a single $\theta$ must balance the needs of both types. If $\lambda_{D}\neq\lambda_{I}$ and $\rho_{D}\neq\rho_{I}$, the optimal $\theta$ lies between the optimal type-specific mandates:

$\min(\theta_{D}^{opt},\theta_{I}^{opt})\leq\theta^{uniform}\leq\max(\theta_{D}^{opt},\theta_{I}^{opt})$. This means: If $\theta_{D}^{opt}>\theta_{I}^{opt}$ (as in our calibration), the uniform mandate $\theta$ is lower than $\theta_{D}^{opt}$ (under-utilizing platforms) and higher than $\theta_{I}^{opt}$ (over-burdening independents). This is the key inefficiency that the tiered policy overcomes.

**Appendix B: Extensions and Robustness Details**

This appendix provides full derivations for the extensions summarized in Section 6 of the main text.

**B.1 Alternative Cost Function Specifications**

B.1.1 Linear-Quadratic Cost (without quadratic effort term)

Model specification: The cost is linear in effort (with a fixed marginal cost):

$\Pi_{i} =\alpha_{i} (\gamma_{ii} K_{i} +\gamma_{ji }K_{j} )+\rho_{i} (1-\theta_{i} )e_{i} -c_{i} (1+\lambda_{i} \theta_{i} )e_{i}$. Optimal effort decision:

The first-order condition is: $\rho_{i} (1-\theta_{i })+V_{i},_{K_{i} } \eta_{i} \theta_{i} -c_{i} (1+\lambda_{i} \theta_{i} )\leq0$, with complementary slackness: if the left-hand side is negative, $e_{i}^{*}=0$; if it is positive, effort is at its maximum; if equal to zero, effort is indeterminate (bang-bang). For an interior solution, we require: $\rho_{i} (1-\theta_{i })+V_{i},_{K_{i} } \eta_{i} \theta_{i} =c_{i} (1+\lambda_{i} \theta_{i} )$. Solving for $\theta_{i}$: $\rho_{i} -\rho_{i }\theta_{i }+S_{i} \theta_{i }=c_{i }+c_{i} \lambda_{i} \theta_{i}$, where $S_{i}=V_{A,K_{i}}\eta_{i}$.

Rearrange: $\rho_{i} -c_{i }+\theta_{i }(S_{i} -\rho_{i }-c_{i} \lambda_{i})=0$. Thus: $\theta_{i}^{linear}=\frac{ c_{i }-\rho_{i}}{S_{i} -\rho_{i }-c_{i} \lambda_{i}}$.

Feasibility constraint: The optimal effort is positive only if the marginal benefit of effort exceeds the marginal cost. When $\theta_{i}$ exceeds a threshold, effort drops to zero, creating a corner solution. This threshold occurs when: $\rho_{i }(1-\theta_{i} )+S{}_{i} \theta_{i }=c_{i} (1+\lambda_{i }\theta_{i })$. Solving for the effort-zero threshold $\overline{\theta}_{i}$: . If $\overline{\theta}_{i}<0$, effort is always positive; if $\overline{\theta}_{i}>1$, effort is always zero (uninteresting case). For typical parameters, $\overline{\theta}_{i}\in(0,1)$.

Optimal tiering under linear-quadratic cost:

The architect chooses $\theta_{D}$ and $\theta_{I}$ subject to the constraint that effort is nonnegative. The optimal solution features: $\theta_{D}$ is capped at $\overline{\theta}_{D}$ (platforms have higher $c_{D}$ and $\lambda_{D}$, so the cap is lower); $\theta_{I}$ is set lower than $\theta_{D}$ to preserve independent effort.

In our numerical example, $\theta_{D}^{linear}\approx0.55$, $\theta_{I}^{linear}\approx0.42$, yielding differentiation of 0.13.

**B.1.2 Power Cost Function**

Model specification: $\Pi_{i} =\alpha_{i} (\gamma_{ii} K_{i} +\gamma_{ji }K_{j} )+\rho_{i} (1-\theta_{i} )e_{i} -\frac{1}{p}c_{i} (1+\lambda_{i} \theta_{i} )e_{i}^{p}$, with $p>1$ (convex cost). The baseline quadratic model corresponds to $p=2$. Optimal effort decision:

The first-order condition is: $\rho_{i} (1-\theta_{i} +V_{i,k_{i}}\eta_{i}\theta_{i}-c_{i} (1+\lambda_{i} \theta_{i} )e_{i}^{p-1}=0$.Solving for $e_{i}^{*}$:

$e_{i}^{*}=[\frac{\rho_{i}(1-\theta_{i})+S_{i}\theta_{i} }{c_{i}(1+\lambda_{i}\theta_{i})}]^{1/p-1}$. Comparative statics with respect to $p$: When $p>2$ (more convex cost), the effort function is more sensitive to changes in $\theta_{i}$. The marginal cost of increasing the mandate is higher because effort drops more sharply. Consequently, the optimal mandate rates are lower for both types.

Optimal tiering under power cost: The architect's FOC is:

. Using $e_{i}^{*}=[A_{i} /B_{i} ]^{1/(p-1)}$, where $A_{i} =\rho_{i} (1-\theta_{i} )+S_{i} \theta_{i}$ and $B_{i}=c_{i}(1+\lambda_{i}\theta_{i})$: $\frac{\partial e_{i}^{*}}{\partial\theta_{i}}=\frac{1}{p-1}(\frac{A_{i} }{B_{i}})^{1/(p-1)-1}\frac{A'_{i}B_{i}-A_{i}B'_{i}}{B_{i}^{2}}$. For $p=3$, numerical evaluation yields $\theta_{D} \approx0.60$, $\theta_{I}\approx0.45$, differentiation 0.15.

General pattern: As pp increases, the optimal mandates converge toward the linear-quadratic case because the convexity makes effort more responsive, and the architect reduces mandates to avoid suppressing effort. The tiered advantage persists but is smaller.

**B.1.3 Fixed Cost of Compliance**

Model specification: $\Pi_{i} =\alpha_{i} (\gamma_{ii} Ki +\gamma_{ji }K_{j} )+\rho_{i} (1-\theta_{i} )e_{i }-c_{i} (1+\lambda_{i} \theta_{i} )e_{i} -F_{i} \cdot\lambda_{i} \theta_{i} \cdot1_{\{e_{i} >0\}}$,The fixed cost $F_{i}$ is incurred only if the project is active ($e_{i}>0$), capturing administrative, legal, and procedural overhead associated with open-source compliance. Optimal effort decision:

If $e_{i}>0$, the first-order condition is: $\rho_{i} (1-\theta_{i} )+S_{i }\theta_{i} -c_{i} e_{i} =0$, so: $e_{i}^{*}=\frac{\rho_{i} (1-\theta_{i} )+S_{i }\theta_{i}}{c_{i}}$.

The project remains active if the net payoff with $e_{i}^{*}>0$ exceeds the payoff of exiting (which is 0, assuming no fixed costs if inactive). This requires: $\rho_{i }(1-\theta_{i} )e_{i}^{*} +S_{i} \theta_{i} e_{i}^{*} -\frac{1}{2} c_{i} (e_{i}^{*} )^{2}-F_{i }\lambda_{i} \theta_{i }>0$.

Substituting $e_{i}^{*}$: $\frac{[\rho_{i} (1-\theta_{i} )+S_{i }\theta_{i}]^{2}}{2c_{i}}>F_{i }\lambda_{i} \theta_{i }$.

Effect on optimal tiering: The fixed cost $F$ penalizes participation. For independents, $F_{I}$ is relatively larger (as a fraction of their resources), so high $\theta_{I}$ would force them to exit. The architect sets $\theta_{I}$ lower to keep independents active.

For platforms, $F_{D}$ is relatively smaller, so the architect can impose higher $\theta_{D}$. This amplifies the tiering advantage.

In our numerical example, $F_{D} =0.1$ and $F_{I} =0.5$ (normalized), yielding $\theta_{D} \approx0.65$, $\theta_{I} \approx0.25$, differentiation 0.40, and health gain of 40%.

**B.2 Strategic Classification Gaming**

**B.2.1 Model Setup**

Let a project's true type be $i\in\{D,I\}$. The project can choose to report type $j\in\{D,I\}$ at a switching cost $s_{ij}$ (with $s_{ii}=0$). If it reports $j$, it faces mandate $\theta_{j}$.

There is a probability $q$ of being audited. If audited and found to have misreported, the project must pay a penalty $P$ and revert to its true mandate $\theta_{i }$.

**B.2.2 Expected Payoffs**

The expected payoff for a project of true type ii reporting $j$ is: $U_{i} (j)=(1-q)\Pi_{i} (\theta_{j })+q[\Pi_{i} (\theta_{i })-P]-s_{ij }$.The project reports truthfully ($j=i$) if $U_{i} (i)\geq U_{i} (j)$for all $j\neq i$.

**B.2.3 Incentive Compatibility Conditions**

For platforms ($i=D$) reporting as independents ($j=I$): Truthful reporting requires: $U_{D} (D)\geq U_{D} (I)$. Substituting: $\Pi_{D} (\theta_{D} )\geq(1-q)\Pi_{D} (\theta_{I} )+q[\Pi_{D} (\theta_{D} )-P]-s_{DI }$

Thus: $\Pi_{D} (\theta_{D} )-\Pi_{D} (\theta_{I} )\geq-\frac{s_{DI }+qP}{1-q}$.

Since $\Pi_{D} (\theta_{D} )<\Pi_{D} (\theta_{I} )$ (platforms prefer lower mandates), this condition is: $\Pi_{D} (\theta_{I} )-\Pi_{D} (\theta_{D}) \leq\frac{s_{DI }+qP}{1-q}$.

Interpretation: Misreporting is prevented if the gain from reporting as independent ($\Pi_{D} (\theta_{I} )-\Pi_{D} (\theta_{D})$) is less than the expected cost of doing so (switching cost plus expected penalty).

For independents ($i=I$) reporting as platforms ($j=D$), the analogous condition is: $\Pi_{I} (\theta_{D} )-\Pi_{I} (\theta_{I}) \leq\frac{s_{ID }+qP}{1-q}$.

But since $\theta_{D}>\theta_{I}$ and $\Pi_{I}$ is decreasing in $\theta$ (independents also prefer lower mandates), the left-hand side is negative, so this condition is automatically satisfied. Thus, the relevant binding constraint is the platform's incentive to misreport as independent.

**B.2.4 Constrained Optimal Policy**

The architect chooses $\theta_{D}$,$\theta_{I}$ to maximize $V_{A}$ subject to the incentive compatibility constraint: $\Pi_{D} (\theta_{I} )-\Pi_{D} (\theta_{D}) \leq\frac{s_{DI }+qP}{1-q}$.

This constraint limits how much differentiation is feasible. If the unconstrained optimal differentiation violates the constraint, the architect must reduce $\theta_{D}$ or increase $\theta_{I}$.

Optimal solution:

If the constraint binds, the optimal $\theta_{D}$ and $\theta_{I}$ satisfy: $\Pi_{D} (\theta_{I} )-\Pi_{D} (\theta_{D}) =\frac{s_{DI }+qP}{1-q}$, along with the architect's first-order conditions (Equation 13 in the main text).

The constrained optimal differentiation is smaller than the unconstrained optimum. The reduction in health gain relative to the unconstrained case is:

$\Delta V_{A}^{constraint }=\int_{0}^{\infty} e^{-rt}[H(\theta^{constraint})-H(\theta^{unconstraint})]dt<0$.

**B.2.5 Mitigation Strategies**

(1) Multi-dimensional classification: Instead of a single binary classification, use multiple criteria: revenue, employee count, funding sources, governance structure. This increases sijsij because it is harder to misrepresent along all dimensions simultaneously.

(2) Verification probability: Increase qq through regular audits and transparent reporting requirements. This directly raises the expected cost of misreporting.

(3) Penalty design: Set PP sufficiently high to deter misreporting. However, penalties must be credible and enforceable.

(4) Gradual mandate schedules: Instead of discrete $\theta_{D}$ and $\theta_{I}$, use a continuous or multi-tier schedule. This reduces the marginal gain from misreporting and thus the incentive to game the system.

**B.2.6 Equilibrium with Strategic Misreporting**

Define the effective mandate for a project of true type ii as:

$$\overrightarrow{\theta_{i}}=\left\{\begin{matrix} \theta_{i},if reporting truthfully \\ \theta_{j},if\text{ }reporting\text{ }as\text{ }type\text{ }j \end{matrix}\right.$$

with the reporting decision chosen to maximize expected payoff. The equilibrium features:

If $s_{DI}$ and $q$ are large enough, all projects report truthfully; If $s_{DI}$ is small and $q$ is low, platforms misreport as independents. Then the effective $\theta_{D}^{eff}=\theta_{I}$ (all platforms pay the lower rate), and the tiered policy collapses to a uniform policy in equilibrium.

The critical threshold for $s_{DI}$ is:$s_{DI}^{crit}=(1-q)[\Pi_{D} (\theta_{I} )-\Pi_{D} (\theta_{D} )]-qP$.

If $s_{DI}\geq s_{DI}^{crit}$, truthful reporting prevails. If $s_{DI}<s_{DI}^{crit}$, strategic misreporting occurs.

**B.3 Effort Reallocation (Core vs. Peripheral R&D)**

**B.3.1 Model Setup**

Introduce two types of R&D effort: Core R&D ($e_{i}^{c}$): Contributes to both open-source and proprietary outputs. Subject to the mandate.

Peripheral R&D ($e_{i}^{p}$): Contributes only to proprietary outputs. Not subject to the mandate.

Total effort: $e_{i}=e_{i}^{c}+e_{i}^{p}$. The open-source flow is $\eta_{i}\theta_{i}e_{i}^{c}$. Proprietary returns are $\rho_{i} [(1-\theta i_{ })e_{i}^{c} +e_{i}^{p}$. Costs are separable: $C_{i}(_{i}e_{i}^{c}+e_{i}^{p})=\frac{1}{2}c_{i}(e_{i}^{c})^{2}(1+\lambda_{i}\theta_{i})+\frac{1}{2}c_{i}(e_{i}^{p})^{2}$.

**B.3.2 Optimal Effort Allocation**

The followers' optimization problem is:

$\max_{e_{i}^{c},e_{i}^{p}} \left\{\alpha_{i} (\gamma_{ii} K_{i }+\gamma_{ji} K_{j} )+\rho_{i [}[(1-\theta_{i} )e_{i}^{c}+e_{i}^{p} ]-\frac{1}{2}c_{i}(e_{i}^{c})^{2}(1+\lambda_{i}\theta_{i})-\frac{1}{2}c_{i}(e_{i}^{p})^{2}+V_{i,K_{i }} \eta_{i} \theta_{i} e_{i}^{c} \right\}$.

First-order conditions: For $e_{i}^{c}$: $\rho_{i [}(1-\theta_{i} )+V_{i,K_{i }} \eta_{i} \theta_{i}-c_{i} (1+\lambda_{i}\theta_{i})e_{i}^{c} =0$. Thus: $e_{i}^{c*}=\frac{\rho_{i}(1-\theta_{i} )+V_{i,K_{i }} \eta_{i} \theta_{i}}{c_{i} (1+\lambda_{i}\theta_{i})}$

For $e_{i}^{p}$: $\rho_{i} -c_{i}e_{i}^{p} =0\Rightarrow e_{i}^{p*}=\frac{\rho_{i} }{c_{i}}$.

**B.3.3 Key Observations**

(1) Peripheral effort $e_{i}^{p*}$ is independent of the mandate rate $\theta_{i}$. Projects always do some proprietary R&D that is not subject to open-source requirements. (2) Core effort $e_{i}^{c}$ has the same functional form as the baseline model. Thus, the Laffer curve analysis from Section A.3 still applies: $Q_{i}(\theta_{i})=\eta_{i}\theta_{i}e_{i}^{c*}$ has an inverted-U s$Q_{i}(\theta_{i})=\eta_{i}\theta_{i}\frac{\rho_{i}(1-\theta_{i} )+S_{i}\theta_{i}}{c_{i} (1+\lambda_{i}\theta_{i})}$hape. (3) Total effort $e_{i}=e_{i}^{c}+e_{i}^{p}$ is higher than in the baseline model (where all effort was subject to the mandate). This means the mandate has a smaller proportional effect on total effort. (4) The optimal mandate is lower than in the baseline because projects can reallocate effort to peripheral R&D, making the mandate less effective at increasing total open-source output.

**B.3.4 The Laffer Curve**

Total open-source output from type $i$ is: $Q_{i}(\theta_{i})=\eta_{i}\theta_{i}\frac{\rho_{i}(1-\theta_{i} )+S_{i}\theta_{i}}{c_{i} (1+\lambda_{i}\theta_{i})}$. Maximize $Q_{i}$ with respect to $\theta_{i}$: $\frac{dQ_{i}}{d\theta_{i}}=\eta_{i}(\frac{A}{B}+\theta_{i}\frac{A'B-AB'}{B^{2}})=0$, where $A=\rho_{i}(1-\theta_{i} )+S_{i}\theta_{i}$ and . This yields exactly the same quadratic equation as in A.3. Thus, the revenue-maximizing mandate satisfies: $D_{i }\lambda_{i }\theta_{i}^{2} +2D_{i} \theta_{i} +\rho_{i} =0$, where $D_{i}=S_{i}-\rho_{i}$.

Implication: The reallocation does not change the revenue-maximizing mandate rate, but it reduces the total open-source output at that rate because $e_{i}=e_{i}^{c}+e_{i}^{p}$ and only $e_{i}^{c}$ contributes to $Q_{i}$. The Laffer curve is lower.

**B.4 Stochastic Shocks**

**B.4.1 Model Setup**

The state dynamics with Brownian shocks are:

$$\left\{\begin{matrix} dK_{D }=[\eta_{D} \theta_{D} e_{D} -\delta K_{D} ]dt+\sigma_{D} K_{D} dW_{D} \\ dK_{I }=[\eta_{I} \theta_{I} e_{I} -\delta K_{I} ]dt+\sigma_{I} K_{I} dW_{I} \end{matrix}\right.$$

where: $\sigma_{D}>0$ and $\sigma_{I}>0$ is the volatility of knowledge accumulation, $dW_{D}$ and $dW_{I}$ are Wiener processes with correlation , .

**B.4.2 HJB Equation for the Architect**

Using Itô's lemma, the architect's HJB equation is:

.

**B.4.3 First-Order Conditions**

The FOCs are the same as in the deterministic case because the variance terms do not depend on $\theta_{D}$ or $\theta_{I}$ (they depend only on $K_{D}$ and $K_{I}$):

..

Thus, the optimal $\theta_{i}^{*}$ is the same function of $V_{A,K_{I}}$ as in the deterministic case. However, $V_{A,K_{I}}$ now includes the variance terms, which affect the value functions through the cross-partial derivatives.

**B.4.4 Effect of Volatility on the Value Function**

From the HJB equation, an increase in $\sigma_{i}$ has two effects: (1) Precautionary effect: The variance terms $-\frac{1}{2}\sigma_{i}^{2}K_{i}^{2}V_{A,K_{i}K_{i}}$ appear (since $V_{A,K_{i}K_{i}}<0$ due to concavity). Higher $\sigma_{i}$ reduces the value of being in the ecosystem because uncertainty is costly. This tends to reduce the optimal mandate rates because the architect wants to protect the ecosystem from excessive volatility. (2) Option value effect: The correlation term can be positive or negative depending on and the sign of $V_{A,K_{D}K_{I}}$. If $V_{A,K_{D}K_{I}}>0$ (the health function is supermodular, meaning $K_{D}$ and $K_{I}$ are complements), then positive correlation increases the value of having both stocks.

**B.4.5 Correlation Effects**

(a) Uncorrelated shocks ():

The cross term vanishes. Higher volatility reduces $V_{A}$ and reduces the optimal mandates. The tiered advantage persists because the policy still targets heterogeneity in $\rho$ and $\lambda$.

(b) Perfectly correlated shocks ():

The variance term is $+\sigma_{D} \sigma_{I}K_{D}K_{I}V_{A,K_{D}K_{I}}+\frac{1}{2}\sigma_{D}^{2}K_{D}^{2} \frac{\partial^{2}V_{A}}{\partial K_{D}^{2}}V_{A,K_{D}K_{D}}+\frac{1}{2}\sigma_{I}^{2}K_{I}^{2} V_{A,K_{I}K_{I}}$. If $V_{A,K_{D}K_{I}}>0$ (complements), the cross term can partially offset the negative variance terms. The tiered and uniform regimes become more similar in their effect because the shocks move both stocks in the same direction. However, the tiered policy still contains the uniform policy as a special case, so it cannot be worse. The tiered advantage is attenuated, not reversed.

(c) Perfectly negatively correlated shocks ():

The cross term is negative, amplifying the negative effect of volatility. Tiered mandates become more valuable because they allow the architect to hedge: when one stock is hit, the other is boosted, and the differentiated mandates can respond to the state.

**B.5 Proprietary Knowledge Stock Extension**

**B.5.1 Model Setup**

Let proprietary knowledge stocks accumulate as:

,,

where $\delta_{P }$ is the depreciation rate of proprietary knowledge (which may differ from $\delta$, the depreciation of open-source knowledge). The followers' payoffs are:

$\Pi_{D}=\alpha_{D}\left[\gamma_{DD}K_{D} +\gamma_{ID}K_{I}\right]+\rho_{D}P_{D}-\frac{1}{2}c_{D}(1+\lambda_{D}\theta_{D}) e_{D}^{2}$, $\Pi_{I}=\alpha_{I}\left[\gamma_{II}K_{I} +\gamma_{DI}K_{D}\right]+\rho_{I}P_{I}-\frac{1}{2}c_{I}(1+\lambda_{I}\theta_{I}) e_{I}^{2}$.

**B.5.2 Optimal Effort Decisions**

The followers' value functions are now functions of four state variables: $V_{i }(K_{D} ,K_{I} ,P_{D} ,P_{I} )$. Due to linearity in $P_{D}$ and $P_{I}$, we can write:

$V_{D} =A_{D} K_{D} +B_{D} K_{I }+C_{D} P_{D} +D_{D} P_{I} +E_{D}$, $V_{I} =A_{I} K_{D} +B_{I }K_{I} +C_{I} P_{D} +D_{I} P_{I} +E_{I}$, where $A_{i}$, $B_{i}$, $C_{i}$, $D_{i}$, $E_{i}$are constants (or functions of $K_{D}$, $K_{I}$ in the more general case). Substituting into the HJB equations yields: For $e_{D}$:

. Thus:

. Similarly for $e_{I}$:

.

**B.5.3 Interpretation**

The effort now depends on both the value of open-source knowledge ($A_{D}$) and the value of proprietary knowledge ($C_{D}$). A higher mandate $\theta_{D}$: (1) Increases the weight on $A_{D}$ (open-source value). (2) Decreases the weight on $C_{D}$ (proprietary value). This creates an additional trade-off: effort is allocated between building open-source and proprietary knowledge stocks.

**B.5.4 Effect on Optimal Mandates**

The architect's FOC is still: , where . But now$e_{i}^{*}$ depends on $C_{i}$ (the marginal value of proprietary knowledge), which is endogenous.

Comparative statics: An increase in $\rho_{i}$ (private value) increases $C_{i}$ (the marginal value of proprietary stock), which reduces the optimal $\theta_{i}$ because more effort is diverted to proprietary R&D. An increase in $\alpha_{i}$ (ecosystem value) increases $A_{i}$ (the marginal value of open-source stock), which increases the optimal $\theta_{i}$.

The tiering result remains: $\theta_{D} >\theta_{I}$ if $\rho_{D}/\rho_{I}$ is sufficiently large and $\lambda_{D}/\lambda_{I}$ is sufficiently large. The magnitudes are slightly smaller than in the baseline because the proprietary stock channel adds an additional cost to mandates.

The tiered advantage persists, confirming that our core result is robust to the inclusion of proprietary knowledge dynamics.

**This completes the appendices. All proofs and derivations are consistent with the main text. The online supplementary material includes the complete code and additional numerical results.**
