# Supplementary material for: Can tiered open-source mandates optimize innovation? A differential game study osn platform vs. independent project dynamics in foundation software ecosystems
Source: PLoS One. 2026 Sep 11;21(9):e0340713. doi: 10.1371/journal.pone.0340713 (PMC13567796; doi:10.1371/journal.pone.0340713)
Supplement: Supplementary file 2 [file pone.0340713.s003.docx]

**Response to reviewers**

Dear Editor and Reviewers,

We sincerely thank the Editor and the two reviewers for their thoughtful, detailed, and constructive comments on our manuscript entitled "Can Tiered Open-Source Mandates Optimize Innovation? A Differential Game Study on Platform vs. Independent Project Dynamics in Foundation Software Ecosystems". We greatly appreciate the time and effort invested in reviewing our work.

We have carefully considered all comments and have revised the manuscript accordingly. Below we provide point-by-point responses to each concern raised by the reviewers. All modifications in the revised manuscript are highlighted in color (red) for easy identification. We believe the revisions have substantially strengthened both the theoretical rigor and the practical relevance of our study.

**Response to the Comments of Reviewer #1**

**Point 1:** The paper's central result — that heterogeneous agents benefit from differentiated policy treatment — is a well-established principle in mechanism design and price discrimination theory, and the authors do not adequately distinguish their contribution from this general result."

**Response 1:**

We thank the reviewer for this important observation. We fully agree that the general principle of optimal differentiation under heterogeneity is well-established in classical mechanism design (Wilson, 1993; Armstrong, 2006). However, our contribution is not to rediscover this general principle, but rather to apply it in a dynamic, strategic, and policy-endogenous context that has not been previously formalized. The novelty lies in three dimensions:

(1) Dynamic feedback policies rather than static schedules. Classical regulation theory derives static menus of contracts or tax schedules. Our model derives state-contingent feedback mandate functions that evolve with the knowledge stocks—a dimension absent in static mechanism design.

(2) Endogenous R&D investment and knowledge spillovers. Unlike price discrimination models where agents choose from a menu, our followers engage in strategic R&D competition with knowledge spillovers, creating non-convexities and dynamic externalities that fundamentally alter the optimal policy structure.

(3) Quantified boundary conditions. We identify specific thresholds (e.g.,$h>0.3$, $\lambda_{D} /\lambda_{I} >2.0$) and dynamic patterns (e.g., S-shaped health gains) that are unique to the open-source governance context and provide actionable guidance for ecosystem architects.

We have revised the Introduction (Section 1) and Section 2.4 to clarify these distinctions and position our contribution more precisely relative to the classical literature. The added text reads:

“While the general principle that heterogeneous agents warrant differentiated policy treatment is well-established in mechanism design and nonlinear pricing theory (Wilson, 1993; Armstrong, 2006), its application to dynamic open-source governance—where policy instruments interact endogenously with R&D investment over time and where knowledge spillovers create non-convexities—remains unformalized. Our contribution is not to rediscover that differentiation helps, but to derive state-contingent feedback mandate functions in a dynamic setting, to quantify how network effect strength and spillover asymmetry modulate the optimal degree of differentiation, and to provide testable boundary conditions that are specific to the governance of AI infrastructure ecosystems."

**Point 2:** The claimed 15–40% welfare gains and specific optimal thresholds ($\theta_{D}=60-75\%$, $\theta_{I}\approx30-40\%$) are artifacts of the authors' parameter calibration, which lacks empirical grounding. Section 5.1 claims calibration is 'based on economic theory, empirical software engineering studies, and the strategic characteristics of platform vs. independent projects,' but no citations are provided for any specific numerical value.

**Response 2:**

The reviewer raises a valid and important concern. We have substantially revised Section 5.1 to provide empirical citations for each key parameter. Specifically:

For R&D efficiency differentials ($\lambda_{D}$, $\lambda_{I}$), we now cite Ghosh et al. (2002) and Lerner & Tirole (2005) on productivity differences between corporate and volunteer contributors.

For compliance costs ($\eta_{D}$, $\eta_{I}$), we cite the Open Source Security Foundation (2023) annual survey on enterprise compliance costs and legal overhead.

For knowledge depreciation ($\delta=0.15$), we cite Robles & González-Barahona (2010) on software project lifecycle decay.

For spillover asymmetry ($\gamma_{DI }>\gamma I_{D}$), we cite Nagappan et al. (2013) on code reuse patterns across projects.

We have also added a clear caveat in Section 5.1:

“These calibration values are intended to reflect typical magnitudes in AI foundation software ecosystems, not precise estimates. The core conclusions are robust to parameter variations within reasonable ranges, as demonstrated in the Monte Carlo analysis in Section 6.7.”

Importantly, we have reframed our contribution away from claiming precise numerical thresholds as universal constants and toward emphasizing the structural conditions under which tiered mandates dominate. We explicitly state that the thresholds are "illustrative reference points, not universal constants" and note that "in real ecosystems where multiple parameters shift jointly, the relevant threshold will differ" (Section 6.3.1).

**Point 3:** Equation 12 contains a notation error. The FOCs for the tiered case show ∂K̇_I/∂θ twice, but these should be ∂K̇_D/∂θ_D = 0 and ∂K̇_I/∂θ_I = 0.

**Response 3:**

We apologize for this typographical error. We have corrected Equation (12) and the surrounding text in Section 4.2.2 to read:

“Taking the first-order conditions with respect to each control variable yields a system of two equations:

 (12)”

We have also verified that all subsequent derivations in the numerical analysis are consistent with these corrected FOCs.

**Point 4:** The 'AI software ecosystem' framing adds little. The model contains no AI-specific mechanism — no training data dynamics, no model capability curves, no compute scaling laws. The model applies equally to any two-type open-source ecosystem. The authors should either incorporate AI-specific structure or generalize the framing honestly.

**Response4:**

We agree with the reviewer that the model is generalizable to any two-type open-source ecosystem. However, we chose to frame the study in the context of AI foundation software stacks because this is where the governance challenge is most urgent and policy-relevant. To address the reviewer's concern, we have:

Generalized the title from "AI Software" to "Foundation Software Ecosystems" while retaining the AI motivation in the introduction.

“Can Tiered Open-Source Mandates Optimize Innovation? A Differential Game Study on Platform vs. Independent Project Dynamics in Foundation Software Ecosystems”

Added a paragraph in Section 1 that explicitly articulates the AI-specific features that motivate our model:

“The AI software stack exhibits distinctive features that differentiate it from general-purpose software ecosystems: extreme network effects where each additional compatible tool exponentially increases platform value; deep hardware-software coupling where frameworks are optimized for specific accelerators; and geopolitical salience where control over the stack confers strategic advantage. These features amplify the governance challenges we analyze and motivate our focus on this specific context.”

Added a limitation in Section 7.3 acknowledging the model's generality:

“While our model is specified for AI foundation ecosystems, its structure applies to any two-type open-source ecosystem. Future work could incorporate AI-specific production functions (e.g., compute-data-model complementarities), but the present framework intentionally adopts a parsimonious structure to maintain analytical tractability as the first dynamic game model of tiered open-source governance.”

**Point 5**: The paper is substantially longer than it needs to be. The robustness section (Section 6) runs through alternative health functions, cost functions, stochastic extensions, gaming, and boundary conditions — much of which confirms what theory already predicts (differentiation helps more when agents differ more, less when they're similar). This could be cut by 40% without losing substance.

**Response5:**

We appreciate this constructive criticism. We have substantially streamlined Section 6 by:

Moving detailed derivations of alternative cost functions (6.1.2), strategic classification gaming (6.2), and stochastic shocks (6.4) to Appendices B.1–B.3, retaining only the key mechanisms and findings in the main text.

Condensing the text in each robustness sub-section to one to two paragraphs summarizing the core insight, with pointers to the Appendix for readers interested in full derivations.

Removing redundant discussions where results merely confirm theoretical predictions, and focusing instead on cases where the robustness checks reveal nuanced or non-obvious findings (e.g., the Min-Max health function producing wider differentiation, which we explain in Section 6.1.1).

We estimate that Section 6 has been reduced by approximately 35–40% in length while preserving all substantive findings.

**Point 6**: Table 5.1 mislabels the 'Weak Network Effects' row: the Optimal Policy column shows π_D ≈ 0.5–0.6 (private value notation) instead of θ_D (mandate notation)."

**Response6:**

We thank the reviewer for catching this error. We have corrected Table 5.1 to consistently use $\theta_{D}$ and $\theta_{I}$ for mandate rates across all rows.

“Based on our analysis, we identify three archetypal ecosystem configurations with corresponding optimal policy prescriptions:

**Table 5.1: Policy Scenarios and Recommendations**

| Scenario | Characteristics | Optimal Policy | Expected Welfare Gain |
| --- | --- | --- | --- |
| Asymmetric Compliance | $\lambda_{D} \gg\lambda_{I}$, $\rho_{D} \gg\rho_{I}$ | Strongly tiered: $\theta_{D}\approx0.70$; $\theta_{I}\approx0.30$ | 25–40% |
| High Platform Monetization | $\rho_{D} \gg\rho_{I}$, moderate $\lambda$ | Moderately tiered: $\theta_{D}\approx0.65$; $\theta_{I}\approx0.35$ | 15–25% |
| Weak Network Effects | $\gamma_{DI}$ low, $\gamma_{ID}$ low, | Mild differentiation or uniform: $\theta_{D}\approx0.55$; $\theta_{I}\approx0.45$ | 0–10% |
| Balanced Ecosystem | Moderate asymmetry across all dimensions | Calibrated tiered: $\theta_{D}\approx0.60$; $\theta_{I}\approx0.40$ | 10–20% |

"

**Point 7:** The diversity term in the ecosystem health function (Eq. 5) uses √(K_D · K_I), which is undefined when either stock is zero. The authors should discuss how the numerical algorithm handles boundary cases.

**Response7:**

We have added a note in Section 3.3.2 and Section 4.3 explaining our boundary treatment:

In numerical implementation, we use a regularized version of the geometric mean term, $\sqrt{K_{D} (t)K_{I} (t)+\varepsilon}$ with $\varepsilon=10^{-6}$, to avoid boundary singularities when either stock approaches zero.

“The quadratic term $-\mu(K_{D} (t)+K_{I} (t))^{2}$ directly penalizes imbalance between the two knowledge stocks, capturing the architect’s preference for a pluralistic ecosystem where neither type dominates excessively. In numerical implementation, we use a regularized version of the geometric mean term, $\sqrt{K_{D} (t)K_{I} (t)+\varepsilon}$ with $\varepsilon=10^{-6}$, to avoid boundary singularities when either stock approaches zero.”

We have also added a discussion of boundary handling in the numerical algorithm description (Section 4.3).

**Point 8:** Figure 6.4's legend labels ('data1,' 'data2,' 'data3') are placeholder text that was never replaced."

**Response8:**

We apologize for this oversight. All figure legends and labels have been corrected to their proper designations (e.g., 'Uniform,' 'Tiered,' 'Health Gain') in the revised manuscript and figures.

**Point 9:** Several references appear incomplete or contain formatting errors (e.g., 'P´erez, 2005' in the text appears as 'P´erez, 2021' in the reference list; 'Almeida et al., 2019;' has a trailing semicolon followed by a closing parenthesis with no second reference).

**Response9:**

We have thoroughly reviewed and corrected all references. The Pérez citation has been unified to Pérez (2021) throughout. The Almeida et al. citation has been corrected. We have also added missing references (including Ghosh et al., 2002; Lerner & Tirole, 2005; Armstrong, 2006; Wilson, 1993; Mirrlees, 1971; Mussa & Rosen, 1978; Kalliamvakou et al., 2016; Robles et al., 2017; Open Source Security Foundation, 2023) and verified that all cited works appear in the reference list and all listed references are cited in the text.

**Point 10:** The stochastic extension (Section 6.4) reports that under tiered mandates, K_I = 2.40 ± 1.60 versus 3.38 ± 2.27 under uniform. The independent project's mean knowledge stock is lower under tiered mandates, which contradicts the deterministic result in Section 5.2.2 (+50% gain). This discrepancy is not discussed.

**Response10:** We thank you for this constructive suggestion. We fully agree that we should articulate the theoretical gap more clearly, with an explicit comparison to existing studies. Based on your suggestion, we have added the following discussion:

“This apparent reduction in independent mean knowledge under tiered mandates in the stochastic simulation reflects a different equilibrium concept: under uncertainty, independent projects may optimally reduce effort in response to increased platform knowledge volatility (a precautionary effect). The deterministic result (+50%) represents the steady-state comparison under no uncertainty; the stochastic result incorporates option-value effects. The key insight is that tiered mandates increase platform knowledge even under uncertainty, while the independent effect is sensitive to the correlation structure of shocks. This highlights that stochastic robustness is conditional—not universal—and should be interpreted as a qualification, not a contradiction, of the deterministic benchmark.”

**Point 11:** The paper would benefit from a brief discussion of how the binary platform/independent classification maps to real ecosystems where projects exist on a spectrum (e.g., Hugging Face, which is VC-backed but community-driven).

**Response11:**

We thank you for this important observation. We have added a discussion in Section 3.1:

“We acknowledge that the binary classification of projects as 'platform' versus 'independent' is a simplification. In reality, projects such as Hugging Face occupy intermediate positions, combining venture capital backing with strong community engagement. Our binary model can be understood as the limiting case of a continuum where the two types represent poles of the distribution. Extending the model to a continuous type space is a promising direction for future work (see Section 7.3); however, the binary formulation allows us to derive sharp analytical insights about the structural conditions under which differentiation is most valuable.”

We have also added this as a limitation in Section 7.3.

**Point 12:** For these reasons I recommend this paper be revised before publication. The model construction is competent and the analysis is thorough, but the core contribution needs sharper positioning relative to existing theory on optimal differentiation, and the parameter calibration requires empirical grounding before the claimed thresholds can be presented as policy-relevant.

**Response11:**

We are grateful for the reviewer's balanced and constructive assessment. We have addressed all concerns as detailed above and believe the revised manuscript now presents a more sharply positioned contribution with empirically grounded calibration and appropriately qualified policy recommendations.

**Response to the Comments of Reviewer #2**

**Point 1:** A foundational logical flaw runs through the design of the follower payoff functions, as they effectively double-count the value of internally developed knowledge. Each firm’s own open-source knowledge stock is included as a component of its ecosystem benefit, treating knowledge the firm itself has created and released as if it delivers incremental external value back to the original developer. This makes little economic sense: the costs of developing that knowledge are already fully accounted for in the R&D cost term, and any private returns from undisclosed portions of the code are calculated separately. Adding one’s own contributed knowledge back as an ecosystem benefit artificially inflates perceived payoffs without any defensible theoretical justification.

**Response1:**

We sincerely thank the reviewer for identifying this critical issue. We have fundamentally revised the follower payoff functions to eliminate the double-counting problem.

Original formulation (flawed):

$$\max_{e_{D}(t)\geq0} J_{D}=\int_{0}^{\infty} e^{-\rho t}\left\{\alpha_{D}\left[K_{D} (t)+\gamma_{ID}K_{I} (t)\right]+\rho_{D}(1-\theta_{D}) e_{D} (t)-\frac{1}{2}c_{D}(1+\lambda_{D}\theta_{D}) e_{D} (t)^{2}\right\}d$$

Revised formulation (corrected):

$$\max_{e_{D}(t)\geq0} J_{D}=\int_{0}^{\infty} e^{-\rho t}\left\{\alpha_{D}\left[\gamma_{DD}K_{D} (t)+\gamma_{ID}K_{I} (t)\right]+\rho_{D}(1-\theta_{D}) e_{D} (t)-\frac{1}{2}c_{D}(1+\lambda_{D}\theta_{D}) e_{D} (t)^{2}\right\}dt$$

“where $\gamma_{DD}<1$ represents the residual benefit the platform derives from its own past contributions (e.g., reputation, internal reuse efficiency), and $\gamma_{ID}$ represents the spillover from the independent project's knowledge. This formulation avoids the double-counting concern whereby a firm would otherwise obtain credit for knowledge it had already paid to produce. $\alpha_{D}$i s the marginal value of ecosystem knowledge to $D$, $\gamma_{ID}$ is the spillover coefficient from $I$ to $D$, $\rho_{D}$ is the marginal private value of proprietary code, $c_{D}$ is the base R&D cost coefficient, and $\lambda_{D}$ scales the additional cost burden of the mandate.”

Similarly for the independent project:

$$\max_{e_{I}(t)\geq0} J_{I}=\int_{0}^{\infty} e^{-\rho t}\left\{\alpha_{I}\left[\gamma_{II}K_{I} (t)+\lambda_{DI}K_{D} (t)\right]+\rho_{I}(1-\theta_{I})\alpha_{I} e_{I} (t)-\frac{1}{2}c_{I}(1+\lambda_{I}\theta_{I}) e_{I} (t)^{2}\right\}dt$$

with $\gamma_{II}<1$.

**Point 2:** The ecosystem health function suffers from a fundamental mismatch between its stated purpose and its actual mathematical structure. The quadratic term described as a penalty for imbalance between the two developer types in fact measures only the squared total of all accumulated knowledge, which captures diminishing returns to scale rather than any measure of relative disparity. A genuine penalty for structural imbalance would track the gap between the two knowledge stocks, rewarding parity and punishing extreme dominance by either side. As currently specified, the term conflates pure scale effects with distributional effects, attaching a misleading conceptual label to a mechanism that operates in an entirely different dimension.

**Response2:**

We thank you for this important and constructive comment. We have revised the health function to use a genuine imbalance penalty:

Original (flawed):

$$H(K_{D} (t),K_{I} (t))=\alpha(K_{D} (t)+K_{I} (t))+\beta\sqrt{K_{D} (t)K_{I} (t)}-\mu(K_{D} (t)+K_{I} (t))^{2}$$

Revised (corrected):

$$H(K_{D} (t),K_{I} (t))=\alpha(K_{D} (t)+K_{I} (t))+\beta\sqrt{K_{D} (t)K_{I} (t)}-\mu(K_{D} (t)-K_{I} (t))^{2}$$

The quadratic term now directly penalizes the gap between the two knowledge stocks, capturing the architect's preference for a balanced, pluralistic ecosystem. This is explicitly stated in Section 3.3.2:

"The quadratic term$-\mu(K_{D} (t)-K_{I} (t))^{2}$ directly penalizes imbalance between the two knowledge stocks, capturing the architect's preference for a pluralistic ecosystem where neither type dominates excessively."

All numerical simulations in the revised manuscript have been re-run with this corrected health function.

**Point 3:** For a framework built explicitly to capture long-run evolutionary dynamics, the model treats proprietary knowledge with surprising staticity. Only open-source knowledge accumulates as a persistent stock over time; proprietary code is modeled purely as a one-time flow of revenue that vanishes in the same period it is created. In practice, private R&D investments build durable internal knowledge capital that improves future R&D productivity and shapes long-term competitive advantage. By reducing proprietary development to a simple contemporaneous trade-off between cost and immediate revenue, the model abandons the very dynamic logic that justifies the use of a differential game in the first place.

**Response3:**

We agree that this is a limitation of the baseline model. We have added an extension in Appendix B.5 where proprietary knowledge accumulates as a persistent stock:

with payoffs:

$$\Pi_{D} =\alpha_{D} (\gamma_{DD} K_{D} +\gamma_{ID} K_{I} )+\rho_{D} P_{D} -\frac{1}{2} c_{D} (1+\lambda_{D} \theta_{D} )e_{D}^{2}$$

$$\Pi_{I} =\alpha_{I} (\gamma_{II} K_{I} +\gamma_{DI} K_{D} )+\rho_{I} P_{I} -\frac{1}{2} c_{I} (1+\lambda_{I} \theta_{I} )e_{I}^{2}$$

Numerical results from this extension (reported in Appendix B.5) confirm that the tiered advantage persists, with slightly reduced magnitudes (e.g., health gain of 28% vs. 32% in the baseline). The qualitative conclusion—that tiered mandates outperform uniform mandates under heterogeneity—is unchanged.

We have also added a footnote in Section 3.3.1 directing readers to Appendix B.5.

**Point 4:** The core rationale for tiered regulation remains theoretically underspecified, because the defining difference between platform and independent projects is never clearly isolated. The two types differ across half a dozen structural parameters simultaneously, from R&D efficiency and base operating costs to compliance burdens, monetization capacity, ecosystem dependence, and spillover sensitivity. No single dimension is identified as the essential trait of “platformness” that justifies differential treatment. This leaves the policy argument for tiering fundamentally ambiguous: readers cannot tell which source of heterogeneity drives the reported benefits, nor why tiering along the platform–independent axis is preferable to classification along any other observable dimension.

**Response4:**

We thank you for this important and constructive comment. We have added a clear definition of "platformness" in Section 3.1:

"We define 'platformness' (the essential trait distinguishing $D$ from $I$) as the combination of (i) higher private appropriation capacity ($\rho_{D}>\rho_{I}$), and (ii) higher compliance cost elasticity ($\lambda_{D}>\lambda_{I}$). These two features are conceptually linked: a platform's larger scale and deeper commercial integration simultaneously create greater monetization opportunities and higher organizational friction in open-sourcing code. The other parameter differences ($\beta$, $\eta$, $\gamma$) are secondary consequences or moderators, not defining traits. We conduct counterfactual analysis in Section 6.3.1 where only the defining traits vary and others are held equal, confirming that these two dimensions are the primary drivers of the tiering advantage."

**Point 5**: One of the paper’s central claims about tiered policy design rests on a basic misreading of the optimization structure. The paper asserts that under a tiered regime, the regulator can set each mandate rate independently to maximize the growth of its corresponding knowledge stock. This decoupling is mathematically impossible, because the ecosystem health function includes strong interaction effects between the two knowledge stocks. The marginal value of adding to one stock depends directly on the current size of the other, so the optimal rate for platform projects and the optimal rate for independent projects are jointly determined in a single coupled optimization problem. Treating them as separate, independent decisions violates the basic logic of optimal control in interconnected systems.

**Response5:**

We thank you for this important and constructive comment. We have revised the relevant passage in Section 4.2.2 to eliminate the claim of "decoupling":

"Strictly speaking, the two FOCs remain coupled through the value function $V_{A}(K_{D},K_{I})$ and its cross-partial derivatives. The statement that the architect “independently tunes” each mandate is a heuristic for the fact that the tiered regime has two control variables rather than one, allowing the planner to move in two directions in the policy space. The full feedback solution, obtained numerically, accounts for the coupling through the joint value function. We do not claim analytical separability."

**Point 6**: Formal errors in the derivation of the tiered optimum further compromise the analytical integrity of the paper. The first-order conditions presented for the tiered policy regime incorrectly retain notation from the uniform policy scenario and fail to distinguish derivatives with respect to the two separate mandate variables. This is not merely a typographical oversight: it means the algebraic step that is supposed to characterize the optimal tiered structure is formally mis-specified, and the conclusions drawn from that step cannot be taken as valid outputs of the model as written.

**Response6:**

We thank you for this important and constructive comment. We apologize for this error. We have corrected the FOCs in Section 4.2.2:

All subsequent derivations have been verified for consistency with these corrected FOCs.

**Point 7**: Several key propositions are presented as established results without the supporting derivation needed to validate them. For instance, the claim that mandate rates have an ambiguous net effect on R&D effort is stated as a general principle but never formally derived through comparative static analysis. Similarly, the discussion of strategic classification gaming posits threshold values for switching costs and verification accuracy without explicitly solving for those thresholds or proving that a stable equilibrium exists at those values. These assertions read as intuitive conjectures rather than rigorously proven results, weakening the logical chain that connects the model’s assumptions to its conclusions.

**Response7:**

We thank you for this important and constructive comment. We have added full derivations for all propositions in Appendix A:

Proposition 4.1 (comparative statics of follower effort): Full derivation of $\partial e_{i}^{*}/\partial\theta_{i}$with interpretation of the ambiguous sign (Appendix A.1).

Proposition 4.0 (existence and uniqueness): Proof following the contraction mapping argument in Dockner et al. (2000), with assumptions explicitly stated (Appendix A.2).

Proposition 4.2 (optimal mandate structure): Detailed derivation of the quadratic equation for $\theta_{i}^{*}$ and comparison of $\theta_{D}^{*}$ and $\theta_{I}^{*}$ (Appendix A.3).

Classification gaming thresholds (Section 6.2): Formal derivation of the incentive compatibility constraint and equilibrium conditions (Appendix B.2).

**Point 8**: The extension to stochastic shocks includes a conclusion that contradicts a basic principle of policy instrument design. The paper suggests that when external shocks are perfectly correlated across the two project types, uniform mandates may become preferable due to unspecified risk-sharing benefits. This reasoning is unsound because a tiered policy framework strictly contains the uniform policy as a special case where both rates are set equal. A broader set of policy tools cannot produce a worse optimal outcome than a constrained subset of those same tools. There is no mechanism in the model that would generate a risk-sharing advantage for uniform rules, and the claim runs counter to fundamental optimization logic.

**Response8:**

We thank you for this important and constructive comment. We have deleted this erroneous claim and revised Section 6.4 to read:

"When shocks are perfectly correlated (), the variance of the health function under tiered and uniform regimes converges, and the tiered advantage is attenuated but does not reverse. We find no theoretical or numerical case in which tiered mandates are strictly dominated, consistent with the fact that tiered policies contain uniform policies as a special case. However, the magnitude of the tiered advantage shrinks as correlation increases, because the two knowledge stocks move in tandem and the diversification value of differentiated governance diminishes."

**Point 9**: The numerical analysis fails to decompose the channels through which tiered mandates produce their observed effects, leaving causal claims under-supported. Simulations show that tiered rules increase the platform’s knowledge stock while also slightly raising its R&D effort, but the paper never separates the direct mechanical effect of a higher contribution requirement from the indirect incentive effect operating through changed effort levels. Given the platform’s heavier compliance burden, basic incentive logic would predict that a stricter mandate should suppress effort; any observed increase must therefore be driven by feedback from a larger shared knowledge pool. The paper does not delineate the conditions under which this feedback effect dominates or quantify its strength, instead attributing the outcome vaguely to better incentive design without closing the causal loop.

**Response9:**

We thank you for this important and constructive comment. We have added a channel decomposition analysis in Section 5.2.4 (newly added):

"**5.2.4 Channel Decomposition**

To understand the mechanisms driving these results, we decompose the knowledge gains under the tiered mandate into three channels:

(1) Direct contribution effect: The increase in open-source flow holding effort constant at uniform-mandate levels.
(2) Effort response effect: The additional contribution from changes in equilibrium R&D effort.
(3) Spillover effect: The contribution from increased knowledge of the other type.

The decomposition reveals that for the platform project, the direct contribution effect accounts for approximately 65% of the knowledge gain, while the effort response effect contributes 20% (the platform increases effort despite higher mandates due to the larger commons), and the spillover from the independent project contributes 15%. For the independent project, the spillover from the platform is the dominant channel (55%), with direct contribution (25%) and own effort response (20%) playing secondary roles. This asymmetry in channels justifies the tiered design: high platform mandates fuel the commons, which in turn drives independent innovation.”

**Point 10:** TThe boundary conditions presented as general policy guidelines are derived from highly restrictive single-parameter sensitivity tests, and their real-world generality is therefore overstated. Thresholds such as the required ratio of compliance costs between platform and independent projects are calculated by varying one parameter at a time while holding all others fixed at baseline values. This local comparative static exercise cannot support the paper’s tendency to present these thresholds as universal rules of thumb. When multiple parameters shift simultaneously, as they routinely do in real ecosystems, the critical thresholds will almost certainly shift as well, and the analysis provides no guidance on how or by how much.

**Response 10:**

We thank you for this important and constructive comment. We agree with the reviewer that thresholds derived from single-parameter sensitivity analyses should not be presented as universal constants. To address this concern, we have added a caveat paragraph at the end of Section 6.3 (and cross-referenced in Section 6.6) explicitly clarifying that all reported thresholds are illustrative reference points derived from one-parameter-at-a-time perturbations at baseline values. We emphasize that in real ecosystems—where multiple parameters shift simultaneously—the relevant thresholds will differ, and we direct readers to the adaptive governance framework (Section 6.6) which is designed to address this very limitation by emphasizing continuous recalibration rather than fixed cutoffs. The newly added text reads:

"A caveat is in order regarding the interpretation of the boundary conditions reported in this section. Unless otherwise noted, all thresholds are derived from one-parameter-at-a-time sensitivity analyses conducted at baseline parameter values. They are intended as illustrative reference points rather than universal constants. In real-world ecosystems where multiple structural parameters shift jointly—for example, when compliance cost asymmetry and monetization gap increase simultaneously—the relevant thresholds will shift accordingly. The adaptive governance framework proposed in Section 6.6 is designed precisely to address this limitation: rather than prescribing fixed numerical cutoffs, it emphasizes continuous monitoring and recalibration of mandate rates as ecosystem conditions evolve. Policymakers should therefore treat the specific threshold values (e.g., $h>0.3$, $\lambda_{D} /\lambda_{I} >2.0$) as diagnostic heuristics rather than rigid policy rules, and complement them with ecosystem-specific empirical assessment."

We believe this revision appropriately qualifies our policy recommendations while preserving their practical value as diagnostic heuristics..

**Point 11:** Counterintuitive findings in the robustness analysis are left without adequate theoretical explanation. For example, the health function specification that embodies the strongest aversion to structural imbalance produces the widest gap between the two mandate rates. At first glance this seems paradoxical: greater concern for balance would intuitively call for more similar treatment, not more divergent rules. The paper brushes this result aside with a generic reference to complementarity effects but does not unpack the mechanism that would produce this pattern. Without a clearer account of why the result emerges, the robustness exercise confirms numerical sensitivity but does not deepen theoretical understanding.

**Response 11:**

We thank you for this important and constructive comment. We have added a brief explanation in Section 6.1.1 (with full derivation in Appendix B.1) clarifying that the Min-Max result follows from a Ramsey-type logic: the planner raises platform contributions to lift the minimum knowledge stock while protecting independents with a low mandate.

**Point 12:** The paper systematically stretches the concept of welfare in a way that inflates the apparent significance of its findings. Improvements in the ecosystem health metric are consistently described as welfare gains in the range of 15 to 40 percent, implying broad social benefits. Yet the model includes no representation of end users, consumer surplus, or broader societal outcomes; it captures only the objectives of the ecosystem architect and the two developer types. What the paper calls welfare is in fact the architect’s narrow objective function, and equating it to aggregate social welfare confuses a partial governance metric with a comprehensive welfare measure.

**Response 12:**

We thank you for this important and constructive comment. The reviewer raises a valid point. We have made a comprehensive terminological revision throughout the manuscript:

(1) All instances of "welfare" and "welfare gains" have been replaced with "ecosystem health" and "health improvements" to make clear that we are referring specifically to the architect's composite health function, not a full measure of social welfare.
(2) We have added an explicit clarification in the Abstract:

“Throughout this paper, 'health improvement' refers specifically to the ecosystem architect's composite health function $H(K_{D} ,K_{I })$, a governance metric of scale, diversity, and balance, not a full measure of social welfare."

We believe these revisions appropriately address the concern and prevent any misinterpretation of our findings as claiming broad social welfare gains.

**Point 13:** The paper’s claim to novel theoretical contribution is weakened by its lack of engagement with established literatures on differentiated regulation. The study positions itself as the first to endogenize tiered open-source mandates, but it does not connect its argument to the long-standing bodies of research on optimal taxation, non-linear pricing, and type screening that have analyzed policy design for heterogeneous agents for decades. It does not explain what distinctive insight the differential game setting adds beyond what classical regulation theory already provides, leaving readers uncertain about the exact nature and scope of its theoretical advance.

**Response 13:**

We thank you for this important and constructive comment. We have added a new paragraph in Section 2.4 explicitly connecting our work to the classical regulation literature:

"Our approach is related to, but distinct from, the classical literature on optimal taxation and nonlinear pricing (Mirrlees, 1971; Mussa & Rosen, 1978; Armstrong, 2006). In those models, a principal designs a menu of contracts or tax schedules to screen a continuum of agent types in a static or repeated setting. Our contribution differs in three ways: (i) the policy instrument is a dynamic feedback rule rather than a static schedule; (ii) agents' types are exogenously fixed and observable (platform vs. independent) rather than screened; and (iii) the key friction is knowledge spillovers and strategic R&D interaction, not private information. This makes our model complementary to, rather than a subset of, classical regulation theory. We explicitly treat the choice between uniform and differentiated rules as a policy design problem in a dynamic commons, a setting that has received limited attention in the regulation literature."

**Point 14:** Foundational questions about equilibrium validity remain unaddressed throughout the analysis. The paper never explicitly specifies whether it is solving for open-loop or closed-loop feedback equilibria, and it provides no formal proof that an equilibrium exists or that it is unique. The numerical policy iteration algorithm is described step by step, but its convergence properties are not established theoretically. Without such guarantees, there is no way to rule out the possibility of multiple equilibria, unstable dynamics, or algorithmic non-convergence, all of which would cast doubt on the reliability of the reported numerical results.

**Response 14:**

We thank you for this important and constructive comment. We have added Proposition 4.0 at the beginning of Section 4:

“**Proposition 4.0** (Existence and Uniqueness). Under Assumptions 1–4 and the parameter restrictions specified in Appendix A.2, the system of HJB equations (7)–(11) admits a unique viscosity solution in the class of continuously differentiable functions on the compact state space $S=[0,\overline{K}]^{2}$. The feedback strategies derived from the first-order conditions constitute a unique Markov-perfect equilibrium. Proof is provided in Appendix A.2, following the contraction mapping argument in Dockner et al. (2000, Chapter 7).”

We have also explicitly stated that we are solving for closed-loop feedback equilibria (Markov-perfect equilibria) in Section 3.4 and Section 4.3. The numerical Policy Iteration Algorithm (PIA) convergence properties are discussed in Section 4.3 and Appendix A.2.

**Point 15:** There is a persistent logical gap between the model’s theoretical constructs and the practical realities of policy implementation. The core state variable driving the entire framework—effective open-source knowledge stock—is not directly measurable in real-world ecosystems. Similarly, clear, verifiable criteria for classifying projects and auditing compliance are never operationalized. Even so, the paper’s proposed adaptive governance framework calls for continuous parameter monitoring and dynamic recalibration of mandate rates. It offers no account of how policymakers would actually measure these unobservable quantities in practice, creating a rift between the model’s elegant theoretical structure and its feasibility as a real-world governance tool.

**Response 15:**

We thank you for this important and constructive comment. We have added a detailed discussion in Section 6.5.1 (newly added):

“We acknowledge that the core state variable—effective open-source knowledge stock—is not directly observable in its theoretical purity. In practice, we propose proxies: for platforms, this could be measured by the number of active contributors to core repositories weighted by commit frequency and code review acceptance; for independents, by the same metrics plus community responsiveness indicators. The classification between platform and independent projects can be operationalized using thresholds on annual revenue, employee count, and corporate governance structure (e.g., board composition). These proxies are imperfect but feasible, and are commonly used in empirical OSS studies (Robles et al., 2017; Kalliamvakou et al., 2016). We do not suggest that the theoretical model maps directly into administrative rules; rather, the model provides a conceptual benchmark and directional guidance for calibrated governance."

We have also added this as a limitation in Section 7.3.

**Point 16:** The paper systematically overstates the novelty of its contribution.

**Response 16:**

We thank you for this important and constructive comment. We have carefully revised the Abstract, Introduction, and Conclusion to more modestly and precisely frame our contributions. We now emphasize that our contribution is the application of the general principle of optimal differentiation to a dynamic, open-source governance context with specific, quantifiable boundary conditions, rather than claiming the general principle itself as novel. The revised framing is more accurate and appropriately modest.

**Additional Changes per Editorial Requests**

(1) **Data Availability Statement**: We have supplemented a complete Data Availability Statement that fully complies with the policy requirements of PLOS ONE. Consistent with the actual situation of our manuscript, we declare that: "All minimal data points required to replicate the study findings, including the parameter calibration values, statistical measures and graph construction datasets, are fully available within the manuscript in Section “5.1 Parameter Calibration and Justification”.
(2) **Author Contributions**: We have revised the Author Contributions section to use the CRediT (Contributor Roles Taxonomy) format:

**“Author contributions**

**Bingyang Wu** (First Author): Conceptualization; Data curation; Formal analysis; Investigation; Methodology; Validation; Visualization; Writing – original draft; Writing – review & editing.

**Yongxi Yi** (Corresponding Author, *marked with *): Conceptualization; Funding acquisition; Project administration; Resources; Supervision; Writing – review & editing.

All authors jointly reviewed and approved the final version of the manuscript, confirming the accuracy and integrity of the work.”

(3) **Author and author instructions:** We have carefully reviewed the author instructions, added the complete list of all authors and their corresponding affiliations on the title page, and clearly marked the corresponding author with an asterisk, which is consistent with the information filled in the online submission system.
(4) **Ethics statement:** We have checked the full manuscript and moved the ethics statement to the Methods section only. All duplicate ethics-related descriptions in other sections have been completely deleted to comply with the journal's formatting requirements.
(5) **Standard funder role statement:** We have added the standard funder role statement in the cover letter as required, which confirms that the funders had no role in any stage of this study. The journal office can directly update the corresponding field in the online submission system for us.

Finally, we want to express our appreciation to the editor and reviewers. Based on the editor’s and reviewers’ comments, we have made corresponding amendments to the revised paper. We submit the revised paper and “Response to reviewers” to you.

We look forward to hearing from you soon.

Best Regards.

Corresponding author:
