# Supplementary material for: Can tiered open-source mandates optimize innovation? A differential game study osn platform vs. independent project dynamics in foundation software ecosystems
Source: PLoS One. 2026 Sep 11;21(9):e0340713. doi: 10.1371/journal.pone.0340713 (PMC13567796; doi:10.1371/journal.pone.0340713)
Supplement: Peer review history

# Peer review: Decision Letter 0

**DOI:** 10.1371/journal.pone.0340713.r001

**Authors:** Bo Li

15 Jul 2026

PONE-D-25-68439

Can Tiered Open-Source Mandates Optimize Innovation? A Differential Game Study on Platform vs. Independent Project Dynamics in AI Software

PLOS One?

Dear Dr. Yi,

Thank you for submitting your manuscript to PLOS ONE. After careful consideration, we feel that it has merit but does not fully meet PLOS ONE’s publication criteria as it currently stands. Therefore, we invite you to submit a revised version of the manuscript that addresses the points raised during the review process.

Please overhaul the paper to avoid reading like an AI-written paper. Please follow the referee comments carefully.

Please submit your revised manuscript by Aug 29 2026 11:59PM. If you will need more time than this to complete your revisions, please reply to this message or contact the journal office at plosone@plos.org. When you're ready to submit your revision, log on to https://www.editorialmanager.com/pone/ and select the 'Submissions Needing Revision' folder to locate your manuscript file.

- A letter that responds to each point raised by the academic editor and reviewer(s). You should upload this letter as a separate file labeled 'Response to Reviewers'.
- A marked-up copy of your manuscript that highlights changes made to the original version. You should upload this as a separate file labeled 'Revised Manuscript with Track Changes'.
- An unmarked version of your revised paper without tracked changes. You should upload this as a separate file labeled 'Manuscript'.

If you would like to make changes to your financial disclosure, please include your updated statement in your cover letter. Guidelines for resubmitting your figure files are available below the reviewer comments at the end of this letter.

If applicable, we recommend that you deposit your laboratory protocols in protocols.io to enhance the reproducibility of your results. Protocols.io assigns your protocol its own identifier (DOI) so that it can be cited independently in the future. For instructions see: https://journals.plos.org/plosone/s/submission-guidelines#loc-laboratory-protocols. Additionally, PLOS ONE offers an option for publishing peer-reviewed Lab Protocol articles, which describe protocols hosted on protocols.io. Read more information on sharing protocols at https://plos.org/protocols?utm_medium=editorial-email&utm_source=authorletters&utm_campaign=protocols.

As the corresponding author, your ORCID iD is verified in the submission system and will appear in the published article. PLOS supports the use of ORCID, and we encourage all coauthors to register for an ORCID iD and use it as well. Please encourage your coauthors to verify their ORCID iD within the submission system before final acceptance, as unverified ORCID iDs will not appear in the published article. *Only*  the individual author can complete the verification step; PLOS staff *cannot*  verify ORCID iDs on behalf of authors.

We look forward to receiving your revised manuscript.

Kind regards,

Bo Li, Ph.D.

Academic Editor

PLOS One

Journal Requirements:

When submitting your revision, we need you to address these additional requirements.

1. Please ensure that your manuscript meets PLOS ONE's style requirements, including those for file naming. The PLOS ONE style templates can be found at

https://journals.plos.org/plosone/s/file?id=wjVg/PLOSOne_formatting_sample_main_body.pdf and https://journals.plos.org/plosone/s/file?id=ba62/PLOSOne_formatting_sample_title_authors_affiliations.pdf

2. Your ethics statement should only appear in the Methods section of your manuscript. If your ethics statement is written in any section besides the Methods, please delete it from any other section.

3. Please provide a complete Data Availability Statement in the submission form, ensuring you include all necessary access information or a reason for why you are unable to make your data freely accessible. If your research concerns only data provided within your submission, please write "All data are in the manuscript and/or supporting information files" as your Data Availability Statement.

4. We note that the grant information you provided in the ‘Funding Information’ and ‘Financial Disclosure’ sections do not match.

When you resubmit, please ensure that you provide the correct grant numbers for the awards you received for your study in the ‘Funding Information’ section.

5. Thank you for stating the following financial disclosure:

This work was supported by the Key Research Projects in Philosophy and Social Sciences in Hunan Province [grant number 24ZDB013].

Please state what role the funders took in the study. If the funders had no role, please state: "The funders had no role in study design, data collection and analysis, decision to publish, or preparation of the manuscript."

If this statement is not correct you must amend it as needed.

Please include this amended Role of Funder statement in your cover letter; we will change the online submission form on your behalf.

6. Please update your submission to use the PLOS LaTeX template. The template and more information on our requirements for LaTeX submissions can be found at http://journals.plos.org/plosone/s/latex.

7. Please ensure that you refer to Figure 5.1 – 5.8, 6.1 – 6.8, in your text as, if accepted, production will need this reference to link the reader to the figure.

8. We note you have included a table to which you do not refer in the text of your manuscript. Please ensure that you refer to Table 5.1, 6.2-6.4 in your text; if accepted, production will need this reference to link the reader to the Table.

9. If the reviewer comments include a recommendation to cite specific previously published works, please review and evaluate these publications to determine whether they are relevant and should be cited. There is no requirement to cite these works unless the editor has indicated otherwise.

Additional Editor Comments :

The paper reads like AI-written, particularly in later sections. The author should overhaul the writing and bear all responsibility to the accuracy of the results.

[Note: HTML markup is below. Please do not edit.]

Reviewers' comments:

Reviewer's Responses to Questions

**Comments to the Author**

1. Is the manuscript technically sound, and do the data support the conclusions?

Reviewer #1: Partly

Reviewer #2: No

**********

2. Has the statistical analysis been performed appropriately and rigorously?

Reviewer #1: Yes

Reviewer #2: No

**********

3. Have the authors made all data underlying the findings in their manuscript fully available?

The PLOS Data policy

Reviewer #1: Yes

Reviewer #2: No

**********

4. Is the manuscript presented in an intelligible fashion and written in standard English?

Reviewer #1: Yes

Reviewer #2: No

**********

Reviewer #1: The paper's central result — that heterogeneous agents benefit from differentiated policy treatment — is a well-established principle in mechanism design and price discrimination theory, and the authors do not adequately distinguish their contribution from this general result. When you construct a model with two structurally different players (different costs, different private values, different spillovers) and ask whether a planner should treat them identically or differently, the answer is almost always "differently." The claimed 15–40% welfare gains and specific optimal thresholds (θ_D ≈ 60–75%, θ_I ≈ 30–40%) are artifacts of the authors' parameter calibration, which lacks empirical grounding. Section 5.1 claims calibration is "based on economic theory, empirical software engineering studies, and the strategic characteristics of platform vs. independent projects," but no citations are provided for any specific numerical value. Without empirical anchoring, the "actionable thresholds" in the abstract and conclusion are not actionable — they are outputs of assumed inputs.

Proposed fix: The authors should either (a) ground their parameter calibration in cited empirical data from real open-source AI ecosystems (e.g., contribution data from PyTorch vs. community projects, compliance cost estimates from corporate open-source program offices), or (b) reframe the contribution away from specific numerical thresholds and toward the structural conditions under which tiered mandates dominate, while explicitly acknowledging the relationship to classical results on optimal differentiation under heterogeneity.

Minor issues:

Equation 12 contains a notation error. The FOCs for the tiered case show ∂K̇_I/∂θ_u twice, but these should be ∂K̇_D/∂θ_D = 0 and ∂K̇_I/∂θ_I = 0.

The "AI software ecosystem" framing adds little. The model contains no AI-specific mechanism — no training data dynamics, no model capability curves, no compute scaling laws. The model applies equally to any two-type open-source ecosystem. The authors should either incorporate AI-specific structure or generalize the framing honestly.

The paper is substantially longer than it needs to be. The robustness section (Section 6) runs through alternative health functions, cost functions, stochastic extensions, gaming, and boundary conditions — much of which confirms what theory already predicts (differentiation helps more when agents differ more, less when they're similar). This could be cut by 40% without losing substance.

Table 5.1 mislabels the "Weak Network Effects" row: the Optimal Policy column shows π_D ≈ 0.5–0.6 (private value notation) instead of θ_D (mandate notation).

The diversity term in the ecosystem health function (Eq. 5) uses √(K_D · K_I), which is undefined when either stock is zero. The authors should discuss how the numerical algorithm handles boundary cases.

Figure 6.4's legend labels ("data1," "data2," "data3") are placeholder text that was never replaced.

Several references appear incomplete or contain formatting errors (e.g., "P´erez, 2005" in the text appears as "P´erez, 2021" in the reference list; "Almeida et al., 2019;" has a trailing semicolon followed by a closing parenthesis with no second reference).

The stochastic extension (Section 6.4) reports that under tiered mandates, K_I = 2.40 ± 1.60 versus 3.38 ± 2.27 under uniform. The independent project's mean knowledge stock is lower under tiered mandates, which contradicts the deterministic result in Section 5.2.2 (+50% gain). This discrepancy is not discussed.

The paper would benefit from a brief discussion of how the binary platform/independent classification maps to real ecosystems where projects exist on a spectrum (e.g., Hugging Face, which is VC-backed but community-driven).

For these reasons I recommend this paper be revised before publication. The model construction is competent and the analysis is thorough, but the core contribution needs sharper positioning relative to existing theory on optimal differentiation, and the parameter calibration requires empirical grounding before the claimed thresholds can be presented as policy-relevant.

Reviewer #2: A foundational logical flaw runs through the design of the follower payoff functions, as they effectively double-count the value of internally developed knowledge. Each firm’s own open-source knowledge stock is included as a component of its ecosystem benefit, treating knowledge the firm itself has created and released as if it delivers incremental external value back to the original developer. This makes little economic sense: the costs of developing that knowledge are already fully accounted for in the R&D cost term, and any private returns from undisclosed portions of the code are calculated separately. Adding one’s own contributed knowledge back as an ecosystem benefit artificially inflates perceived payoffs without any defensible theoretical justification.

The ecosystem health function suffers from a fundamental mismatch between its stated purpose and its actual mathematical structure. The quadratic term described as a penalty for imbalance between the two developer types in fact measures only the squared total of all accumulated knowledge, which captures diminishing returns to scale rather than any measure of relative disparity. A genuine penalty for structural imbalance would track the gap between the two knowledge stocks, rewarding parity and punishing extreme dominance by either side. As currently specified, the term conflates pure scale effects with distributional effects, attaching a misleading conceptual label to a mechanism that operates in an entirely different dimension.

For a framework built explicitly to capture long-run evolutionary dynamics, the model treats proprietary knowledge with surprising staticity. Only open-source knowledge accumulates as a persistent stock over time; proprietary code is modeled purely as a one-time flow of revenue that vanishes in the same period it is created. In practice, private R&D investments build durable internal knowledge capital that improves future R&D productivity and shapes long-term competitive advantage. By reducing proprietary development to a simple contemporaneous trade-off between cost and immediate revenue, the model abandons the very dynamic logic that justifies the use of a differential game in the first place.

The core rationale for tiered regulation remains theoretically underspecified, because the defining difference between platform and independent projects is never clearly isolated. The two types differ across half a dozen structural parameters simultaneously, from R&D efficiency and base operating costs to compliance burdens, monetization capacity, ecosystem dependence, and spillover sensitivity. No single dimension is identified as the essential trait of “platformness” that justifies differential treatment. This leaves the policy argument for tiering fundamentally ambiguous: readers cannot tell which source of heterogeneity drives the reported benefits, nor why tiering along the platform–independent axis is preferable to classification along any other observable dimension.

One of the paper’s central claims about tiered policy design rests on a basic misreading of the optimization structure. The paper asserts that under a tiered regime, the regulator can set each mandate rate independently to maximize the growth of its corresponding knowledge stock. This decoupling is mathematically impossible, because the ecosystem health function includes strong interaction effects between the two knowledge stocks. The marginal value of adding to one stock depends directly on the current size of the other, so the optimal rate for platform projects and the optimal rate for independent projects are jointly determined in a single coupled optimization problem. Treating them as separate, independent decisions violates the basic logic of optimal control in interconnected systems.

Formal errors in the derivation of the tiered optimum further compromise the analytical integrity of the paper. The first-order conditions presented for the tiered policy regime incorrectly retain notation from the uniform policy scenario and fail to distinguish derivatives with respect to the two separate mandate variables. This is not merely a typographical oversight: it means the algebraic step that is supposed to characterize the optimal tiered structure is formally mis-specified, and the conclusions drawn from that step cannot be taken as valid outputs of the model as written.

Several key propositions are presented as established results without the supporting derivation needed to validate them. For instance, the claim that mandate rates have an ambiguous net effect on R&D effort is stated as a general principle but never formally derived through comparative static analysis. Similarly, the discussion of strategic classification gaming posits threshold values for switching costs and verification accuracy without explicitly solving for those thresholds or proving that a stable equilibrium exists at those values. These assertions read as intuitive conjectures rather than rigorously proven results, weakening the logical chain that connects the model’s assumptions to its conclusions.

The extension to stochastic shocks includes a conclusion that contradicts a basic principle of policy instrument design. The paper suggests that when external shocks are perfectly correlated across the two project types, uniform mandates may become preferable due to unspecified risk-sharing benefits. This reasoning is unsound because a tiered policy framework strictly contains the uniform policy as a special case where both rates are set equal. A broader set of policy tools cannot produce a worse optimal outcome than a constrained subset of those same tools. There is no mechanism in the model that would generate a risk-sharing advantage for uniform rules, and the claim runs counter to fundamental optimization logic.

The numerical analysis fails to decompose the channels through which tiered mandates produce their observed effects, leaving causal claims under-supported. Simulations show that tiered rules increase the platform’s knowledge stock while also slightly raising its R&D effort, but the paper never separates the direct mechanical effect of a higher contribution requirement from the indirect incentive effect operating through changed effort levels. Given the platform’s heavier compliance burden, basic incentive logic would predict that a stricter mandate should suppress effort; any observed increase must therefore be driven by feedback from a larger shared knowledge pool. The paper does not delineate the conditions under which this feedback effect dominates or quantify its strength, instead attributing the outcome vaguely to better incentive design without closing the causal loop.

The boundary conditions presented as general policy guidelines are derived from highly restrictive single-parameter sensitivity tests, and their real-world generality is therefore overstated. Thresholds such as the required ratio of compliance costs between platform and independent projects are calculated by varying one parameter at a time while holding all others fixed at baseline values. This local comparative static exercise cannot support the paper’s tendency to present these thresholds as universal rules of thumb. When multiple parameters shift simultaneously, as they routinely do in real ecosystems, the critical thresholds will almost certainly shift as well, and the analysis provides no guidance on how or by how much.

Counterintuitive findings in the robustness analysis are left without adequate theoretical explanation. For example, the health function specification that embodies the strongest aversion to structural imbalance produces the widest gap between the two mandate rates. At first glance this seems paradoxical: greater concern for balance would intuitively call for more similar treatment, not more divergent rules. The paper brushes this result aside with a generic reference to complementarity effects but does not unpack the mechanism that would produce this pattern. Without a clearer account of why the result emerges, the robustness exercise confirms numerical sensitivity but does not deepen theoretical understanding.

The paper systematically stretches the concept of welfare in a way that inflates the apparent significance of its findings. Improvements in the ecosystem health metric are consistently described as welfare gains in the range of 15 to 40 percent, implying broad social benefits. Yet the model includes no representation of end users, consumer surplus, or broader societal outcomes; it captures only the objectives of the ecosystem architect and the two developer types. What the paper calls welfare is in fact the architect’s narrow objective function, and equating it to aggregate social welfare confuses a partial governance metric with a comprehensive welfare measure.

The paper’s claim to novel theoretical contribution is weakened by its lack of engagement with established literatures on differentiated regulation. The study positions itself as the first to endogenize tiered open-source mandates, but it does not connect its argument to the long-standing bodies of research on optimal taxation, non-linear pricing, and type screening that have analyzed policy design for heterogeneous agents for decades. It does not explain what distinctive insight the differential game setting adds beyond what classical regulation theory already provides, leaving readers uncertain about the exact nature and scope of its theoretical advance.

Foundational questions about equilibrium validity remain unaddressed throughout the analysis. The paper never explicitly specifies whether it is solving for open-loop or closed-loop feedback equilibria, and it provides no formal proof that an equilibrium exists or that it is unique. The numerical policy iteration algorithm is described step by step, but its convergence properties are not established theoretically. Without such guarantees, there is no way to rule out the possibility of multiple equilibria, unstable dynamics, or algorithmic non-convergence, all of which would cast doubt on the reliability of the reported numerical results.

There is a persistent logical gap between the model’s theoretical constructs and the practical realities of policy implementation. The core state variable driving the entire framework—effective open-source knowledge stock—is not directly measurable in real-world ecosystems. Similarly, clear, verifiable criteria for classifying projects and auditing compliance are never operationalized. Even so, the paper’s proposed adaptive governance framework calls for continuous parameter monitoring and dynamic recalibration of mandate rates. It offers no account of how policymakers would actually measure these unobservable quantities in practice, creating a rift between the model’s elegant theoretical structure and its feasibility as a real-world governance tool.

**********

what does this mean?). If published, this will include your full peer review and any attached files.

If you choose “no”, your identity will remain anonymous but your review may still be made public.

**Do you want your identity to be public for this peer review?**  For information about this choice, including consent withdrawal, please see our Privacy Policy

Reviewer #1: **Yes:**  Mark Whiting

Reviewer #2: **Yes:**  Huang, Weilun

**********

[NOTE: If reviewer comments were submitted as an attachment file, they will be attached to this email and accessible via the submission site. Please log into your account, locate the manuscript record, and check for the action link "View Attachments". If this link does not appear, there are no attachment files.]

To ensure your figures meet our technical requirements, please review our figure guidelines: https://journals.plos.org/plosone/s/figures

You may also use PLOS’s free figure tool, NAAS, to help you prepare publication quality figures: https://journals.plos.org/plosone/s/figures#loc-tools-for-figure-preparation.

NAAS will assess whether your figures meet our technical requirements by comparing each figure against our figure specifications.

# Author comment: Author response to Decision Letter 1

**DOI:** 10.1371/journal.pone.0340713.r002

19 Aug 2026

For point-by-point responses to the specific comments raised by the reviewers and the editor, please refer to our submitted system document Response to Reviewers.

Attachment Submitted filename: Response to reviewers816.docx

# Editor report: Decision Letter 1

**DOI:** 10.1371/journal.pone.0340713.r003

**Authors:** Bo Li

23 Aug 2026

Can Tiered Open-Source Mandates Optimize Innovation? A Differential Game Study on Platform vs. Independent Project Dynamics in Foundation Software Ecosystems

PONE-D-25-68439R1

Dear Dr. Yi,

We’re pleased to inform you that your manuscript has been judged scientifically suitable for publication and will be formally accepted for publication once it meets all outstanding technical requirements.

Within one week, you’ll receive an e-mail detailing the required amendments. When these have been addressed, you’ll receive a formal acceptance letter and your manuscript will be scheduled for publication.

An invoice will be generated when your article is formally accepted. Please note, if your institution has a publishing partnership with PLOS and your article meets the relevant criteria, all or part of your publication costs will be covered. Please make sure your user information is up-to-date by logging into Editorial Manager at Editorial Manager® and clicking the ‘Update My Information' link at the top of the page. For questions related to billing, please contact billing support.

If your institution or institutions have a press office, please notify them about your upcoming paper to help maximize its impact. If they’ll be preparing press materials, please inform our press team as soon as possible -- no later than 48 hours after receiving the formal acceptance. Your manuscript will remain under strict press embargo until 2 pm Eastern Time on the date of publication. For more information, please contact onepress@plos.org.

Kind regards,

Bo Li, Ph.D.

Academic Editor

PLOS One

Additional Editor Comments (optional):

Please address the following issues:

1. Clarify the theoretical contribution. The authors should further sharpen the distinction between this study and existing work on platform governance, innovation commons, and differentiated regulation. In particular, the manuscript would benefit from a clearer explanation of what new theoretical insight emerges from the dynamic Stackelberg framework.

2. Moderate the interpretation of numerical results. Some quantitative findings are presented as policy recommendations. The authors should clarify that these results are model-based implications under calibrated parameters rather than empirical estimates, and appropriately qualify the managerial and policy implications.

3. Strengthen the discussion of parameter calibration. The manuscript provides qualitative justification for parameter choices, but the basis for several numerical values remains unclear. The authors should briefly discuss how sensitive the main conclusions are to alternative calibration choices.

4. Refine the discussion of model limitations. The binary classification of platform and independent projects is acknowledged, but the authors could further explain how the results may extend to ecosystems with intermediate types of participants.

Improve presentation consistency.

5. The authors should carefully proofread the manuscript for remaining notation, formatting, and language issues, particularly in equations, figures, and references.

Reviewers' comments:

# Editor report: Acceptance letter

**DOI:** 10.1371/journal.pone.0340713.r004

**Authors:** Bo Li

PONE-D-25-68439R1

PLOS One

Dear Dr. Yi,

I'm pleased to inform you that your manuscript has been deemed suitable for publication in PLOS One. Congratulations! Your manuscript is now being handed over to our production team.

At this stage, our production department will prepare your paper for publication. This includes ensuring the following:

* All references, tables, and figures are properly cited

* All relevant supporting information is included in the manuscript submission,

* There are no issues that prevent the paper from being properly typeset

You will receive further instructions from the production team, including instructions on how to review your proof when it is ready. Please keep in mind that we are working through a large volume of accepted articles, so please give us a few days to review your paper and let you know the next and final steps.

Lastly, if your institution or institutions have a press office, please let them know about your upcoming paper now to help maximize its impact. If they'll be preparing press materials, please inform our press team within the next 48 hours. Your manuscript will remain under strict press embargo until 2 pm Eastern Time on the date of publication. For more information, please contact onepress@plos.org.

You will receive an invoice from PLOS for your publication fee after your manuscript has reached the completed accept phase. If you receive an email requesting payment before acceptance or for any other service, this may be a phishing scheme. Learn how to identify phishing emails and protect your accounts at https://explore.plos.org/phishing.

If we can help with anything else, please email us at customercare@plos.org.

Thank you for submitting your work to PLOS One and supporting open access.

Kind regards,

PLOS One Editorial Office Staff

on behalf of

Professor Bo Li

Academic Editor

PLOS One
